# Next-generation membrane-active glycopeptide antibiotics that also inhibit bacterial cell division†

**DOI:** 10.1039/d2sc05600c

**Published:** 2023-01-06

**Authors:** Paramita Sarkar, Kathakali De, Malvika Modi, Geetika Dhanda, Richa Priyadarshini, Julia E. Bandow, Jayanta Haldar

**Affiliations:** a Antimicrobial Research Laboratory, New Chemistry Unit and School of Advanced Materials, Jawaharlal Nehru Centre for Advanced Scientific Research (JNCASR) Jakkur Bengaluru 560064 Karnataka India jayanta@jncasr.ac.in +91 802208 2565; b Department of Life Sciences, School of Natural Sciences, Shiv Nadar University Dadri 201314 UP India; c Applied Microbiology, Faculty of Biology and Biotechnology, Ruhr University Bochum, Universitätsstraße 150 44780 Bochum Germany

## Abstract

Resistance to vancomycin, a life-saving drug against Gram-positive bacterial infections necessitates developing alternative therapeutics. Herein, we report vancomycin derivatives that assimilate mechanisms beyond d-Ala–d-Ala binding. The role of hydrophobicity towards the structure and function of the membrane-active vancomycin showed that alkyl-cationic substitutions favored broad-spectrum activity. The lead molecule, VanQAmC_10_ delocalized the cell division protein MinD in *Bacillus subtilis*, implying an impact on bacterial cell division. Further examination of wild-type, GFP-FtsZ, or GFP-FtsI producing- and Δ*amiAC* mutants of *Escherichia coli* revealed filamentous phenotypes and delocalization of the FtsI protein. The findings indicate that VanQAmC_10_ also inhibits bacterial cell division, a property previously unknown for glycopeptide antibiotics. The conjunction of multiple mechanisms contributes to its superior efficacy against metabolically active and inactive bacteria, wherein vancomycin is ineffective. Additionally, VanQAmC_10_ exhibits high efficacy against methicillin-resistant *Staphylococcus aureus* (MRSA) and *Acinetobacter baumannii* in mouse models of infection.

## Abstract 2

A new multi-target, multi-effect glycopeptide antibiotic that compromises membrane integrity, delocalizes cell division proteins and inhibits cell division besides inhibiting cell wall biosynthesis in both Gram-positive and Gram-negative bacteria.

## Graphical abstract

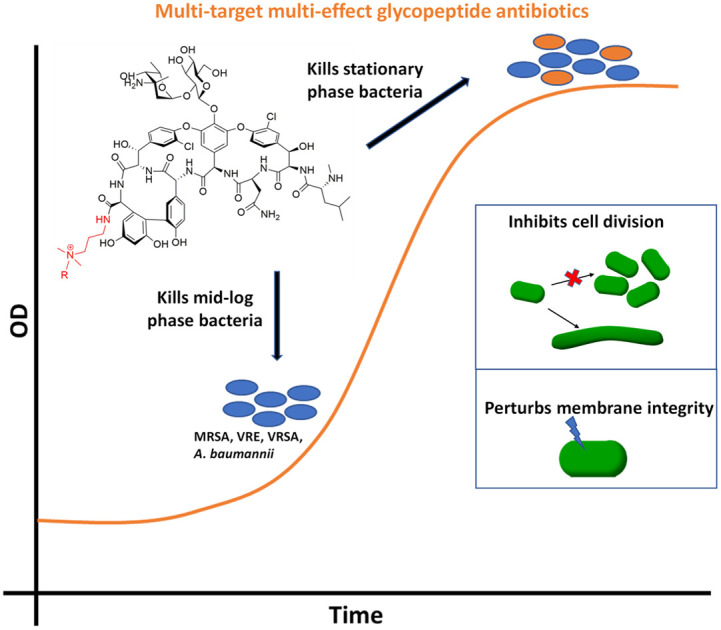

## Introduction

Vancomycin is a critically important antibiotic, that was the drug of last resort against infections caused by multidrug-resistant Gram-positive bacteria.^1^ It belongs to the glycopeptide class of antibiotics that consists of naturally occurring and semi-synthetic products that inhibit bacterial cell wall biosynthesis by binding to the d-Ala–d-Ala terminus of the cell wall precursor pentapeptide.^1,2^ However, widespread resistance to vancomycin that involves modification of the target peptide to d-Ala–d-Lac/d-Ser, concomitant with a reduction in binding affinity, and/or thickening of the cell wall has been reported.^3^ To incorporate additional mechanisms, second-generation glycopeptide antibiotics, telavancin, dalbavancin, and oritavancin, were equipped with lipophilic substitutions that abetted destabilization of the bacterial membranes.^4^ Despite reports of various modified glycopeptides that overcome inherited resistance,^3,5–10^ there are only a few reports of derivatives that are effective against Gram-negative bacteria and dormant bacteria.^11–15^ The presence of an outer membrane in Gram-negative bacteria (GNB) precludes entry of numerous antibiotics including glycopeptides.^16^ Although the conjugation of membrane-interacting moieties onto glycopeptide antibiotics has shown activity against vancomycin-resistant Gram-positive bacteria, they do not necessarily result in activity against Gram-negative bacteria.^17^

We had developed C-terminally modified cationic lipophilic vancomycin derivatives that were effective against both vancomycin-resistant Gram-positive and the intrinsically resistant Gram-negative bacteria.^18–20^ Their broad-spectrum antibacterial activity was attributed to the incorporation of membrane–perturbation properties through the conjugation of lipophilic cationic moieties. In this report, we first examine the structure–activity relationship of alkyl and aryl moieties as hydrophobic substituents and identify the most selective compound. The effect of various hydrophobic group substitutions on membrane perturbing ability, the antibacterial activity of exponential growth, and quiescent bacteria was therefore examined to gain insights for better designs. Of most significance, here, a study of the effect of the lead compound on the membrane, cell division, and associated proteins was performed to better understand the modes of action of this new semisynthetic glycopeptide antibiotic. The protein localization and morphological changes were evaluated upon treatment of *B. subtilis* producing GFP-MinD protein, *E. coli* producing various GFP-tagged cell division proteins and an amidase lacking mutant. Further, the potential of the lead compound as a preclinical candidate has been demonstrated in the murine thigh infection model against MRSA and chronic burn-wound infection against *A. baumannii*.

## Results

### Structure–activity relationship study

#### Design rationale

**Fig. 1:**
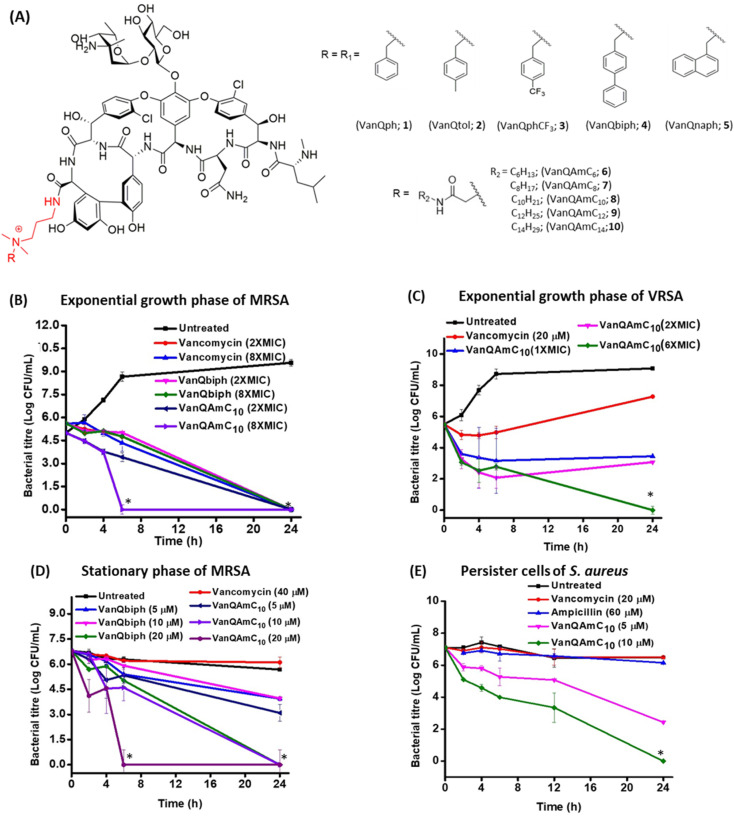
(A) Structure of cationic lipophilic vancomycin derivatives. Bactericidal kinetics of (B) VanQbiph and VanQAmC_10_ against exponential growth phase MRSA, (C) VanQAmC_10_ against VRSA, (D) VanQbiph and VanQAmC_10_ against stationary phase cells of MRSA, and (E) VanQAmC_10_ against persister cells of *S. aureus*. Against MRSA, MIC of VanQAmC_10_ = 0.4 μM, MIC of VanQbiph = 0.5 μM; MIC of VanQAmC_10_ against VRSA = 1.6 μM and ‘_*_’ indicates <50 CFU mL^−1^.

The second-generation glycopeptide antibiotics dalbavancin, oritavancin and possess hydrophobic moieties conjugated to the vancosamine sugar.^21–23^ These antibiotics display improved activity against vancomycin-resistant bacteria. However, they are not active against Gram-negative bacteria. We had previously reported that the attachment of cationic lipophilic moieties onto the C-terminus of vancomycin results in interaction with the negatively charged bacterial membrane.^15^ However, compounds with activity against both Gram-positive and Gram-negative bacteria were also slightly toxic. To address this, two sets of derivatives with C-terminus amido-alkyl-cationic (1–5) and aryl-cationic (6–10) substitutions were designed (Fig. 1A) and synthesized (Scheme S1†). The amphiphilicity of the cationic lipophilic moiety can be varied to selectively target the anionic bacterial membrane over the zwitterionic mammalian membrane. Biophysical studies and molecular dynamics simulation studies in polymeric and small molecular systems indicated that the inclusion of an amide spacer between the quaternary ammonium moiety and the hydrophobic moiety enhances the selectivity towards bacterial cells over mammalian cells.^24,25^ The inclusion of an amide bond contributes to additional hydrogen bonding capacity to the bacterial lipids.^26^ It was, therefore, envisioned that lipophilic cationic vancomycin derivatives with an amide spacer could result in improved selectivity towards bacteria.

#### Activity against Gram-positive bacteria

The antibacterial activity of vancomycin derivatives was determined as the lowest concentration required to completely inhibit bacterial growth (minimum inhibitory concentration, MIC). Against MRSA, the aryl-substituted derivatives 1–5 showed activity similar to vancomycin (Table 1). The increase in the carbon content of substituted moieties from six (1) to seven (2 and 3) to ten (5) to twelve (4), increased activity. Among these derivatives, the biphenyl-substituted, VanQbiph (4) was the most effective. It showed a 22 to 88-fold increase in activity as compared to vancomycin against VRSA. Against VRE, it showed a 40- to 60-fold enhancement in activity.

**Table 1:** Antibacterial activity and hemolysis of vancomycin derivatives (1–10) against multi-drug-resistant Gram-positive and Gram-negative bacteria^a^

Compound	Minimum inhibitory concentration (μM)									
	Gram-positive bacteria						Gram-negative bacteria			
	MRSA	VRSA 1	VRSA 4	VRSA 12	VRE 51575	VRE 51559	AB 1425	PA R590	AB R674	HC_50_ (μM)
Vancomycin	0.6	345	345	345	750	250	100	100	100	N.D
VanQph (1)	1	>30	>30	>30	>30	>30	>30	>30	25.5	>500
VanQtol (2)	1	>30	16	>30	>30	>30	>30	>30	25.3	>500
VanQphCF_3_ (3)	1	>30	15.8	>30	>30	>30	>30	24.6	24.6	>500
VanQbiph (4)	0.5	7.8	3.9	15.7	12.3	6.1	15.7	>30	24.5	>500
VanQnaph (5)	1	>30	3.9	>30	25	25	>30	>30	24.8	>500
VanQAmC_6_ (6)	0.8	>30	6.3	>30	>30	25	25	12.5	12.5	>500
VanQAmC_8_ (7)	0.8	3.1	3.1	>30	25	12.5	12.5	6.3	12.5	>500
VanQAmC_10_ (8)	0.4	3.1	1.6–3.1	1.6	4	4	6.3	3.1	10	>500
VanQAmC_12_ (9)	3.12	1.5	0.7	3	3.1	1.5	6.3	6.3	6.3	350
VanQAmC_14_ (10)	6.25	3	1.5	3	3.1	1.5	6.3	6.3	12.5	150

a MRSA, methicillin-resistant *S. aureus* (ATCC 33591); VRSA, vancomycin-resistant *S. aureus*; VRE vancomycin-resistant *E. faecium* (VanA phenotype, ATCC 51559); vancomycin-resistant *E. faecalis* (VanB phenotype, ATCC 51575); ND, not determined. MIC against VRE and VRSA, HC_50_ of VanQAmC_10_ was previously reported.^18^

Among the amido-alkyl substituted compounds, 6–8 exhibited MICs similar to that of vancomycin against MRSA while the longer chain variants, 9 and 10 showed reduced activity. Against VRSA, the lower chain length variants, 6 and 7 were less effective; VanQAmC_10_ (8) showed a significant 115–490-fold improvement in activity as compared to vancomycin. 9 and 10 had similar MICs and were slightly more effective than 8. Against VRE, VanQAmC_10_ (8) showed activity similar to that against VRSA (∼160-fold improvement in activity as compared to vancomycin). In general, the series of amido-alkyl derivatives, 6–10 exhibited an increase in activity with longer chains, with no further enhancement in activity between longer chain substituted derivatives 9 and 10. The amido-alkyl chain containing compounds exhibited better activity than those with aromatic substitutions. This indicated that the more flexible hydrophobic alkyl chain moieties resulted in better activity. VanQbiph (4), VanQAmC_10_ (8), and VanQAmC_12_ (9) demonstrate potency against vancomycin-resistant Gram-positive bacteria and were potential lead candidates. VanQAmC_10_ shows activity similar to telavancin and is better than dalbavancin. The MIC of semi-synthetic glycopeptides such as oritavancin, telavancin and dalbavancin against VRE (VanA phenotype) were reported as 0.14 μM, 4 μM and 18 μM respectively.^4,20,41^

#### Activity against Gram-negative bacteria

While the aromatic hydrophobicity containing compounds 1–5 did not show significant activity, the alkyl-containing derivatives (6–10) were active against *A. baumannii* (Table 1). The activity against *P. aeruginosa* was strain-dependent, and the compounds were inactive against *K. pneumoniae*. Against *A. baumannii* (MTCC 1425 and AB R674), 6 and 7 were moderately active, while 8–10 showed improved activity with an MIC value of 6.3 μM. Against an MDR strain of *P. aeruginosa*8–10 exhibited the highest activity. In general, compounds with higher hydrophobicity, 8–10, displayed similar activity against Gram-negative bacteria. The results indicate that alkyl-cationic moieties are necessary for the antibacterial activity of vancomycin derivatives against Gram-negative bacteria. Of these, VanQAmC_10_ (8) and VanQAmC_12_ (9) demonstrated the highest activity against both Gram-positive and Gram-negative bacteria.

#### Hemolytic activity

To select the lead compounds, the toxicity of 1–10 was tested against human erythrocytes as their hemolytic activity (HC_50_). The HC_50_ is determined as the concentration at which 50% of the compound treated cells are lysed. The compounds with aromatic substitutions, 1–5, possessed lower antibacterial activity and were also non-toxic (Table 1). The HC_50_ for compounds 6–8 was also greater than 500 μM. Compounds 9 and 10 with longer chain length caused hemolysis. Based on the *in vitro* activity and hemolysis studies, VanQbiph (4) from the aryl-substituted compounds and VanQAmC_10_ (8) from the alkyl-chain substituted compounds were selected for further investigations. VanQAmC_10_ (8) was found to be non-toxic to MDCK cells with CC_50_ >64 μM (Fig. S1†). VanQAmC_10_ (8) was additionally non-toxic to RAW 264.7 cells up to 40 μM and was therefore taken forward *in vivo* investigation.^18^

### Eradication of exponentially growing bacteria

To examine the antibacterial properties of the two leads, VanQbiph (4) and VanQAmC_10_ (8), the kinetics of bactericidal activity against exponentially growing, log-phase cells of MRSA was evaluated (Fig. 1B). The minimum bactericidal concentration (MBC) of VanQbiph (4) and VanQAmC_10_ (8) was at 2×MIC. VanQAmC_10_ (8) showed faster killing than both VanQbiph (4) and vancomycin. At 2×MIC, both the compounds exhibited complete eradication in 24 h like vancomycin. At 8×MIC, VanQAmC_10_ (8) showed complete eradication within 6 h while VanQbiph (4) showed a similar effect at 24 h. VanQAmC_10_ (8) showed a time and concentration-dependent activity while VanQbiph (4) and vancomycin showed only a time-dependent activity.

Against VRSA, VanQAmC_10_ (8) exhibited better efficacy than VanQbiph (4) and was therefore tested for kinetics of killing. At MIC, it reduced the bacterial titer by 1.9 log CFU mL^−1^ in 24 h (Fig. 1C). At 2×MIC, an initial reduction was observed, followed by a 1 log CFU mL^−1^ increase in bacterial titer between 6 h and 24 h. At 6×MIC VanQAmC_10_ resulted in complete eradication after 24 h. Treatment with vancomycin at 20 μM (sub-MIC), showed an initial growth inhibitory effect, followed by resumption of bacterial growth. Resistance in VRSA results from a combination of target modification as well as thickening of the cell wall. The additional mechanisms of VanQAmC_10_ possibly contribute to its bactericidal activity against VRSA.

### Eradication of metabolically inactive bacteria

The stationary phase cells and persister cells are metabolically repressed and phenotypically different from the log-phase cells.^27^ For activity, the commonly used drugs, vancomycin, and linezolid require actively ongoing cellular processes. They are therefore ineffective against non-dividing bacteria. Even at the concentration of 40 μM, vancomycin did not reduce the number of viable stationary phase cells of MRSA in 24 h (Fig. 1D). The integrity of the bacterial membrane is imperative to survival irrespective of metabolic state. VanQbiph (4) and VanQAmC_10_ (8) were designed to incorporate membrane activity and therefore expected to be effective against the metabolically inactive cells of MRSA. VanQAmC_10_ (8) showed better activity than VanQbiph (4) at the same concentration, similar to that observed against log-phase cells of MRSA. Irrespective of treatment concentration, VanQbiph (4), exhibited slow killing with no significant reduction in viability up to 6 h. Complete eradication was observed at a higher concentration of 20 μM in 24 h. VanQAmC_10_ (8) showed rapid bactericidal activity that increased with concentration. It completely eradicated cells in 24 h at 10 μM and within 6 h at 20 μM.

Persister cells are a subpopulation of bacterial cultures that are refractory to antibiotic treatment.^28^ Since VanQAmC_10_ (8) was more effective than VanQbiph (4) against stationary phase cultures, its activity against persister cells was evaluated. VanQAmC_10_ (8) exhibited a concentration-dependent bactericidal activity against the persister cells of MRSA (Fig. 1D). This activity was more rapid than that observed against stationary phase cells.

Another growing challenge in treating bacterial infections is the formation of biofilms, which are recalcitrant to antibiotic treatment.^29^ Most antibiotics including vancomycin are rendered ineffective against biofilms consisting of both dividing and non-dividing cells, majorly due to their inability to penetrate through the extracellular polymeric matrix. Thus, it is important to develop compounds that eradicate biofilms as well as planktonic cells. Untreated biofilms of MRSA grew to a thickness of 11.8 μm (Fig. S3A†). The vancomycin-treated biofilms remained intact with thickness similar to the untreated control. VanQAmC_10_ (2) was found to reduce the thickness of biofilms of MRSA by about 40%, showing a thickness of 7.4 μm as opposed to 11.8 and 10.1 μm for untreated and vancomycin treated cases respectively (Fig. S3B and C†).

### Mechanism of action against Gram-positive bacteria

#### Membrane depolarisation

**Fig. 2:**
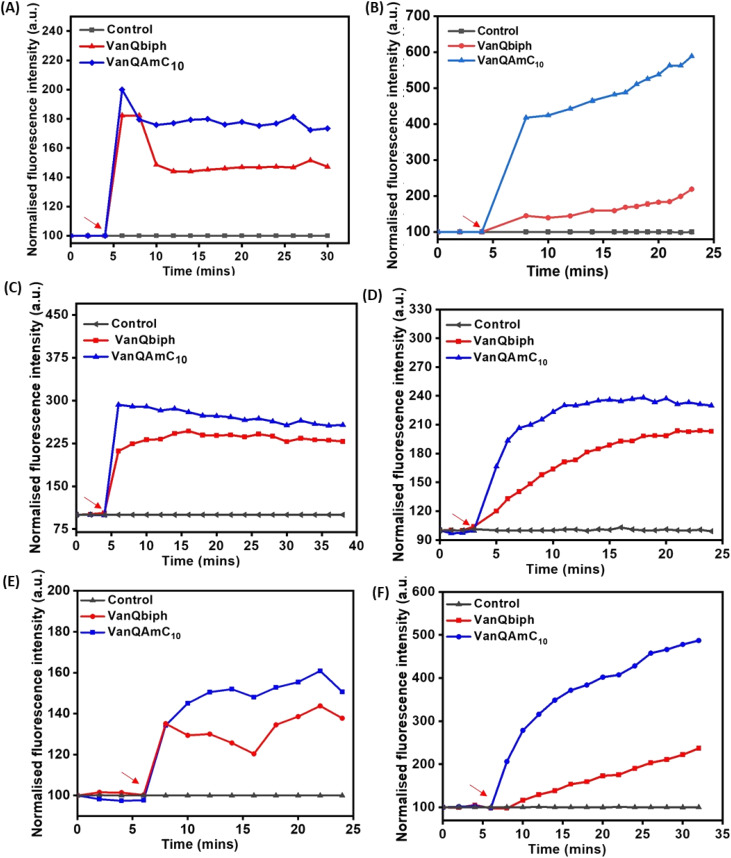
Membrane–perturbation properties of VanQbiph and VanQAmC_10_. Membrane depolarization against, (A) exponentially growing MRSA, (B) exponentially growing *B. subtilis*, upon treatment at 10 μM, and (C) stationary phase cells of MRSA upon treatment at 20 μM. Membrane permeabilization against, (D) exponentially growing MRSA, (E) exponentially growing *B. subtilis*, upon treatment at 10 μM, and (F) stationary phase cells of MRSA upon treatment at 20 μM. Red arrows indicate compound addition. The error percentage between replicates of an experiment was lesser than 5%. The images are representative of results from three independent experiments.

Having established the superior antibacterial properties of cationic lipophilic vancomycin derivatives, their modes of action were probed. The membrane disruption properties of both VanQbiph (4) and VanQAmC_10_ (8) were studied to correlate the extent of membrane perturbation and bactericidal activity against both exponentially growing and non-growing bacterial cells. Their ability to depolarize the membrane was monitored using the fluorescent probe DiSC_3_(5) (3,3′-dipropylthiadicarbocyanine iodide). DiSC_3_(5) is sensitive to the membrane potential and as it accumulates in the membrane, the fluorescence intensity decreases due to self-quenching. Upon dissipation of membrane potential, an increase in fluorescence is observed due to DiSC_3_(5) being dispersed in the solution. Against *B. subtilis* and MRSA, both the compounds VanQbiph (4) and VanQAmC_10_ (8) depolarised the membrane within 2 minutes post-treatment at 10 μM (Fig. 2A and B). The depolarization occurred in a concentration-dependent manner (Fig. S2A†). The alkyl chain substituted compound, VanQAmC_10_ (8) showed a higher extent of depolarisation than the aromatic biphenyl substituted, VanQbiph (4). Against stationary phase cells of MRSA also, rapid dissipation of membrane potential was observed within 2 minutes post-treatment with both VanQbiph (4) and VanQAmC_10_ (8) (Fig. 2C). Vancomycin did not affect the membrane potential.

#### Membrane permeabilization

The kinetics of permeabilization of membranes of MRSA and *B. subtilis* by the compounds was measured by the uptake of propidium iodide (PI). The dye, PI does not permeate through intact membranes. When the membrane integrity is compromised, it enters the cell and fluoresces upon binding to the DNA. Both VanQbiph (4) and VanQAmC_10_ (8) permeabilized the membrane in a concentration-dependent manner (Fig. S2†). While vancomycin does not affect the membrane integrity, VanQAmC_10_ (8) exhibited rapid permeabilization within 4 minutes of treatment. VanQbiph (4) showed slower permeabilization (Fig. 2D and E). They caused gradual permeabilization against the stationary phase cells of MRSA as well (Fig. 2F). The extent of permeabilization was higher for VanQAmC_10_ (8) than VanQbiph (4) against both the log-phase and stationary-phase bacteria similar to that observed during depolarisation. Membrane-perturbation may be the predominant mechanism leading to the antibacterial activity against metabolically inactive bacteria.

**Fig. 3:**
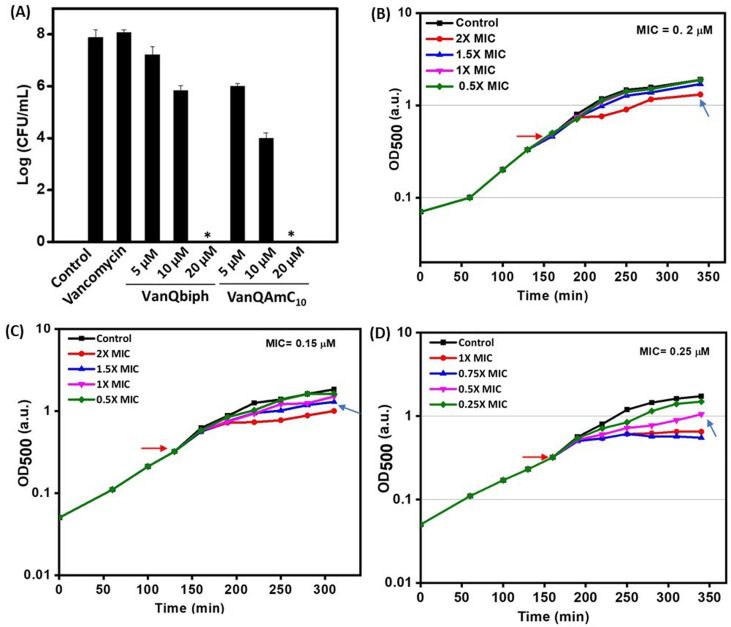
(A) Viability of planktonic cells post-treatment in HEPES-glucose; acute growth retardation upon treatment with (B) vancomycin, (C) VanQbiph, (D) VanQAmC_10_. ‘_*_’ indicates <50 CFU mL^−1^. Red arrow indicates compound addition and blue arrow indicates PEC.

To confirm that membrane perturbation contributed to cell death, the bacterial viability was evaluated under conditions similar to the mechanistic studies. A decrease in the number of viable cells was observed with an increase in the concentration of both VanQbiph (4) and VanQAmC_10_ (8) (Fig. 3A). The number of viable cells was lower upon treatment with VanQAmC_10_ (8) than when treated with VanQbiph (4) at the same concentration. At 5 μM and 10 μM, VanQbiph (4) showed 0.8 log CFU mL^−1^ and 2.2 log CFU mL^−1^ reduction respectively. VanQAmC_10_ (8) showed 2 log CFU mL^−1^ and 4 log CFU mL^−1^ reduction upon treatment at 5 μM and 10 μM respectively. Treatment with both the compounds at 20 μM resulted in complete eradication of the bacteria. Vancomycin did not reduce the viability of bacteria even at 40 μM, under the same conditions. The stronger membrane–perturbation properties of VanQAmC_10_ (8) therefore contribute to higher activity against both exponentially growing and stationary phase bacteria.

#### Acute effect on bacterial growth

The mechanisms of action in Gram-positive bacteria were further examined in the model bacterium *B. subtilis*. The study of the mechanism of action of a drug requires live cells under antibiotic stress. A 30–50% growth inhibition upon treatment, triggers a stress response in the bacterial cell while allowing cell proliferation to progress at a level sufficient to study the effect of the compound. The lowest concentration at which this effect was observed, was termed the physiologically effective concentration (PEC). The MIC of vancomycin, VanQbiph (4), and VanQAmC_10_ (8) against *B. subtilis* were 0.2 μM, 0.15 μM, and 0.25 μM, respectively. The acute effect of different antibiotic concentrations on exponentially growing bacterial cultures were tested to identify the PEC (Fig. 3B–D). Vancomycin showed growth retardation at 0.4 μM. Similar growth retardation was observed at lower concentrations for both compounds. VanQAmC_10_ (8) showed an inhibitory effect from 0.125 μM, while VanQbiph (4) showed a similar inhibition effect from 0.2 μM. The presence of cationic lipophilic moieties possibly result in higher accumulation in the membrane region leading to growth inhibition at lower concentrations.

#### Cell wall biosynthesis inhibition

**Fig. 4:**
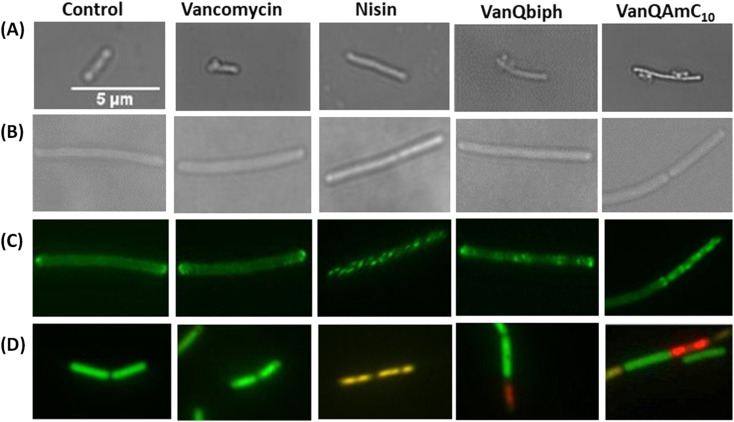
Examination of the effect of VanQbiph, VanQAmC_10_, vancomycin and nisin on cell membrane and wall integrity of *B. subtilis* upon treatment at respective PECs. (A) Inhibition of cell wall biosynthesis and compromise of cell wall integrity by visualization of ‘bubbles’ on the bacterial surface post acetic acid/methanol fixation through light microscopy; (B) light microscopy to assess the morphology of GFP-MinD expressing *B. subtilis* cells; (C) delocalization of GFP-MinD through fluorescence microscopy; (D) membrane permeabilization and pore formation monitored through fluorescence with the BacLight bacterial viability kit. Scale of all images is the same (5 μm).

To further study the mechanisms contributing to the growth retardation, microscopic studies were carried out upon treatment of *B. subtilis* with compounds. Upon inhibition of cell wall biosynthesis, holes are formed in the peptidoglycan layer where new cell wall material is no longer incorporated. The cytoplasmic membrane extrudes out of these perforations, appearing as bubbles on the cell surface when treated with a 1 : 3 mixture of acetic acid and methanol.^30^ This phenomenon is exhibited by compounds that inhibit cell wall biosyntheses like vancomycin and not agents that only perturb the membrane integrity such as gramicidin-A and valinomycin.^31^ Like vancomycin, VanQbiph (4) and VanQAmC_10_ (8) showed bubbles on the surface, indicating that they inhibit cell wall biosynthesis and compromise the integrity of the cell wall (Fig. 4A).

#### MinD delocalization

MinD is a peripheral membrane protein that localizes at the cell poles and is part of the cell division regulation machinery. It has been reported that treatment with membrane-depolarizing agents like valinomycin results in the delocalization of the protein which appears in irregularly distributed spots throughout the cell. Since the compounds depolarise the bacterial membrane, their effect on the localization of the GFP-tagged MinD protein of *B. subtilis* was investigated. Treatment with VanQbiph (4) and VanQAmC_10_ (8) at the PEC resulted in an irregular distribution of GFP-labelled MinD across the cells (Fig. 4B and C). This indicates that the compound also possibly stalls the bacterial cell division process.

#### Membrane permeabilization without pore formation

To examine if the compounds form pores on the membrane, a mixture of the fluorescent dyes SYTO 9 and PI was used. While SYTO 9 penetrates both intact and permeabilized cells staining them green, PI is unable to cross the intact membrane and stains only permeabilized or dead cells red. Membrane-disrupting antibiotics such as nisin are known to form pores in the membrane is stained by both SYTO 9 and PI (Fig. 4D). However, upon treatment with VanQbiph (4) and VanQAmC_10_ (8), cells did not co-stain cells with both the dyes, indicating non-specific interaction with the membrane. Around 20% of the cells visualized post-treatment with both compounds were stained by PI and therefore permeabilized.

#### Antagonization assay with *N*,*N*′-diacetyl-l-Lys–d-Ala–d-Ala (Ac_2_KAA) and teichoic acid

Ac_2_KAA can act as a competitive ligand to the target of glycopeptide antibiotics and therefore antagonize antibacterial activity. In the presence of 500 μM of Ac_2_KAA, the activity of VanQAmC_10_ (8) was reduced by 2-fold. However, the MIC of vancomycin increased from 0.6 μM to >30 μM (>40-fold increase in MIC) in presence of the same amount of Ac_2_KAA. The 2-fold reduction in the activity of VanQAmC_10_ (8) accounts for the loss of activity due to d-Ala–d-Ala binding. This implied that it acted through additional mechanisms that are independent of Ala-d-Ala binding. The presence of the positive charge in VanQAmC_10_ (8) could result in interactions with the negatively charged components of the bacterial cell wall like teichoic acid. C-terminal trimethyl ammonium modified vancomycin derivative has been reported to bind to teichoic acid.^32^ However, no change in the MIC of both vancomycin and VanQAmC_10_ was observed in presence of lipoteichoic acid as a competing ligand, implying that it was not a target.

### Mechanism of action in Gram-negative bacteria (*E. coli*)

#### Lysis of *E. coli*

**Fig. 5:**
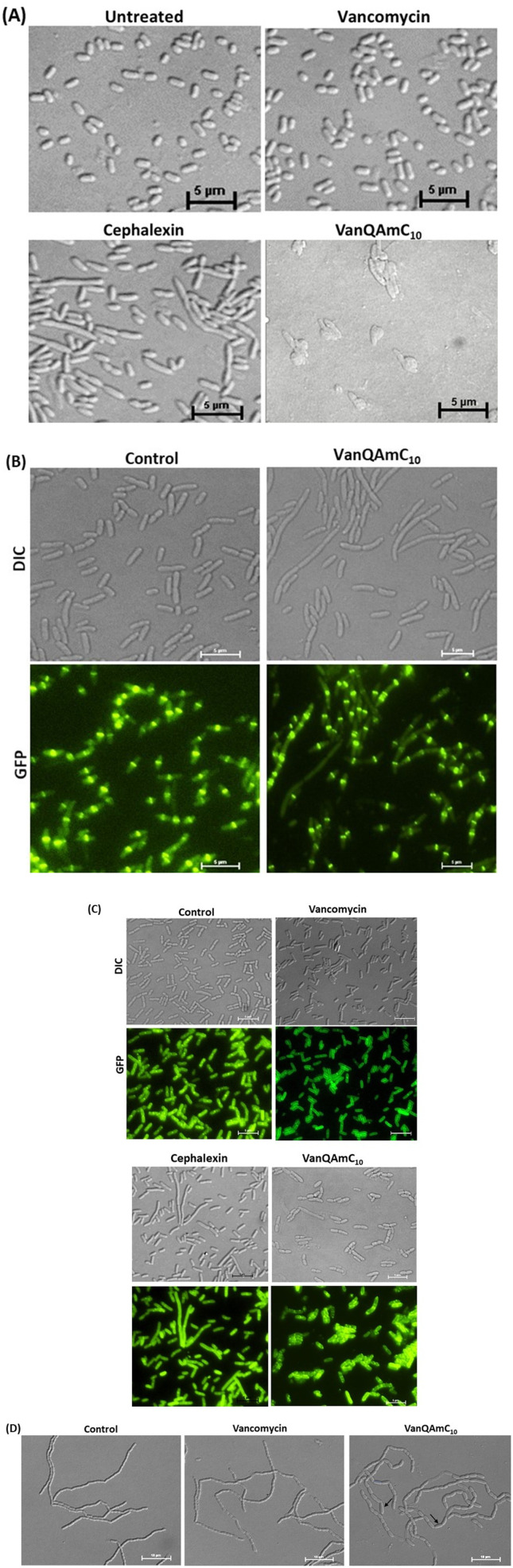
Examination of E. coli cells treated with vancomycin, cephalexin and VanQAmC10 to study the effect on bacterial membrane integrity and cell division. (A) Light microscopy of wild-type E. coli upon treatment with vancomycin (34 μM), and VanQAmC10 (25 μM); (B) light and fluorescence microscopy in GFP-FtsZ expressing E. coli to assess morphological changes and localization of FtsZ post-treatment with VanQAmC10 (15 μM) for 40 min; (C) delocalization of GFP-FtsI in GFP-FtsI expressing E. coli through fluorescence microscopy and morphological changes through light microscopy upon treatment with vancomycin (34 μM), cephalexin (22 μM) and VanQAmC10 (10 μM); (D) light microscopy to assess morphological changes in E. coli MG1655 Δ*amiAC* mutant, when untreated and treated with vancomycin (3 μM) and VanQAmC10 (2 μM). Black arrows indicate morphological aberrations.

The MIC of VanQAmC_10_ (8) against *E. coli* was 16 μM whereas vancomycin was inactive up to 66 μM. Microscopic examination of *E. coli* treated with VanQAmC_10_ at 25 μM, showed that it completely lysed the cells, while vancomycin did not affect the cells (Fig. 5A). Over 240 minutes, *E. coli* treated with VanQAmC_10_ (at 25 μM), gradually grow bulky before finally lysing (ESI Video†). It punctured the cell, causing the cytoplasm to leak out as extrusions on the surface of the bacteria after which the cells flattened and disintegrated. Previous studies in Gram-negative bacteria indicated that VanQAmC_10_ (8) can depolarise and permeabilize their outer and inner membrane.^18^

#### Effect on bacterial cell division

The division process broadly involves the following stages, (i) marking of the division site; (ii) employing the divisome and constriction of the cell wall through activation of cell wall synthesis; (iii) membrane fusion and cell wall hydrolysis for compartmentalization and physical separation into daughter cells.^33^ It was envisioned that the membrane–perturbation properties of VanQAmC_10_ (8) could affect bacterial cell division and the localization of associated proteins. Its effect on the various stages of cell division was investigated. In the first stage of cell division in bacteria, the particular localization of FtsZ at the septum is crucial. The effect of antibiotic treatment on localization and morphology against GFP-FtsZ producing bacteria was therefore first inspected.

#### Inhibition of cell division in GFP-FtsZ producing *E. coli*

The FtsZ protein assembles into a membrane-associated ring structure at the division septum. It recruits other proteins for the progression and completion of cell division.^34^ The effect of VanQAmC_10_ on the localization of FtsZ and morphology in *E. coli* producing green fluorescent protein (GFP)-tagged FtsZ was studied through microscopy (Fig. 5B). The cells were treated with IPTG to overexpress *gfp-ftsZ* and then either treated with compounds or left untreated. Upon treatment with 15 μM of VanQAmC_10_, the cells appear to be filamentous with variable lengths of up to 4 μm, while cells in the control group were ∼2.5 μm long. Incubation with VanQAmC_10_ for a longer time (130 minutes) results in bulkier filamentous cells, with severely distorted shapes (Fig. S4†). However, the localization and distribution of Z-rings remain unaltered. IPTG-treated cells formed minicells of ∼1.5 μm length, which is characteristic of cells overproducing FtsZ.^35^ Known inhibitors of FtsZ alter the regular midcell distribution of Z-rings.^36^ The results indicate that VanQAmC_10_ either affects FtsZ differently than the known inhibitors or that it inhibits bacterial cell division indirectly.

#### Mislocalization of GFP-FtsI protein

FtsI or PBP-3 is the only transpeptidase required for bacterial cell division and localizes to the septal ring.^37^ It regulates the degree of cross-linking and coordinates the division process. GFP-tagged FtsI producing *E. coli* cells were treated with vancomycin (at 34 μM) and VanQAmC_10_ (at 10 μM and 15 μM; Fig. 5C) and then examined under the microscope. The untreated and vancomycin-treated cells showed green fluorescence due to the localization of FtsI at the septum. Cephalexin, a known inhibitor of FtsI, induces a filamentous phenotype with mislocalized GFP-FtsI protein.^38^ Upon treatment with 10 μM of VanQAmC_10_, larger phenotypes of variable sizes are observed with shape defects. Some cells appear filamentous like in the case of cephalexin. The GFP-FtsI protein appears as green fluorescent spots delocalized across the cell in 60% of the cells visualized. At a higher concentration of 15 μM, bulkier cells with more distorted shapes than those at 10 μM were observed (Fig. S5†). Thus, VanQAmC_10_ mislocalizes the cell division protein, FtsI, and inhibits further cell division.

#### Sensitivity against mutants lacking AmiA and AmiC

The bacterial amidases help cleave the septum in the final stage of cell division to produce daughter cells.^39^ In the absence of amidases, chains of unseparated cells are formed (Fig. 5D). These retain distinct cytoplasmic compartments but share an outer membrane.^39^ These cells are known to be hypersensitive to antibiotics and detergents. However, Δ*amiAC* mutations are resistant to killing by cephalexin.^38^ The MIC of VanQAmC_10_ against the Δ*amiAC* mutant was reduced to 4 μM, while that of vancomycin was >10 μM. This indicates that VanQAmC_10_ inhibits cell division through mechanisms different from cephalexin. Microscopic examination of the mutants treated with sub-inhibitory concentrations of VanQAmC_10_ revealed larger cell phenotypes of variable sizes. This is indicative of impaired cell division. Cell debris due to lysis after compound treatment are also visible. In the vancomycin-treated cells, few cells appear larger and bulky while most appear similar to the untreated bacteria (Fig. 5D).

### *In vivo* activity of VanQAmC_10_

#### Resistance induction

**Fig. 6:**
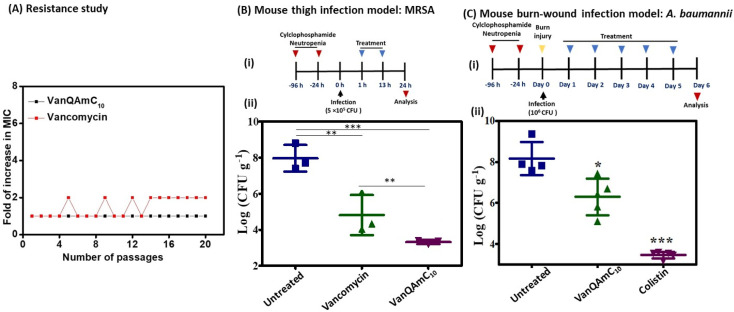
(A) Propensity of vancomycin and VanQAmC_10_ to induce resistance in MRSA upon serial exposure to sub-MIC concentrations; (B) (i) experimental design for *in vivo* efficacy study in a murine thigh infection model, (ii) *in vivo* efficacy of VanQAmC_10_ and vancomycin against the multidrug-resistant methicillin-resistant *S. aureus* (MRSA) (*n* = 3/dose). Antibiotics were administered intraperitoneally twice at 12 h intervals at a dose of 12 mg kg^−1^. (C) (i) Experimental design for *in vivo* efficacy study in murine burn wound infection model, (ii) *in vivo* efficacy of VanQAmC_10_ and colistin against the carbapenem-resistant *A. baumannii* (*n* = 5/dose, 1 mouse died in untreated control). VanQAmC_10_ and colistin were treated topically at 30 mg kg^−1^ for 5 days (‘***’ indicates *p* < 0.0001, ‘**’ indicates *p* < 0.001, ‘*’ *p* < 0.05, *p* = 0.08 (n.s.) for VanQAmC_10_ with respect to vancomycin).

Having observed the multiple mechanisms of VanQAmC_10_, the potential as a preclinical candidate was examined. The multimodal mechanisms possibly lead to the lack of resistance development to VanQAmC_10_ in MRSA after 20 passages, while vancomycin showed a 2-fold increase in MIC (Fig. 6A). Additionally, VanQAmC_10_ does not induce resistance in *A. baumannii*, unlike colistin.^18^ The frequency of resistance for both vancomycin and VanQAmC_10_ against MRSA was <10^−8^ indicating the absence of spontaneous mutants.

#### Activity in mouse liver homogenate and human plasma

The MIC of VanQAmC_10_ against MRSA and VRE remained unchanged in both plasma as well as liver homogenate, thereby confirming their stability for activity *in vivo* (Table S1†).

#### Efficacy in mouse infection models

The LD_50_ of VanQAmC_10_ (8) was found to be 70 mg kg^−1^ through intravenous injection.^18^ When administered intraperitoneally, a 130 mg kg^−1^ dose was found to be well tolerated and all mice survived. The LD_50_ of VanQAmC_10_ was greater than 160 mg kg^−1^ when administered subcutaneously. The *in vitro* activity, low toxicity, and stability in plasma and liver homogenate, supported the potential of VanQAmC_10_ as a candidate for the treatment of bacterial infections. The efficacy of VanQAmC_10_ (8) against MRSA was tested in a neutropenic mouse thigh infection model. Infection was established by injecting them with 5 × 10^5^ CFU of MRSA in the thigh. 1 h and 13 h post-infection, mice were treated with 12 mg kg^−1^ of vancomycin and VanQAmC_10_ administered intraperitoneally (Fig. 6B). The mice were sacrificed 24 h post-infection. The pre-treatment bacterial load was found to be 6.3 log CFU mL^−1^. In the untreated group, the bacterial load increased to 8 log CFU g^−1^. The vancomycin treated group showed a bacterial load of 4.8 log CFU g^−1^ of tissue. However, treatment with VanQAmC_10_ reduced the bacterial load to 3.3 log CFU g^−1^, which is a 1.5 log CFU g^−1^ lower bacterial load than observed against vancomycin. The results highlight the superior *in vivo* efficacy of VanQAmC_10_ as compared to that of the parent drug vancomycin.

Further, the efficacy of VanQAmC_10_ (8) was tested in a chronic burn-wound infection model of *A. baumannii*.^40^ Chronic burn wounds on the back of mice were infected with *A. baumannii*. 24 h post-infection, before initiation of treatment, the bacterial load was found to be 7.5 log CFU g^−1^. Infected mice were treated with 30 mg kg^−1^ of VanQAmC_10_ and colistin for five days consecutively. The bacterial load in the untreated group increased to 8 log CFU g^−1^ six days post-infection. Treatment with VanQAmC_10_ resulted in a 2.2 log CFU g^−1^ lower bacterial load as compared to the untreated mice, while colistin showed a 4.8 log CFU g^−1^ load lower bacterial load as compared to the untreated mice at the same dose (Fig. 6C). The use of colistin is however limited due to its toxicity and bacteria easily develop resistance to it. Therefore, VanQAmC_10_ presents a promising alternative for both Gram-positive and Gram-negative bacteria.

## Discussion

Vancomycin served as a life-saving drug against MDR Gram-positive bacterial infections for over thirty years until the report of resistance. This makes it an attractive drug for further development against the escalating incidences of resistant bacteria. The more difficult to treat Gram-negative bacteria are inherently resistant to vancomycin. We have shown that the conjugation of cationic lipophilic moieties to vancomycin results in broad-spectrum activity against both Gram-negative bacteria and Gram-positive bacteria. The cationic lipophilic moiety incorporates interaction with the negatively charged bacterial membrane, therefore, perturbing membrane integrity. Through comparison of alkyl and aryl-substituted derivatives, VanQAmC_10_ and VanQbiph respectively, we demonstrate that the alkyl substitutions exhibit better membrane activity as well as bactericidal activity against both metabolically active and inactive bacteria (Fig. 1). Vancomycin, on the other hand, is ineffective against the metabolically inactive bacterial cells. The lead compound, VanQAmC_10_ shows remarkable improvement in antibacterial efficacy in a mouse thigh infection model as compared to vancomycin against MRSA (Fig. 6). It was also effective against burn wound infections in mice caused by *A. baumannii*.

Scientific interest toward membrane-active vancomycin derivatives has been growing due to their ability to overcome both inherited and non-inherited resistance.^3^ Therefore, a holistic understanding of how membrane-active vancomycin derivatives affect the bacteria would be imperative for the further development of new agents. In a step toward this, we used a variety of biological assays to provide new insights into the mechanism of action of VanQAmC_10_. It acts through mechanisms in addition to cell wall synthesis inhibition by binding to the d-Ala–d-Ala terminus which is known for the parent drug, vancomycin. To rule out the secondary effects as a result of cell death, mechanisms of action were studied at sub-inhibitory concentrations. Systematic investigations in *B. subtilis* revealed that it acts by simultaneously, (i) inhibiting cell wall biosynthesis, (ii) permeabilizing cells through non-specific interactions with membrane, and (iii) dissipation of membrane-potential leading to delocalization of the cell division protein, MinD (Fig. 4). The dissipation of membrane potential by VanQAmC_10_ possibly results in malfunctioning of the bacterial cell division machinery.^42^ To comprehend how VanQAmC_10_ affects Gram-negative bacteria, studies were conducted on the model Gram-negative bacterium, *E. coli*. Treatment with VanQAmC_10_ results in the lysis of the cells and therefore cell death. Treatment of the various strains of *E. coli* (wild-type, GFP-FtsI, GFP-FtsZ, and Δ*amiAC*) at subinhibitory concentrations resulted in cells of variable sizes and distorted morphology (Fig. 5). It did not impede the formation of the Z-ring in the first stage of cell division. It delocalized the FtsI protein required for the synthesis of the cell wall during septum formation in the second stage of cell division. Mutants lacking AmiA and AmiC enzymes were more sensitive to VanQAmC_10_ which is consistent with reports that these mutants have increased susceptibility to surfactants. These confirm that VanQAmC_10_ inhibits cell division at sub-inhibitory concentrations through mechanisms different from the FtsZ inhibitors and the β-lactam, cephalexin. Our findings suggest that the membrane perturbing properties of VanQAmC_10_ affect the localization of proteins involved in cell division, leading to severe cell wall and membrane defects. This possibly ultimately leads to a breach in the cell membrane and cell death.

Overall, here we demonstrate a new vancomycin derivative, VanQAmC_10_ which is highly effective against Gram-positive as well as Gram-negative bacteria both *in vitro* and *in vivo*. The multiple mechanisms of action contribute to the negligible resistance induction and superior antibacterial properties of VanQAmC_10_ as compared to the parent drug. It represents a new class of multi-target, multi-effect glycopeptide antibiotics. The findings augment a new dimension to the understanding of the mechanism of such membrane-active glycopeptide derivatives.

## Data availability

The datasets supporting this article have been uploaded as part of the ESI.The ESI† includes materials and methods; experimental protocols for *in vitro* microbiological assays (activity, biofilms, time-kill kinetics, and mechanistic studies) and toxicity, and *in vivo* activity and toxicity; supplementary figures; details of characterization of intermediates and final compounds through ^1^H NMR, IR, and HR-MS.

## Author contributions

PS, project design, synthesis, characterisation, performing *in vitro* microbiological assays (activity and mechanistic studies) and *in vivo* infection studies, data curation, formal analysis, manuscript writing and editing; KD, synthesis and characterisation; GD, cytotoxicity testing and *in vivo* activity studies, manuscript editing; JEB, experimental design and analysis for mechanisms of action against *B. subtilis*, manuscript editing; MM and RP, experimental design and analysis for mechanisms of action against *E. coli*, manuscript editing; JH, project design, research supervision, manuscript writing and editing.

## Conflicts of interest

There are no conflicts to declare.

## Supplementary Material

SC-014-D2SC05600C-s001

SC-014-D2SC05600C-s002
