# Supplementary figures and images for: Next-generation membrane-active glycopeptide antibiotics that also inhibit bacterial cell division
Source: Chem Sci. 2023 Jan 6;14(9):2386–98. doi: 10.1039/d2sc05600c (PMC9977398; doi:10.1039/d2sc05600c)

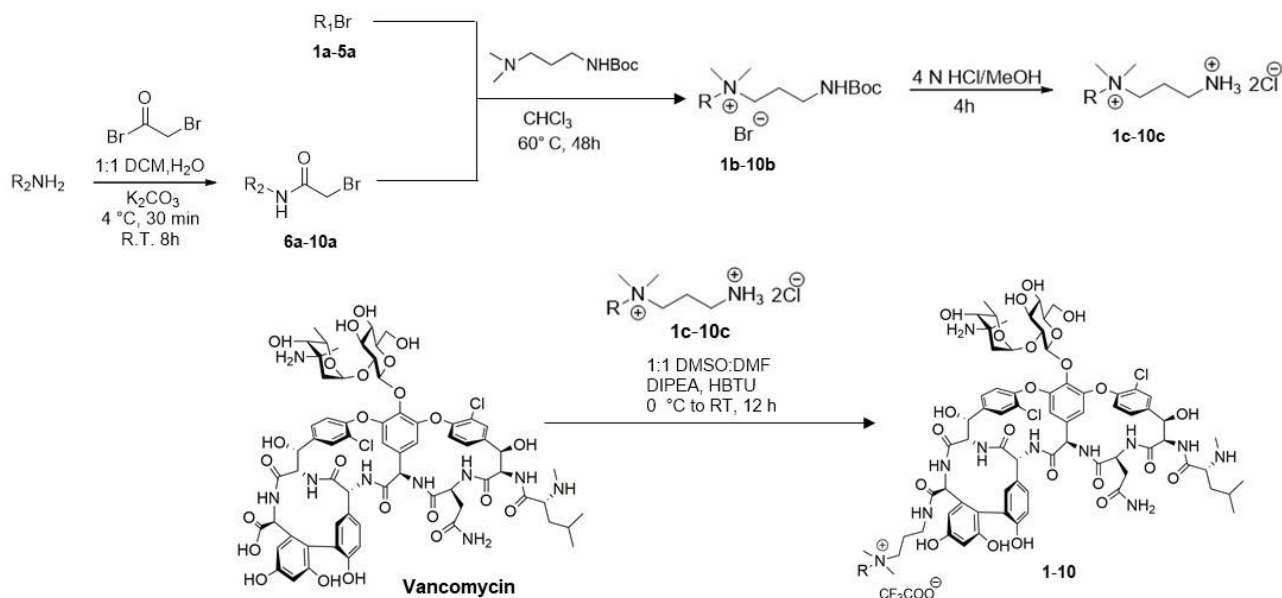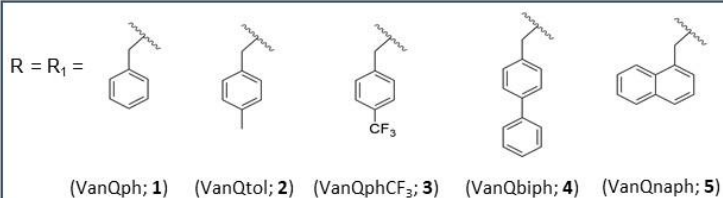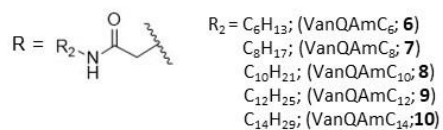

(6a)

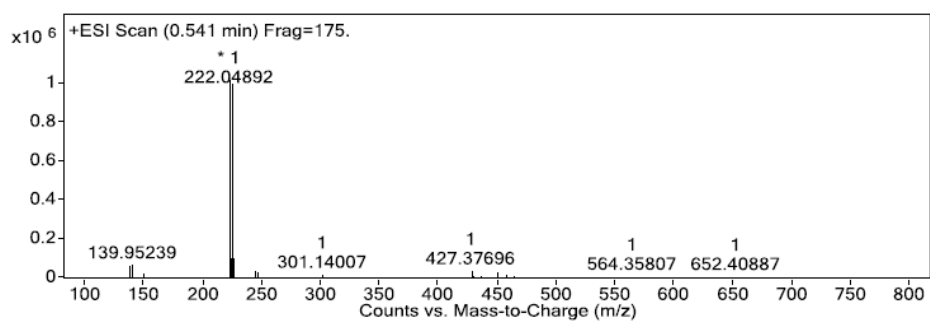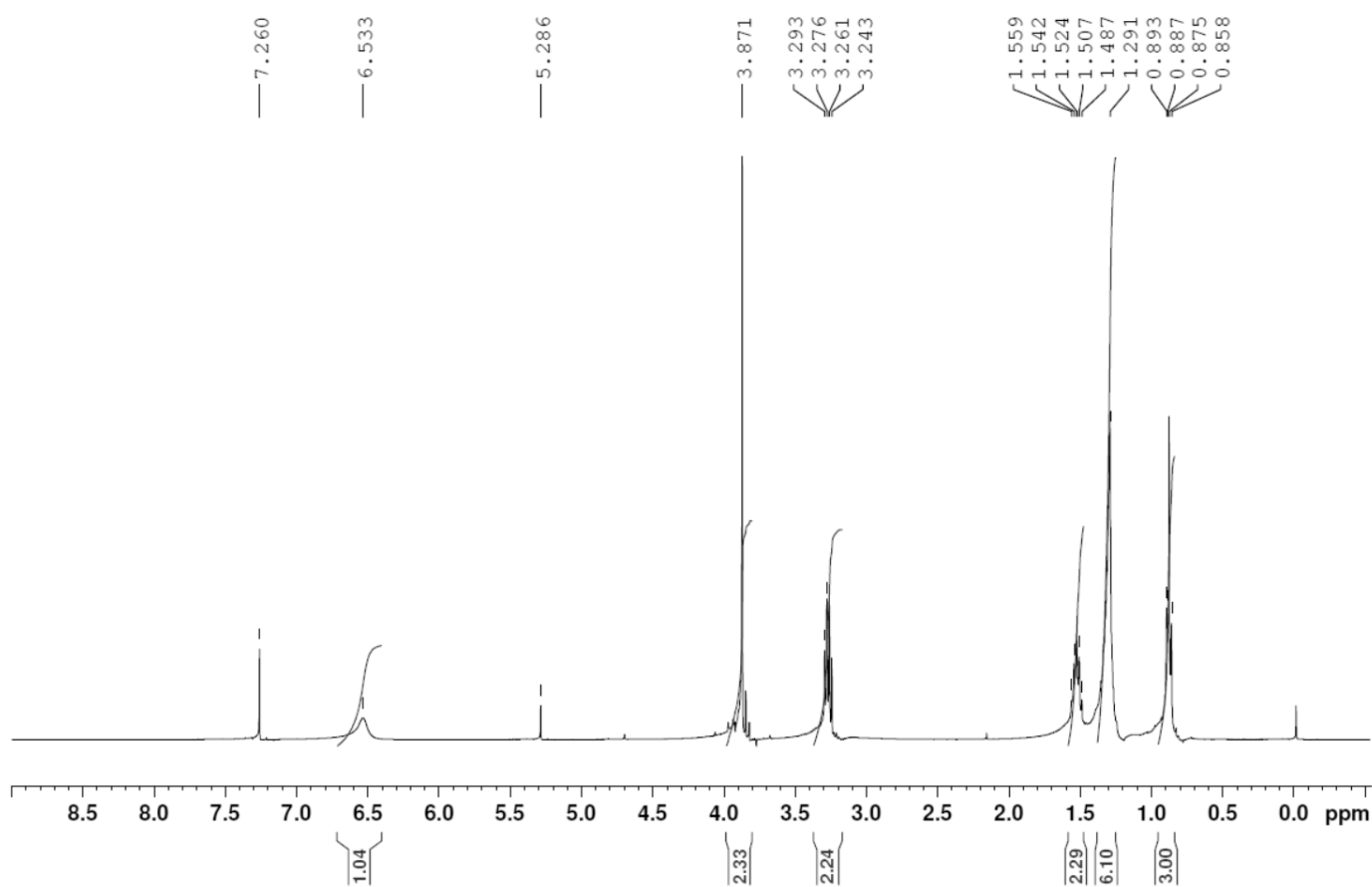

(7a)

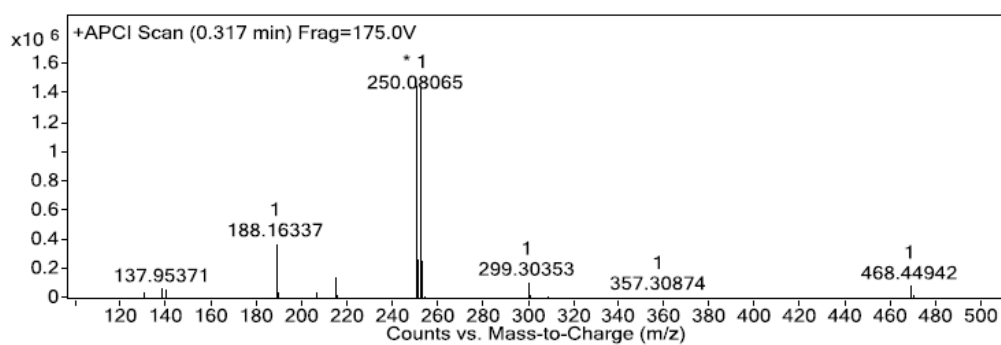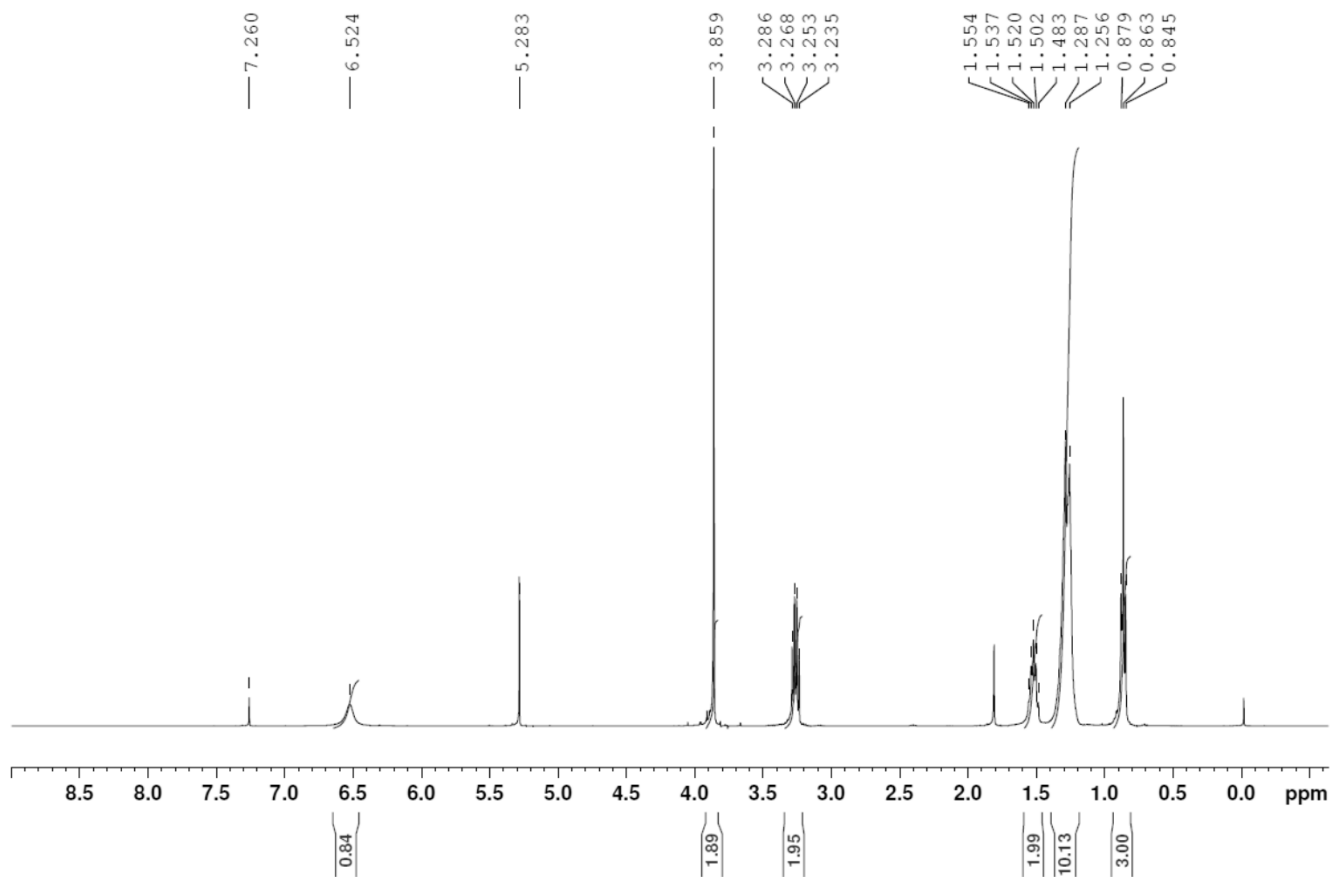

(8a)

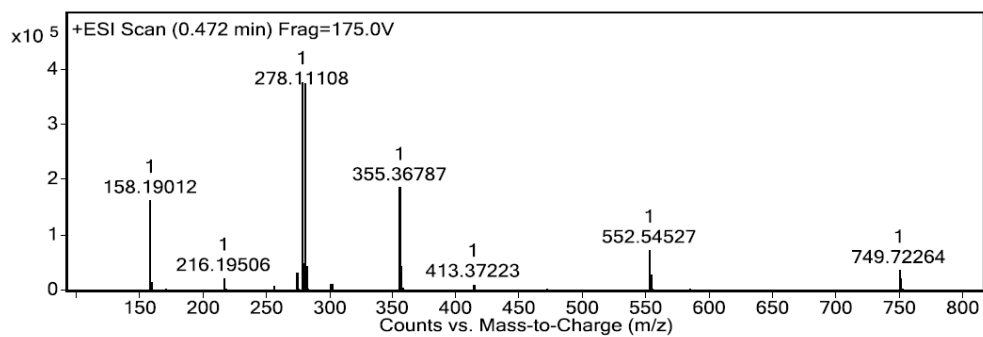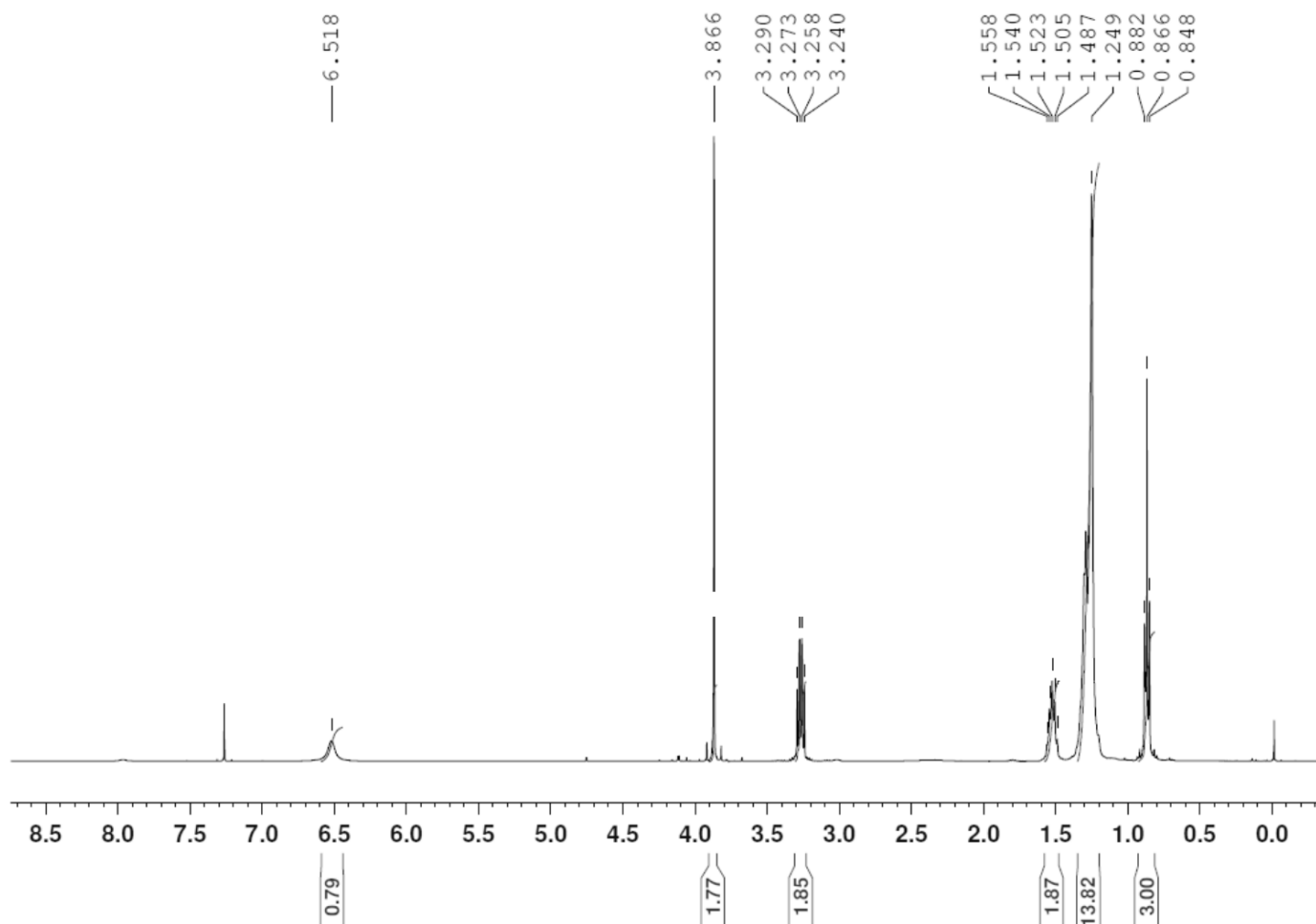

(9a)

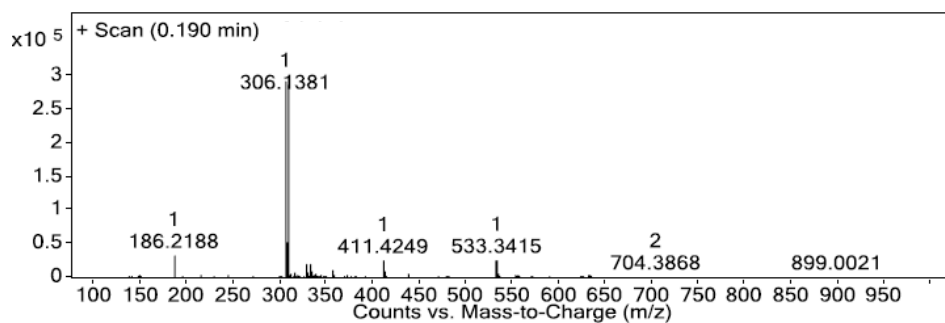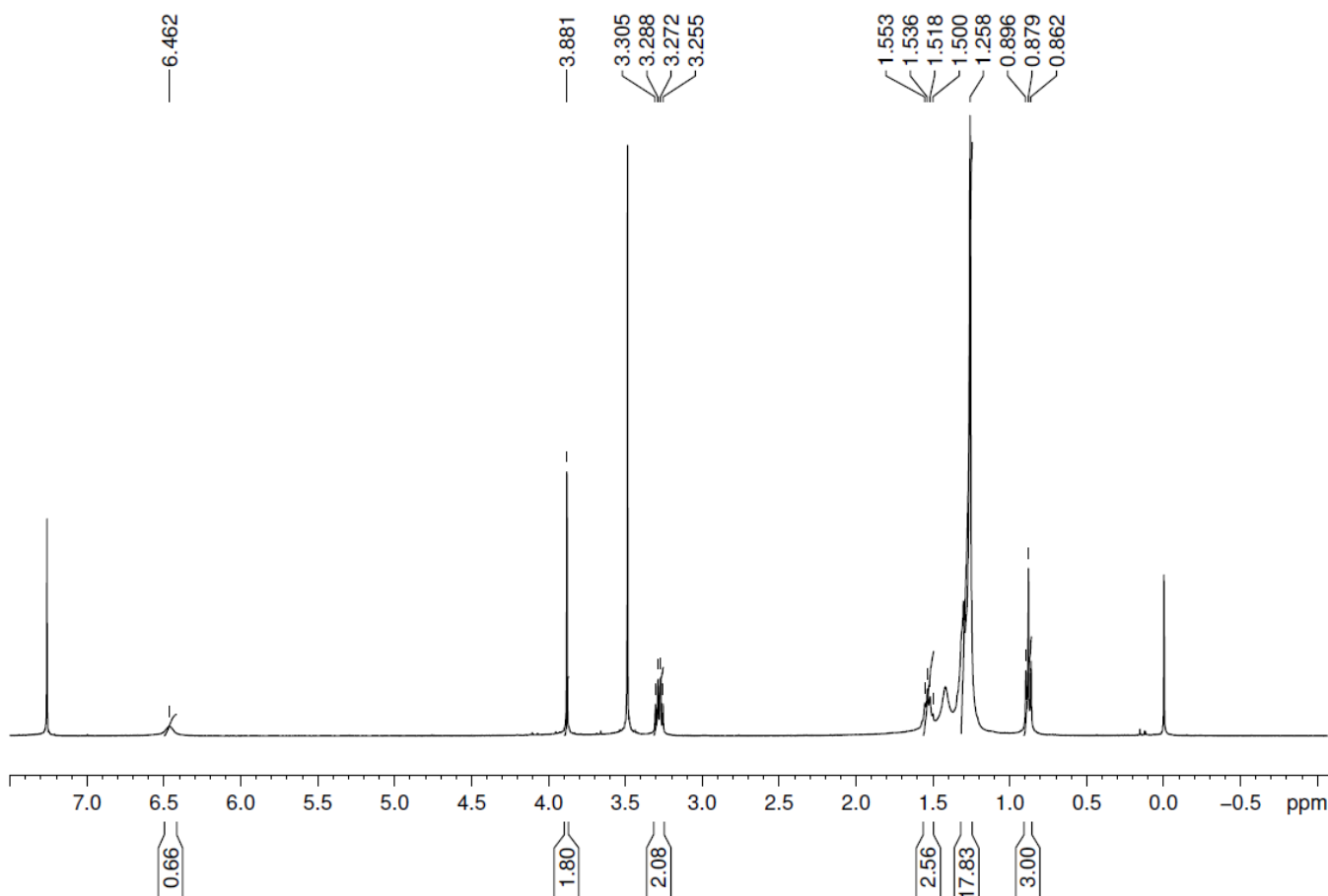

**(10a)**

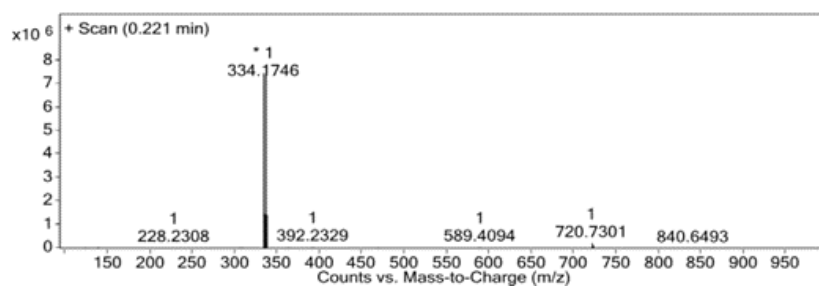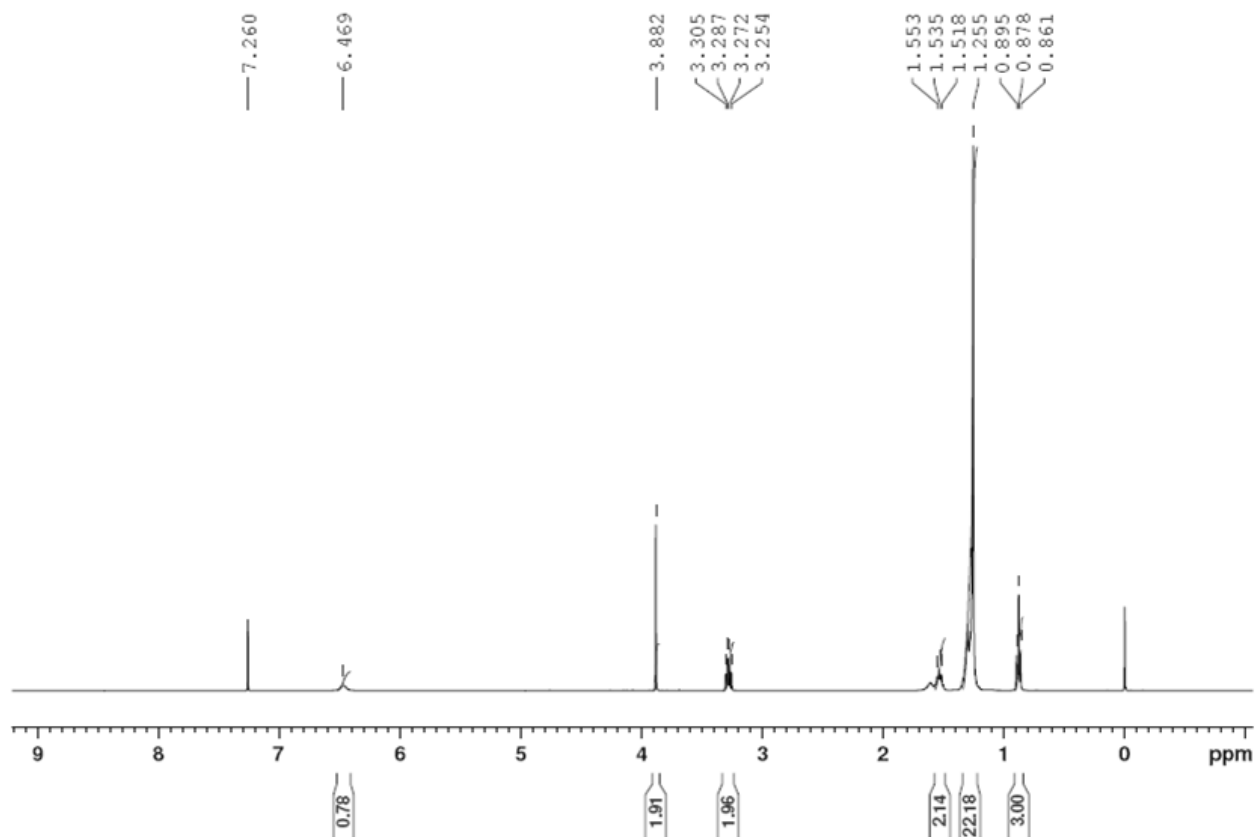

(1b)

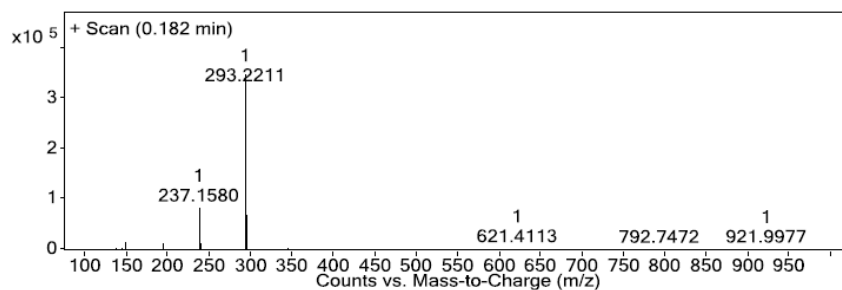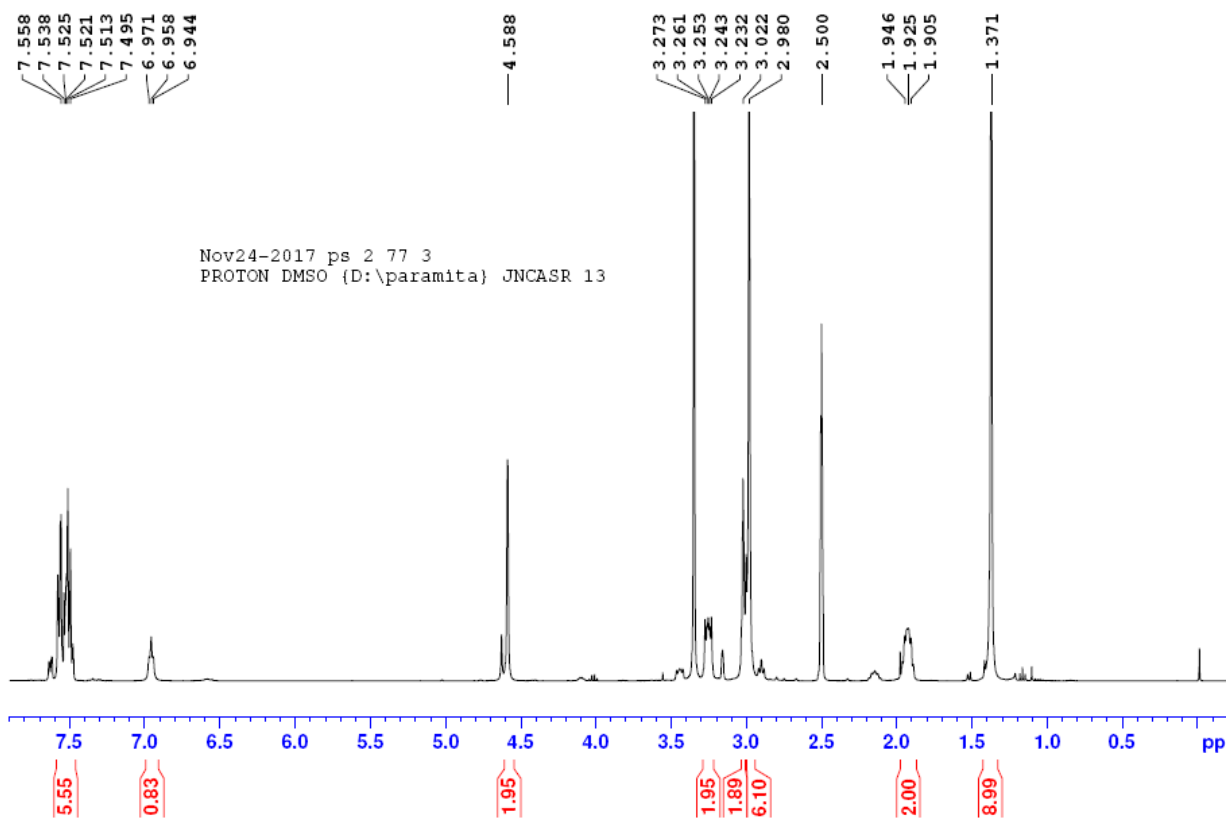

(2b)

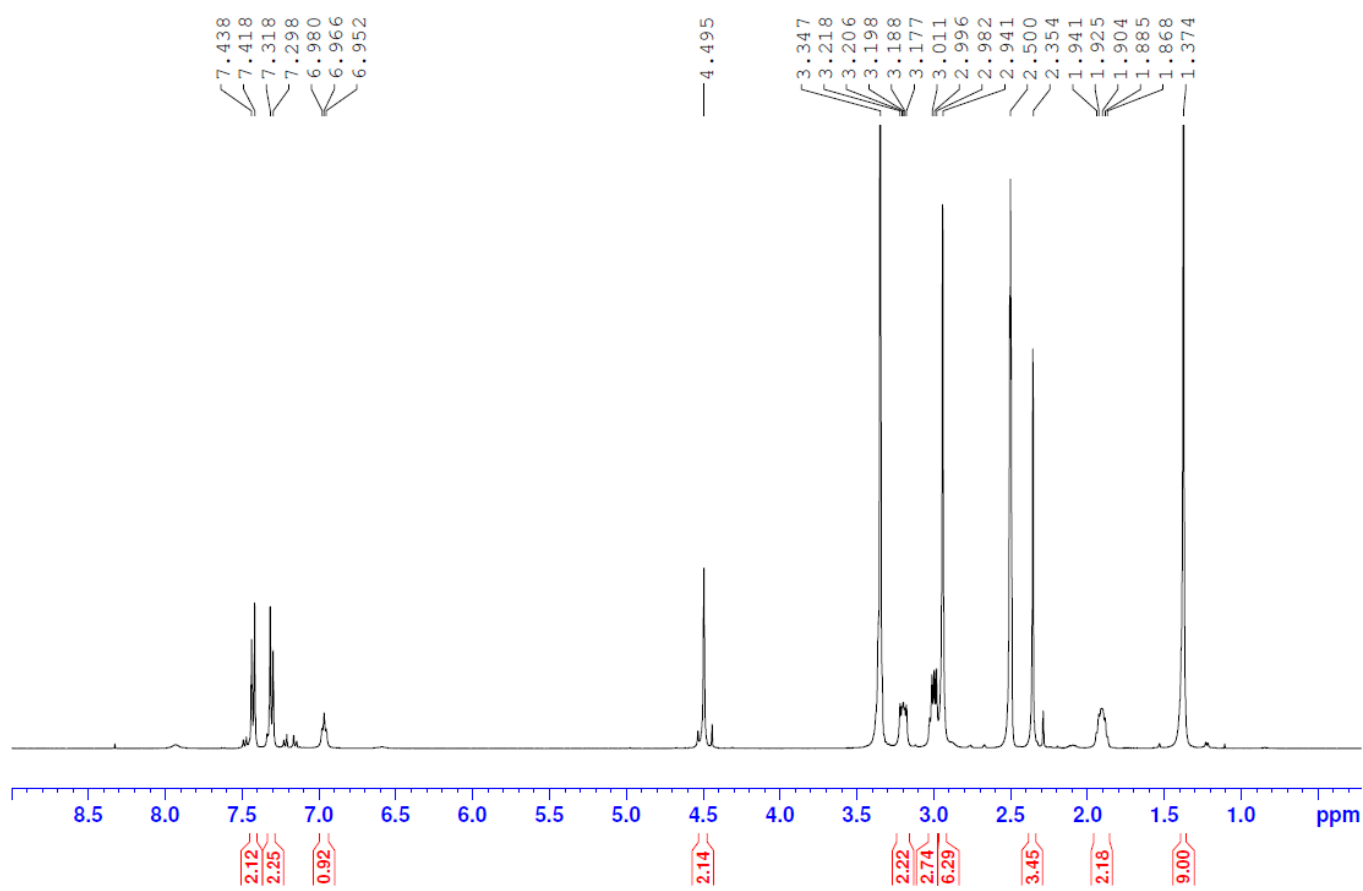

(3b)

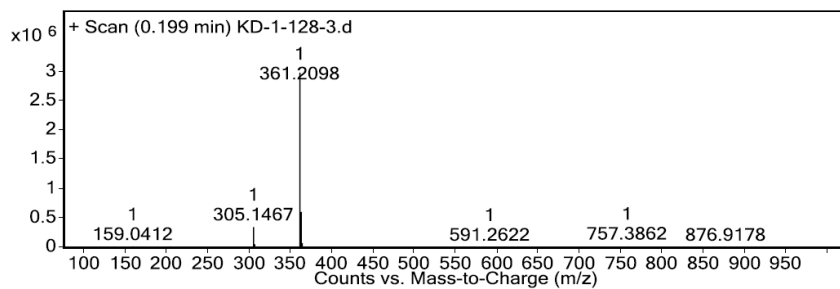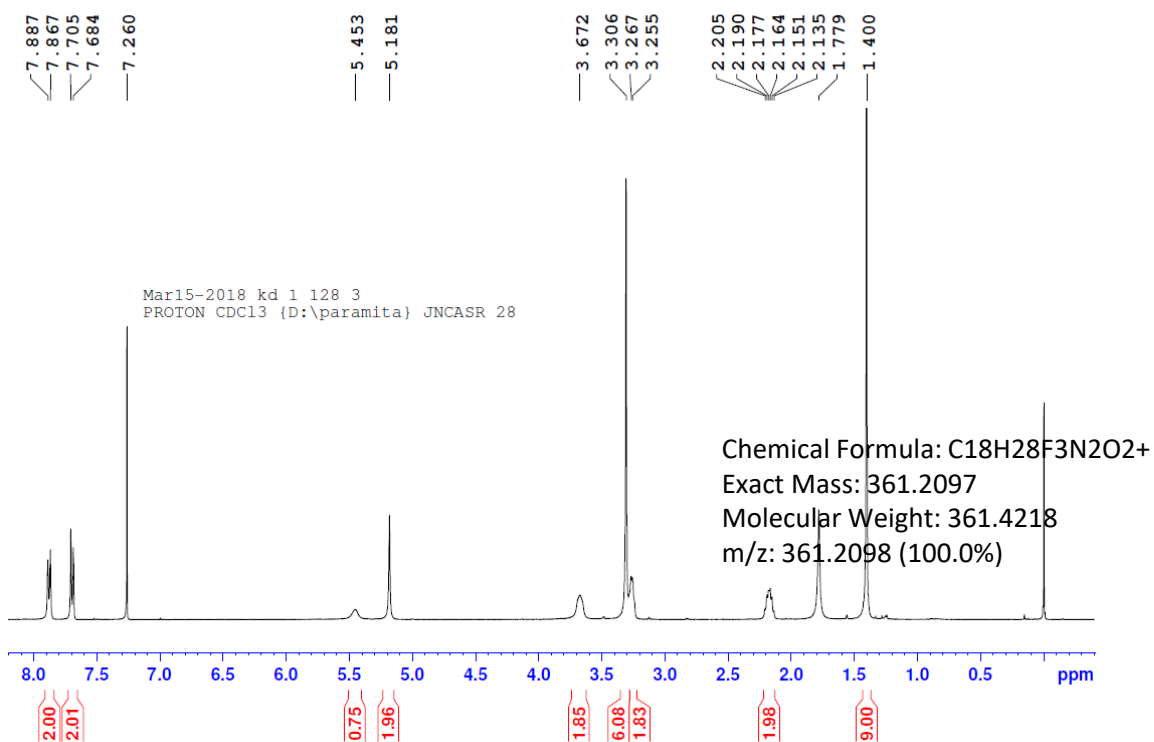

(4b)

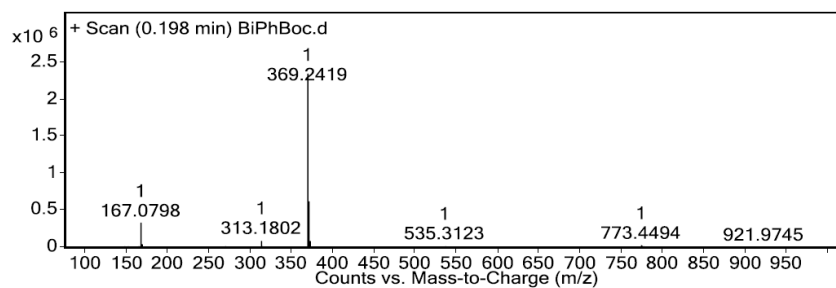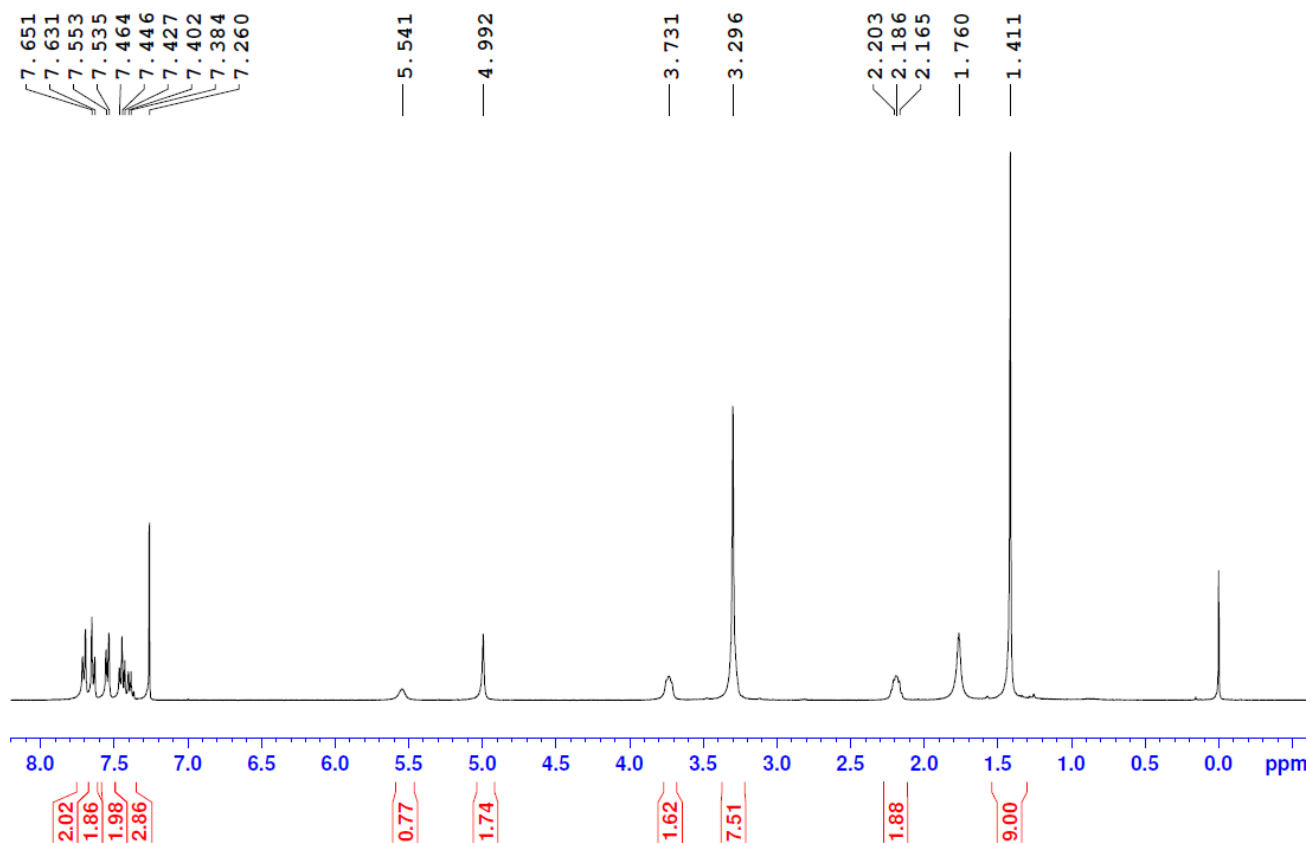

(5b)

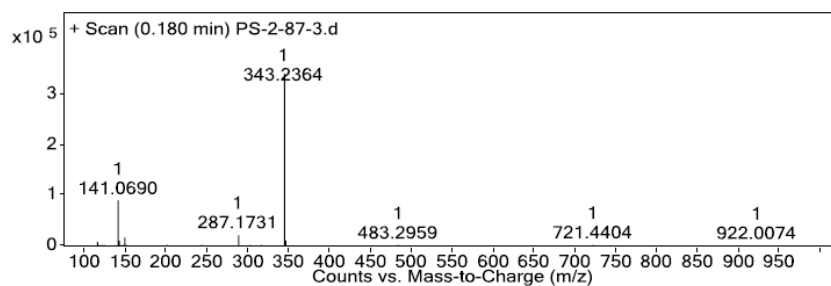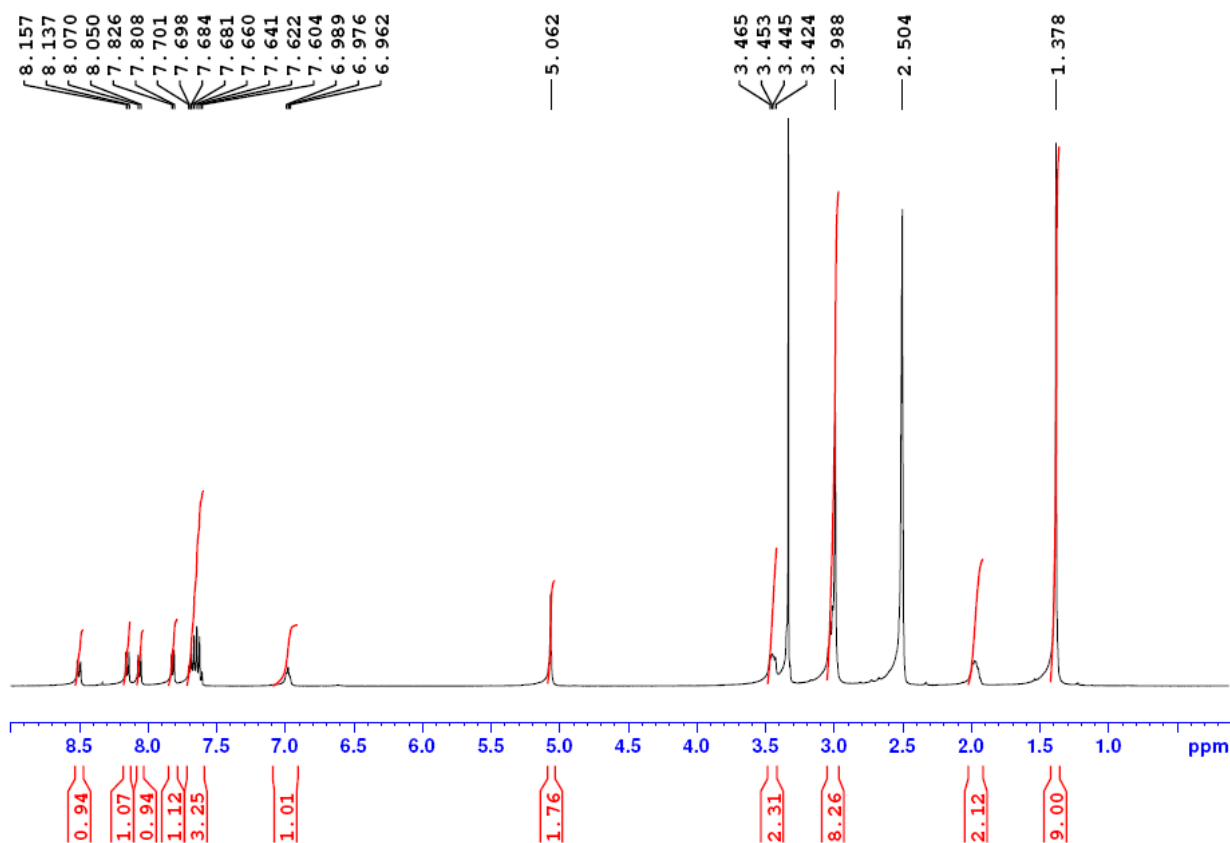

(6b)

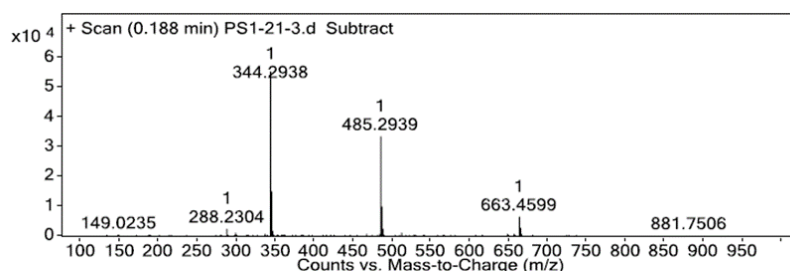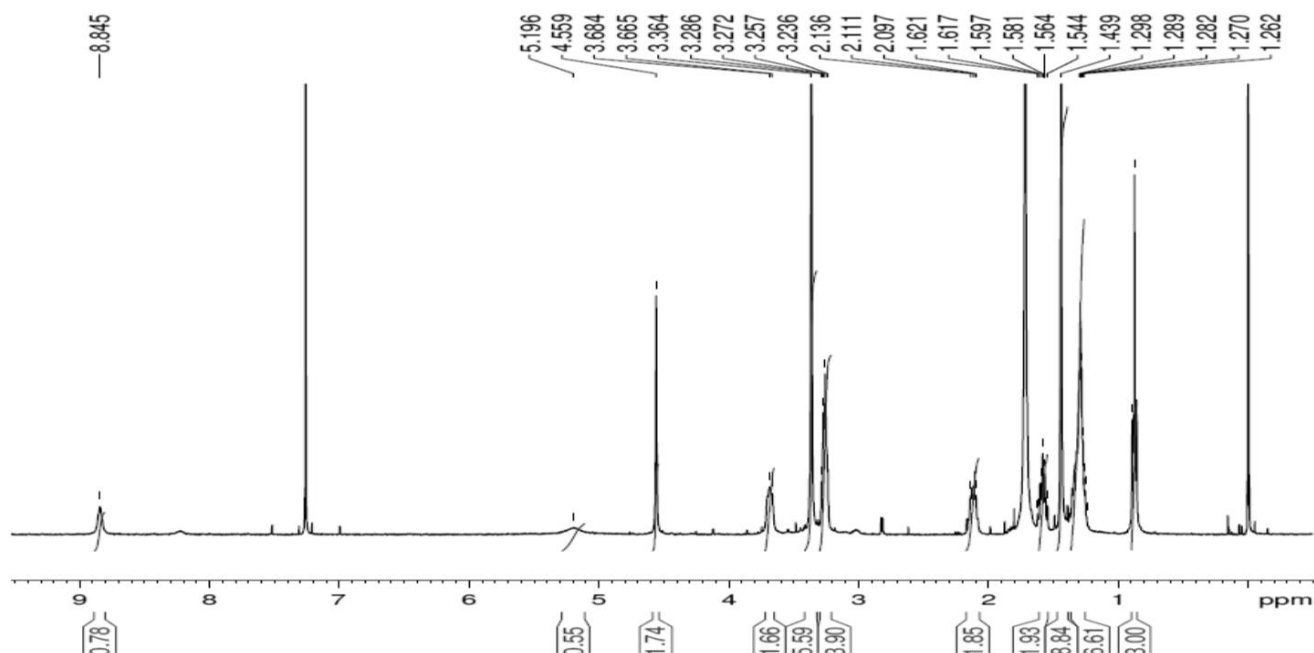

(7b)

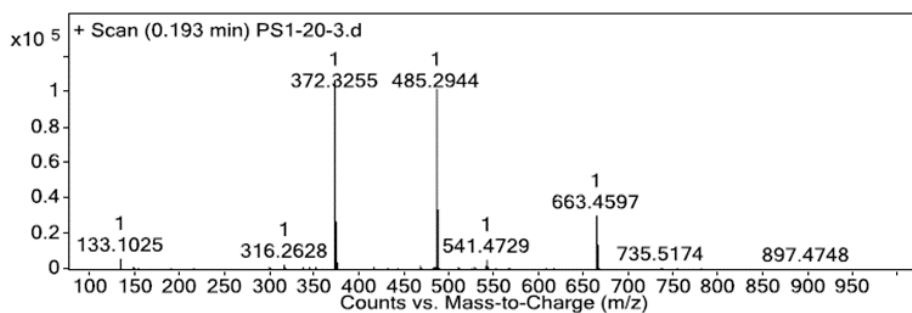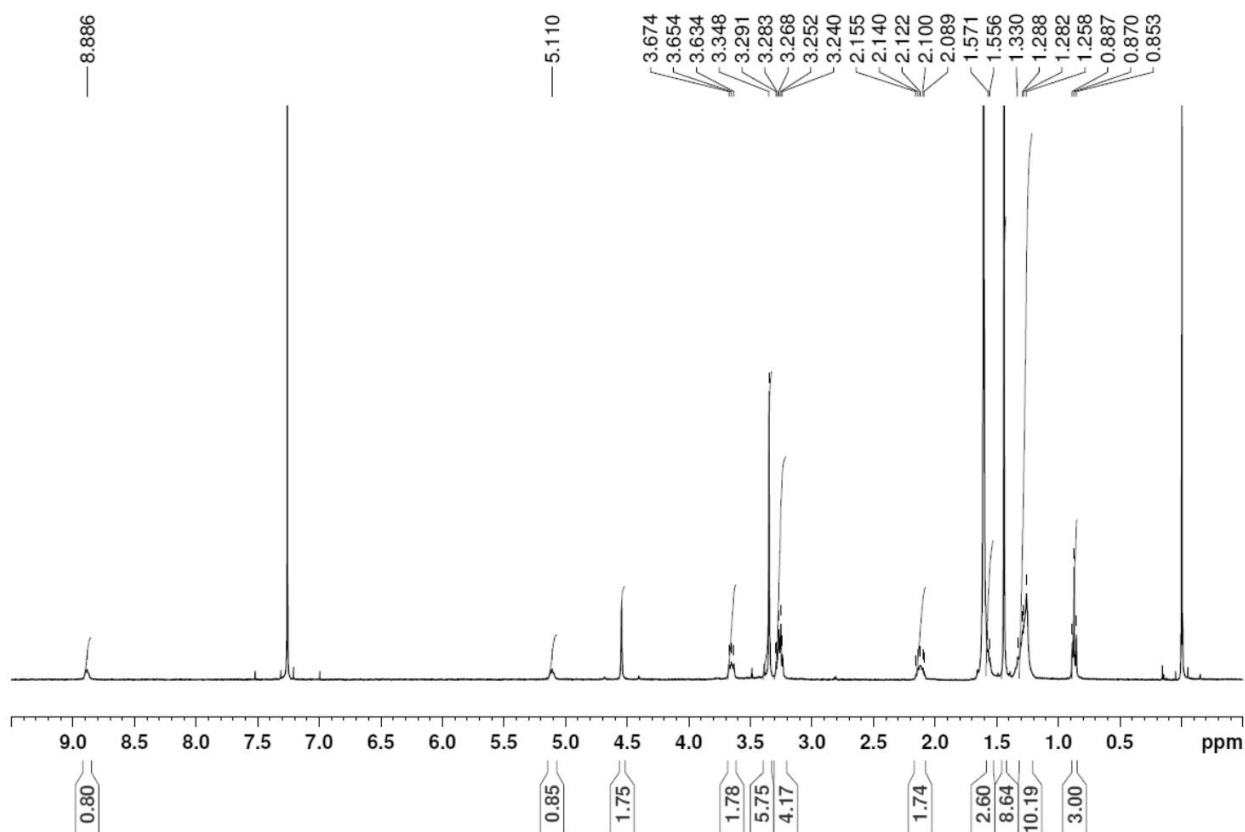

(8b)

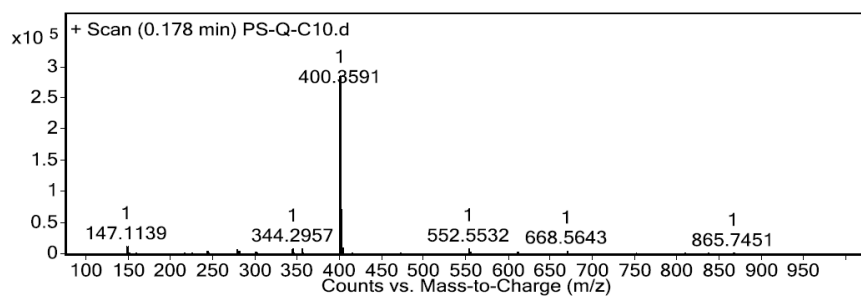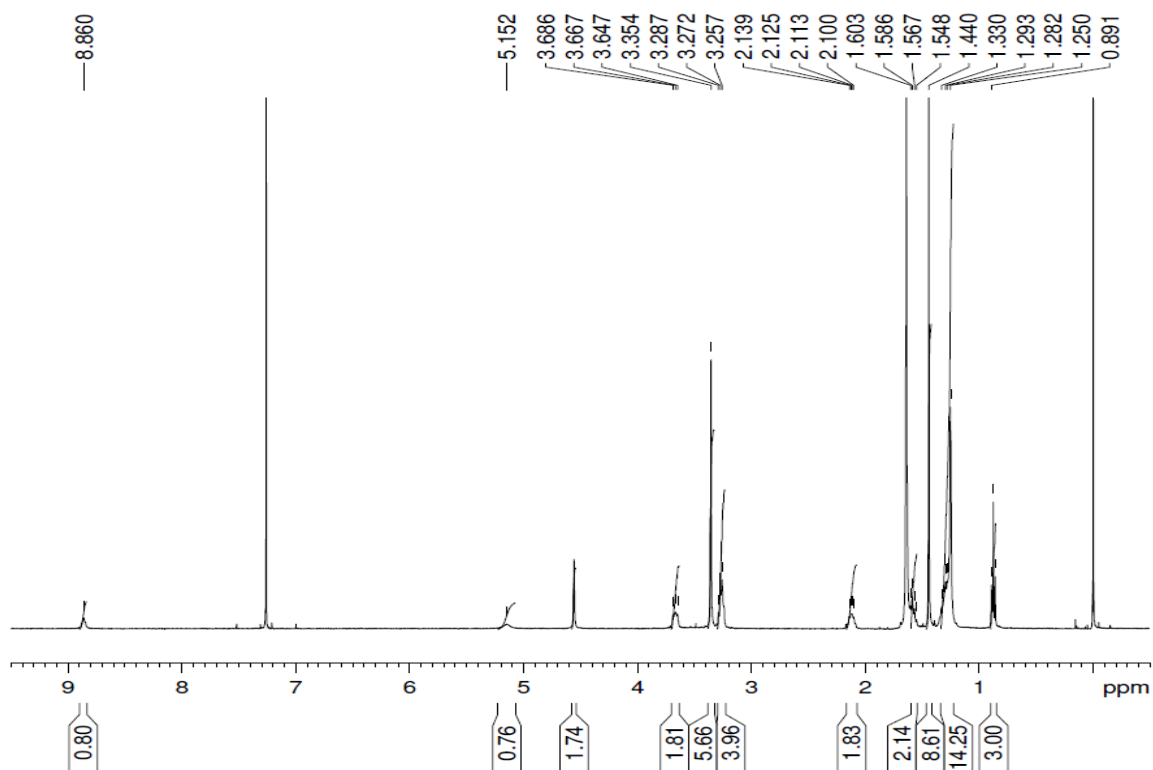

(9b)

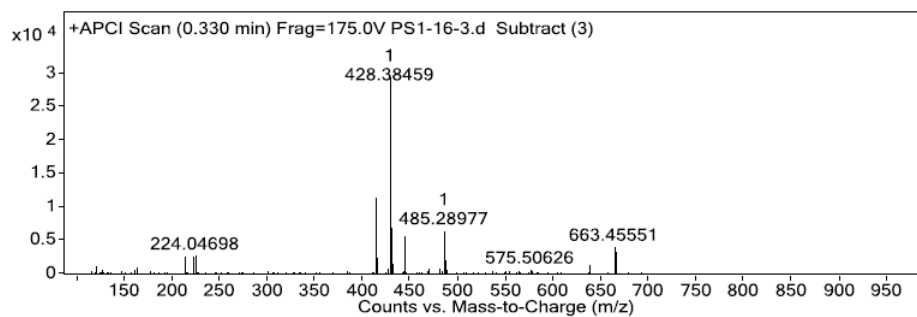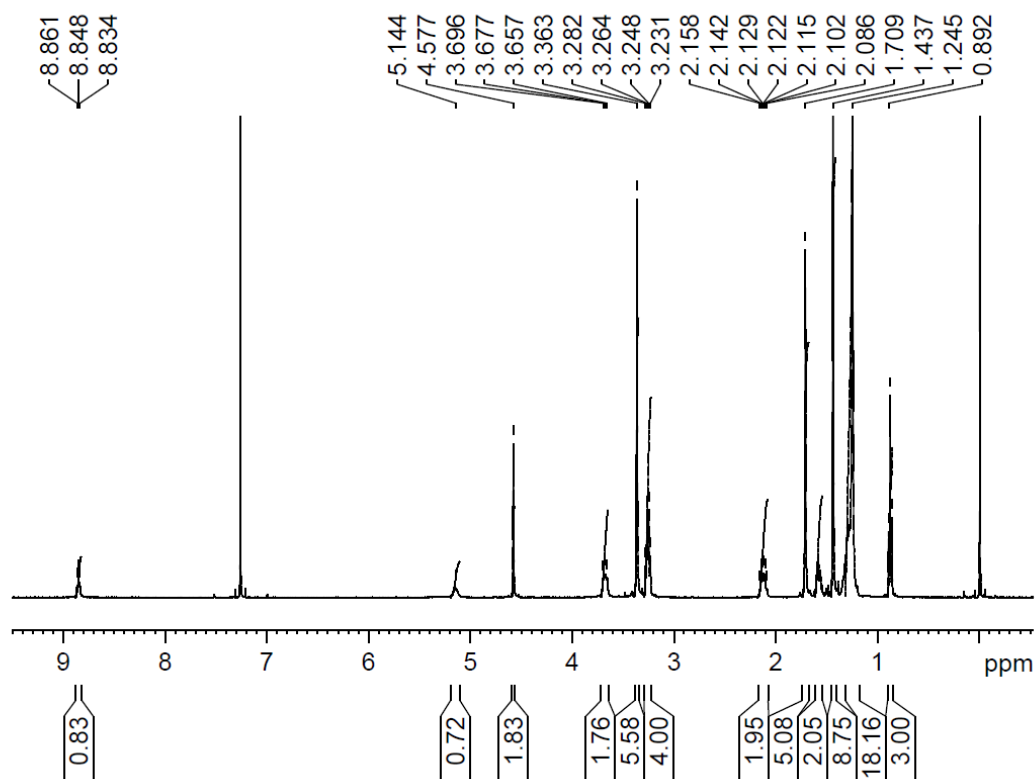

(10b)

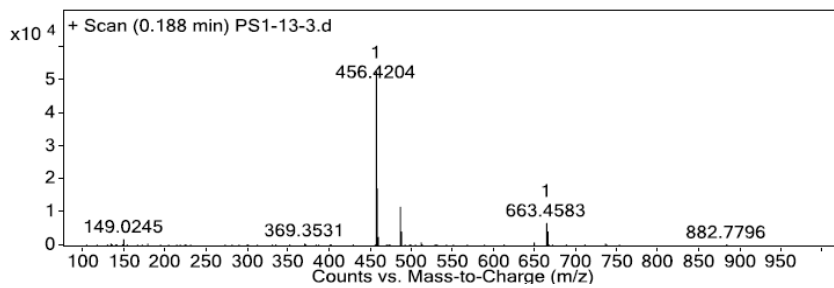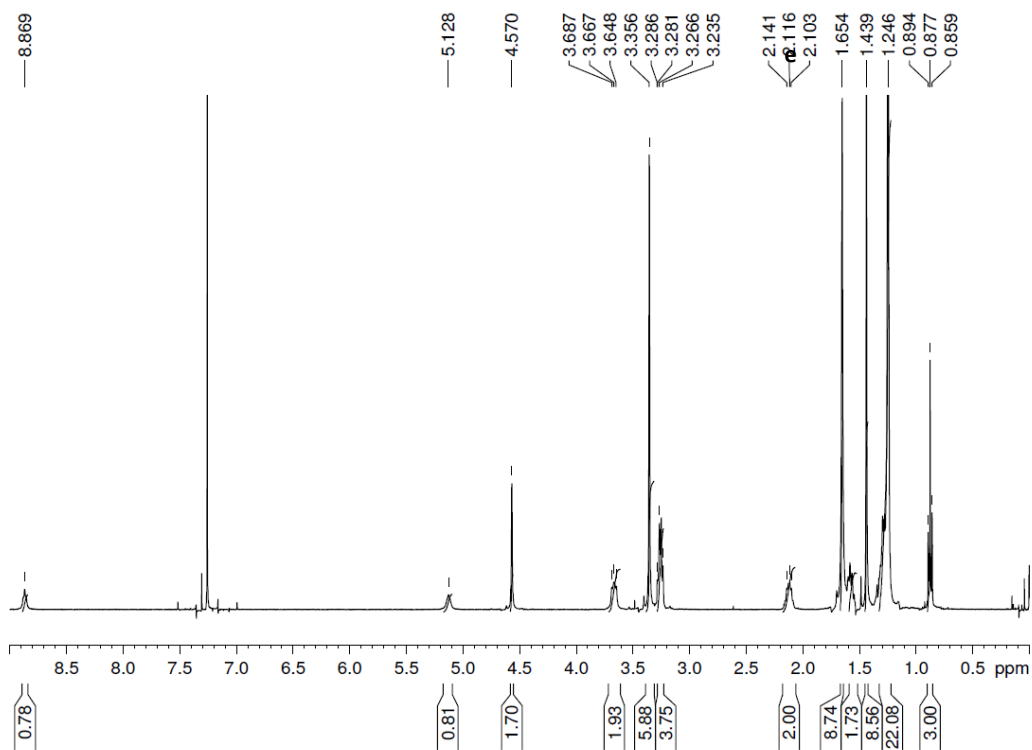

(1c)

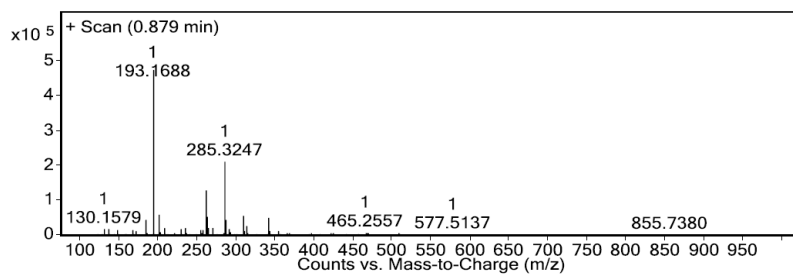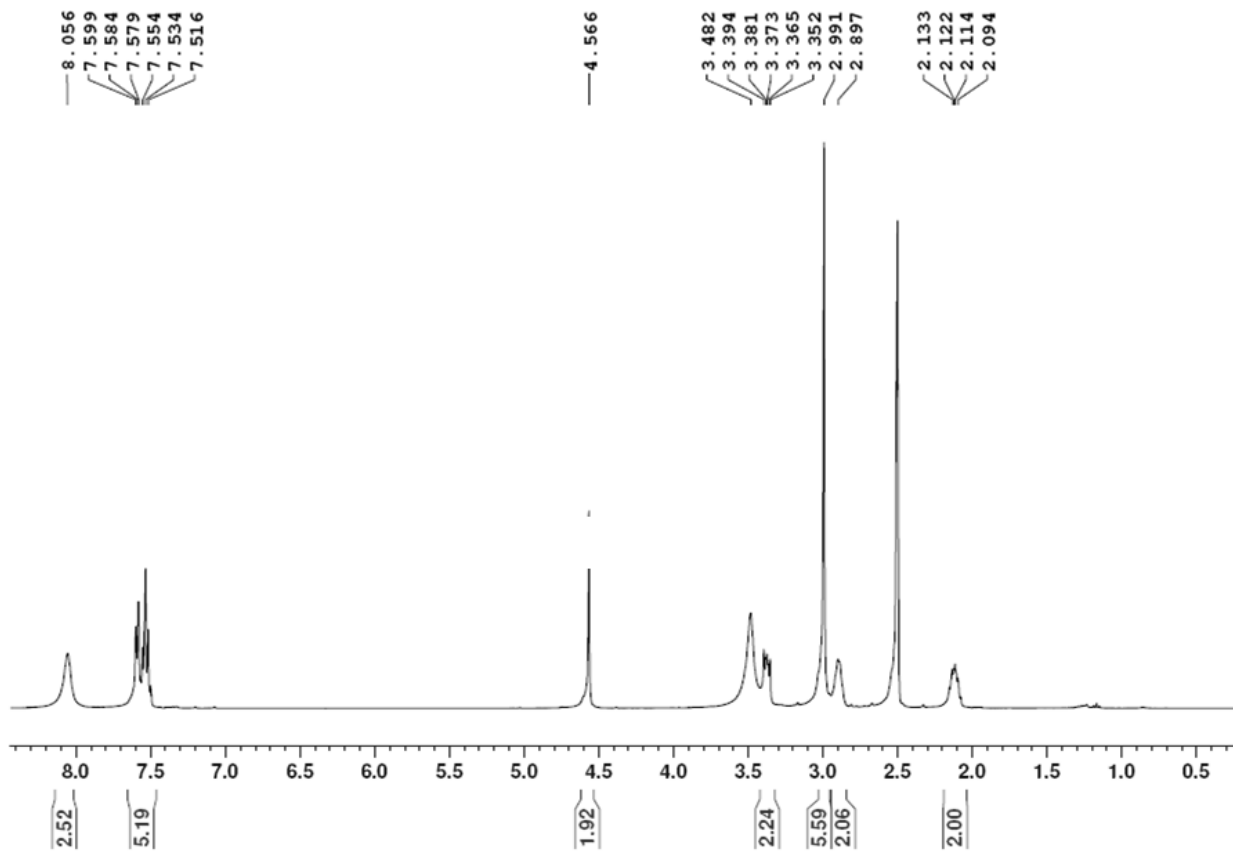

(2c)

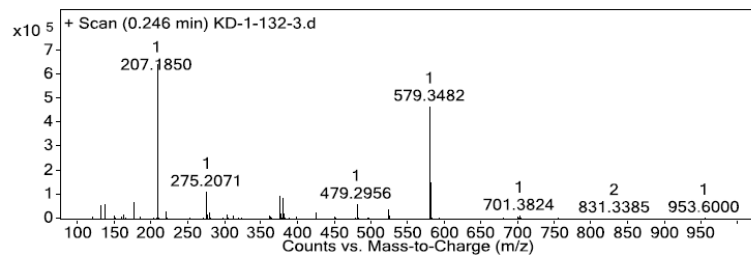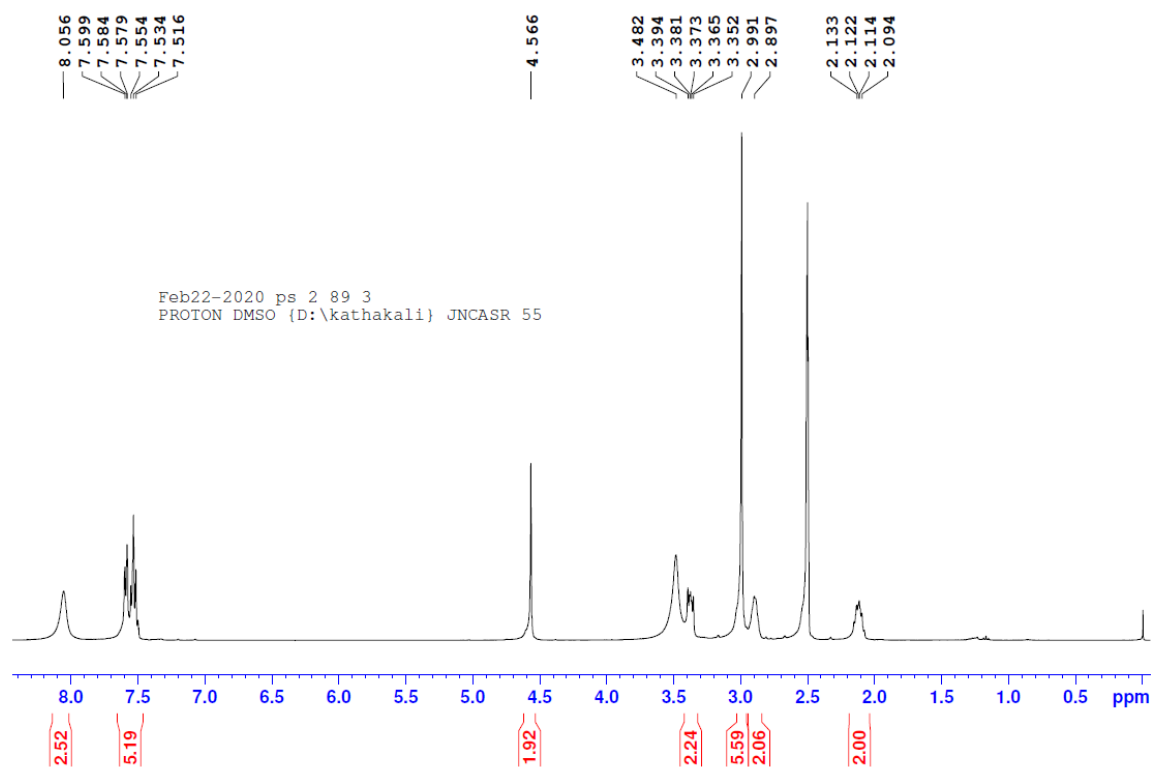

(3c)

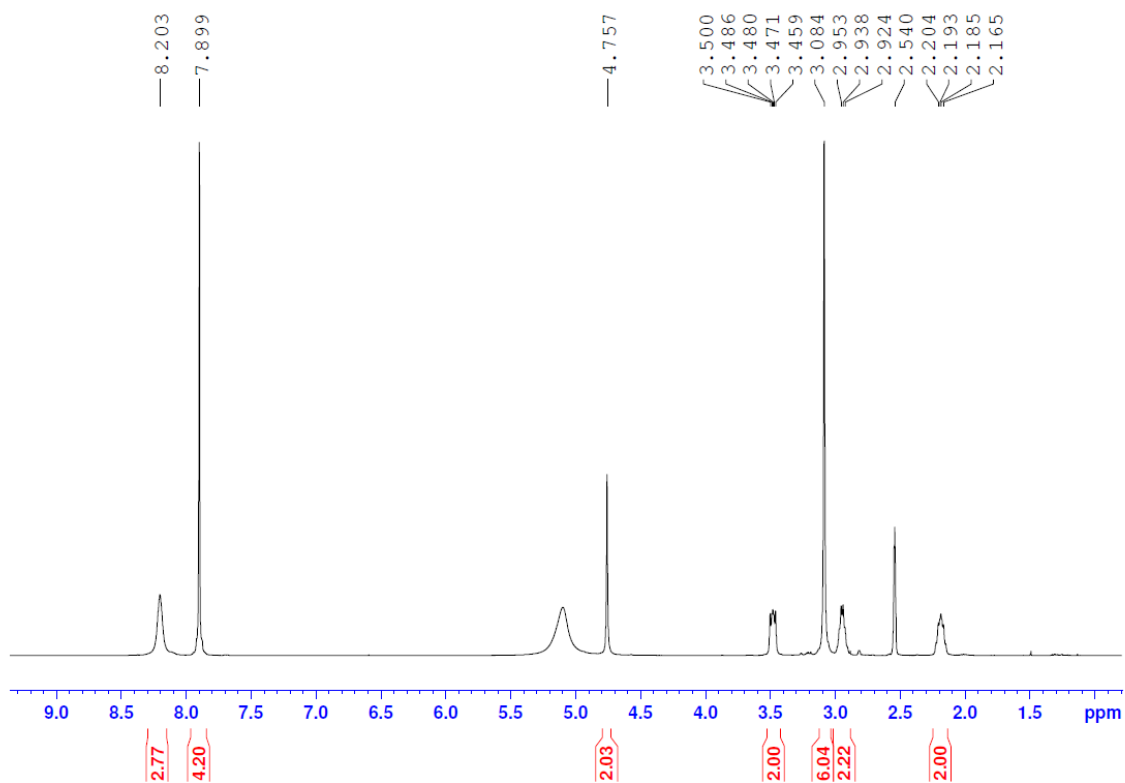

(4c)

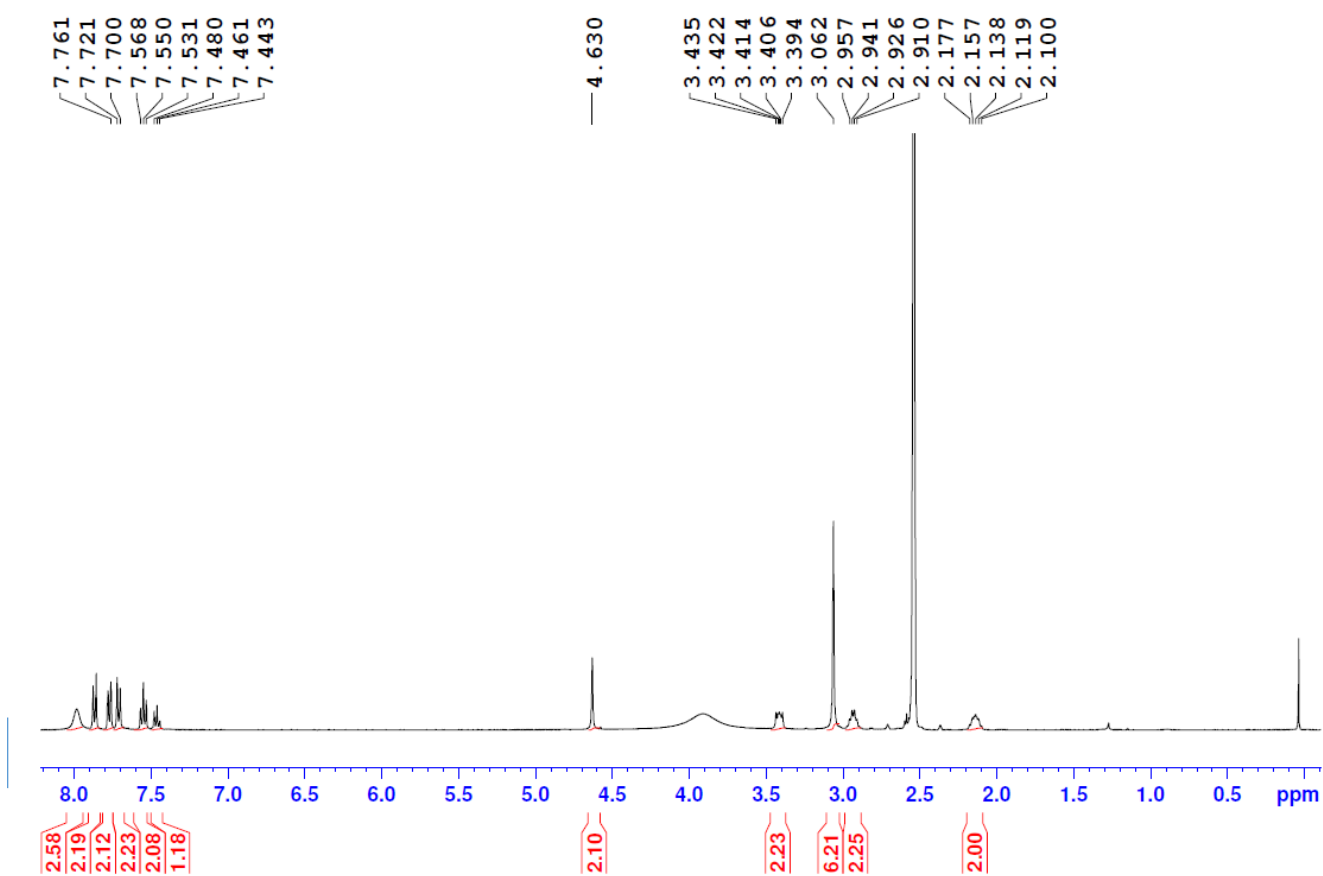

(5c)

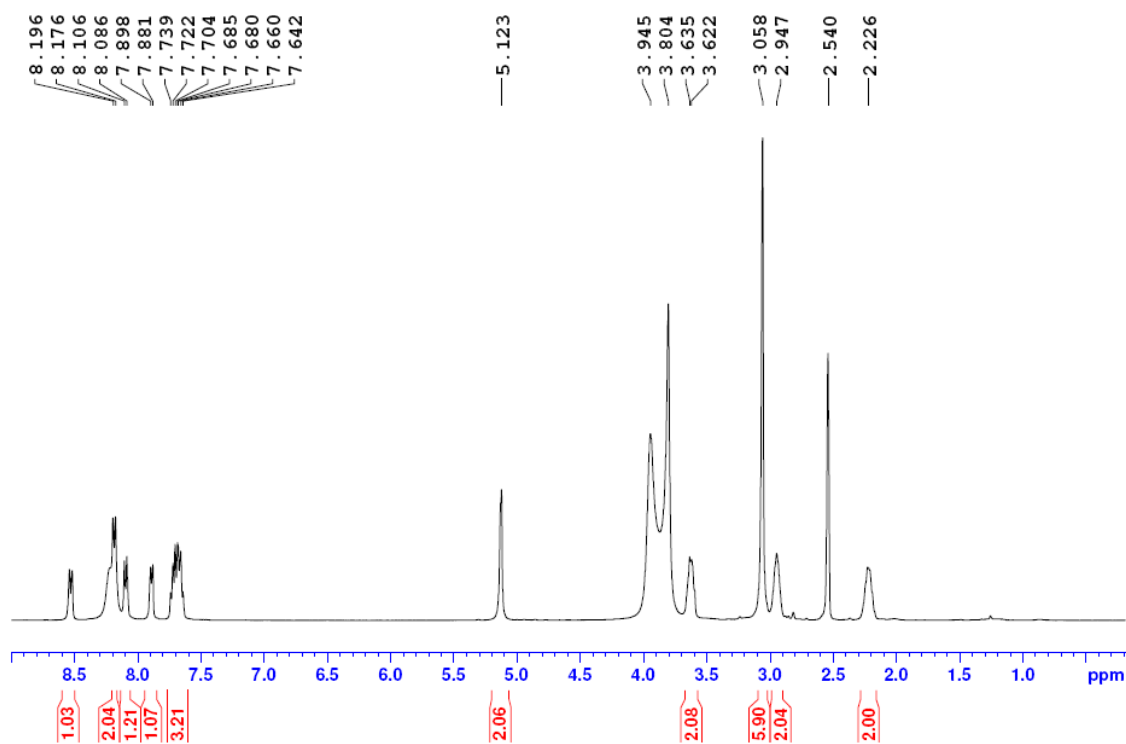

(6c)

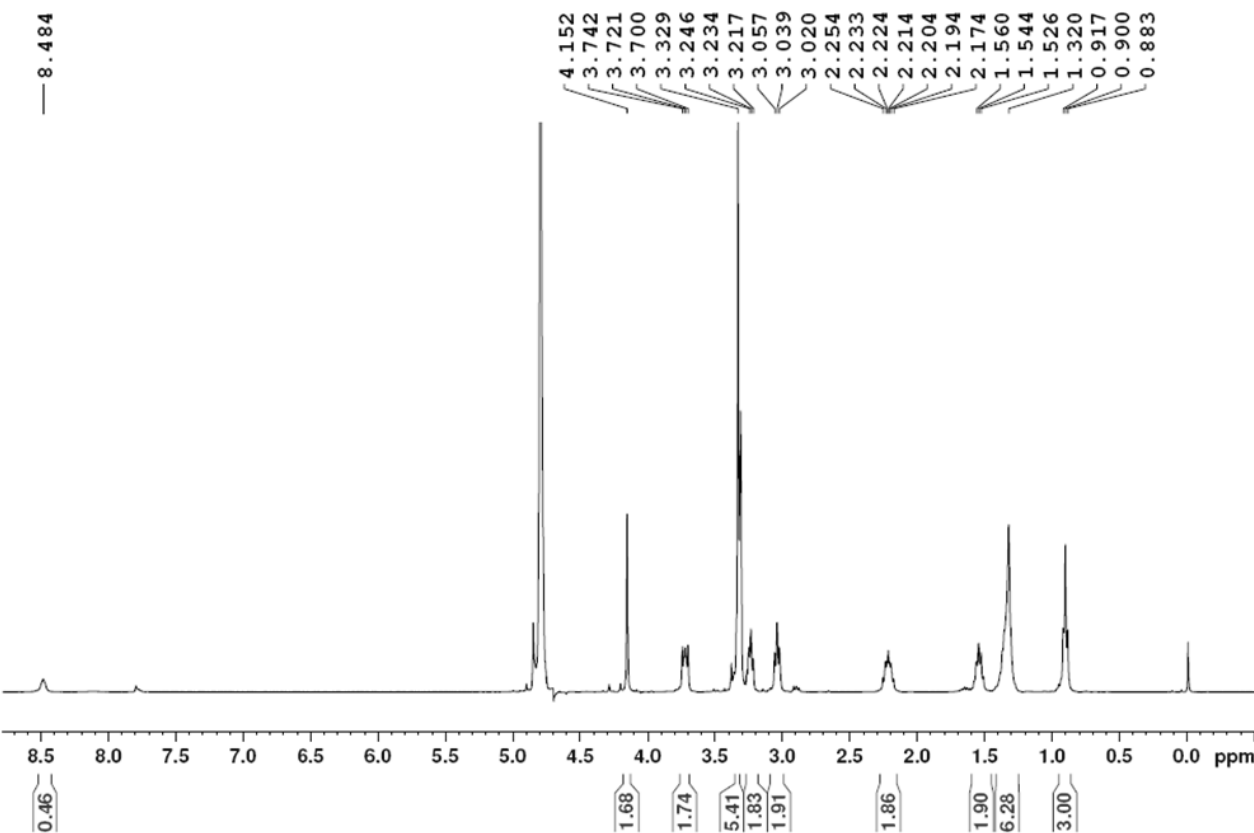

(7c)

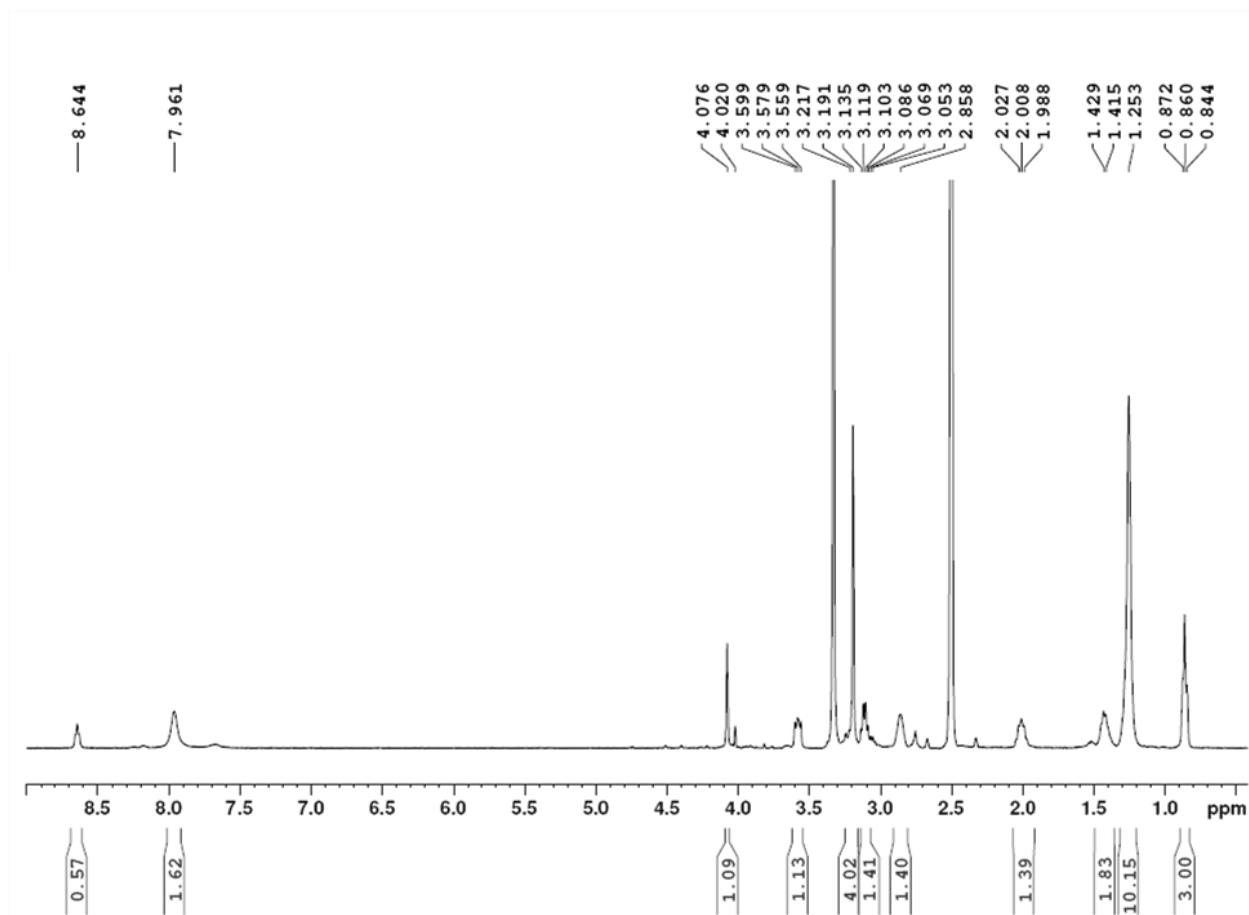

(8c)

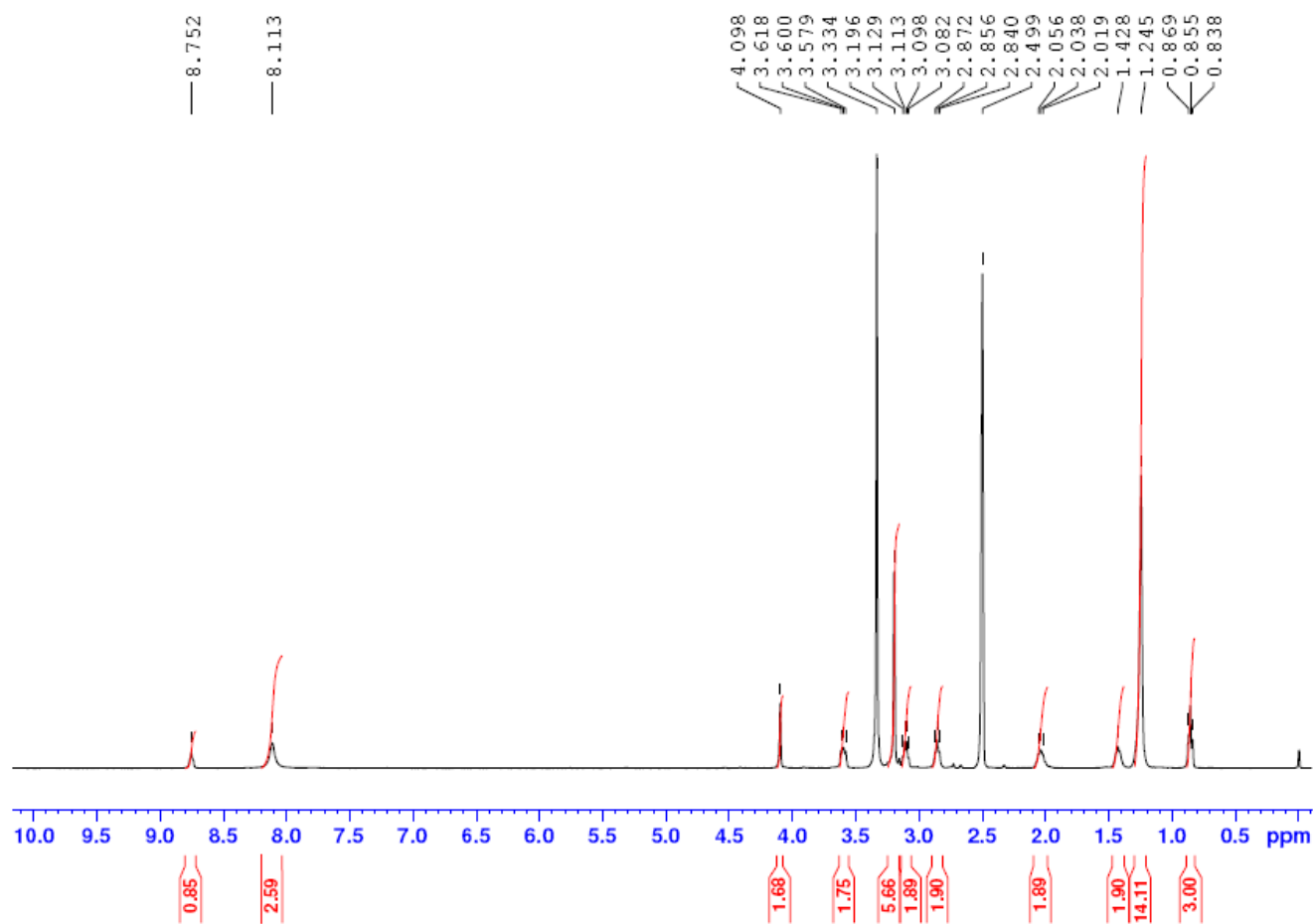

(9c)

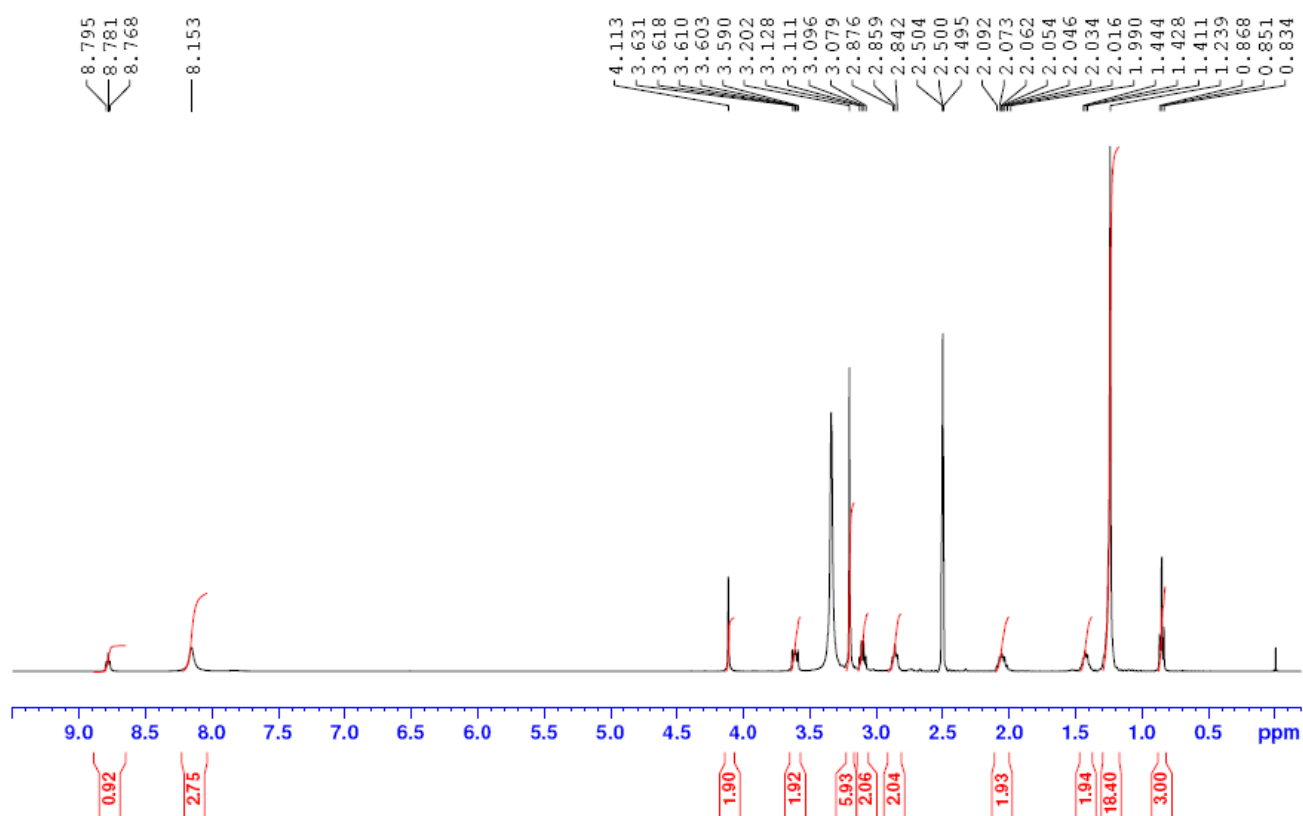

**(10c)**

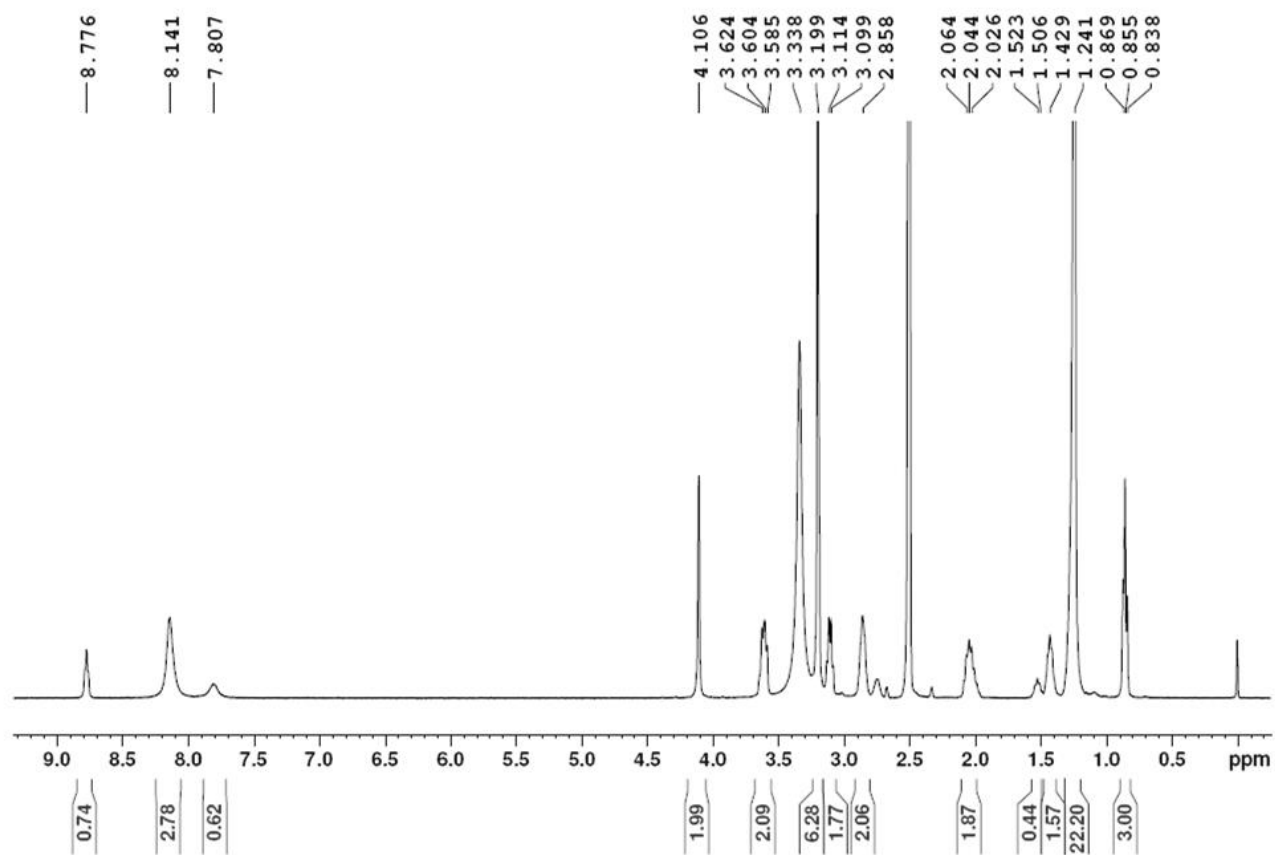

(1)

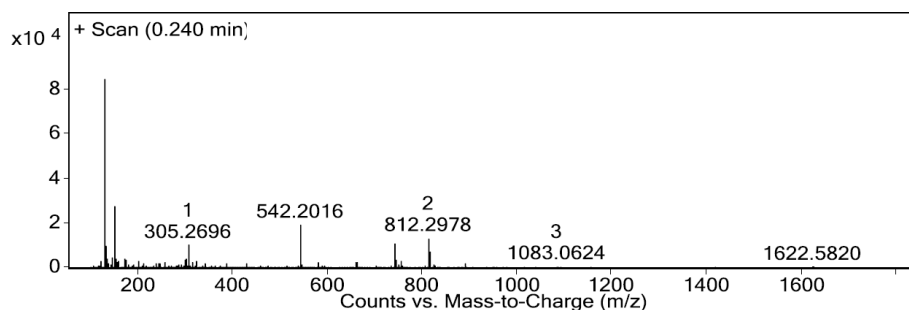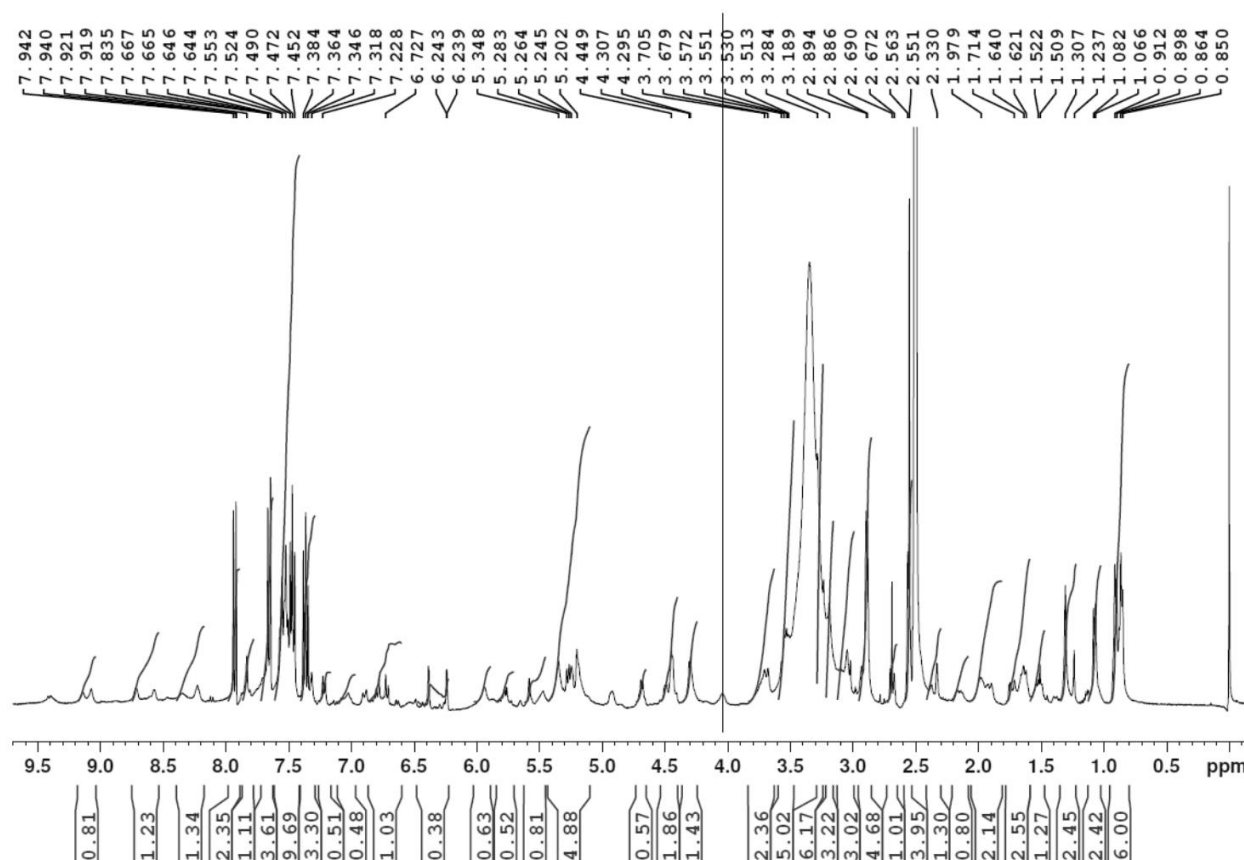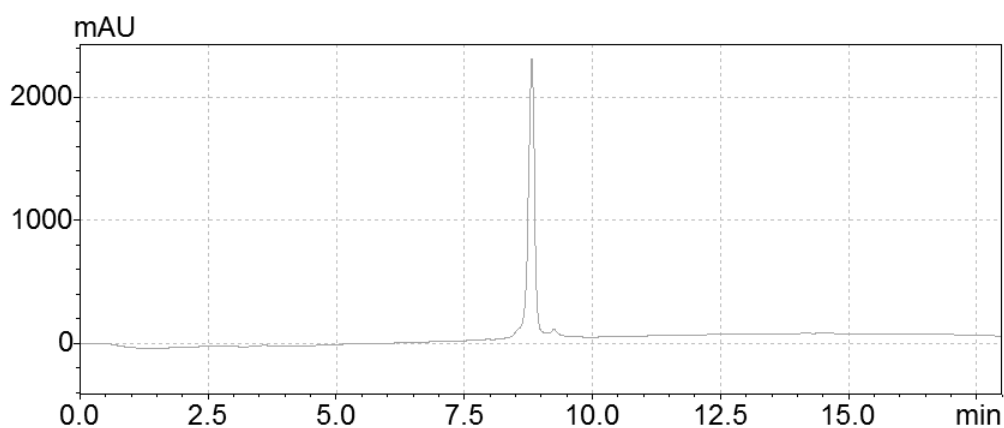

(2)

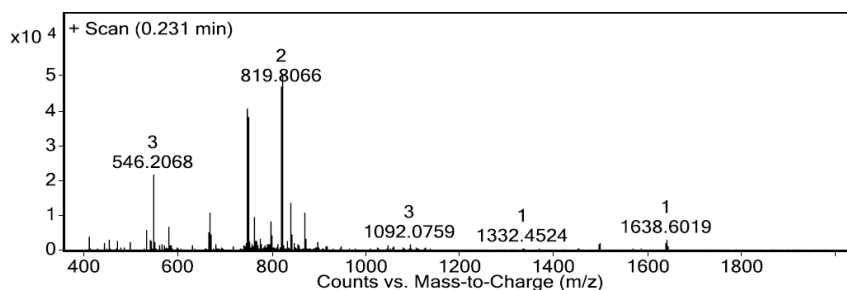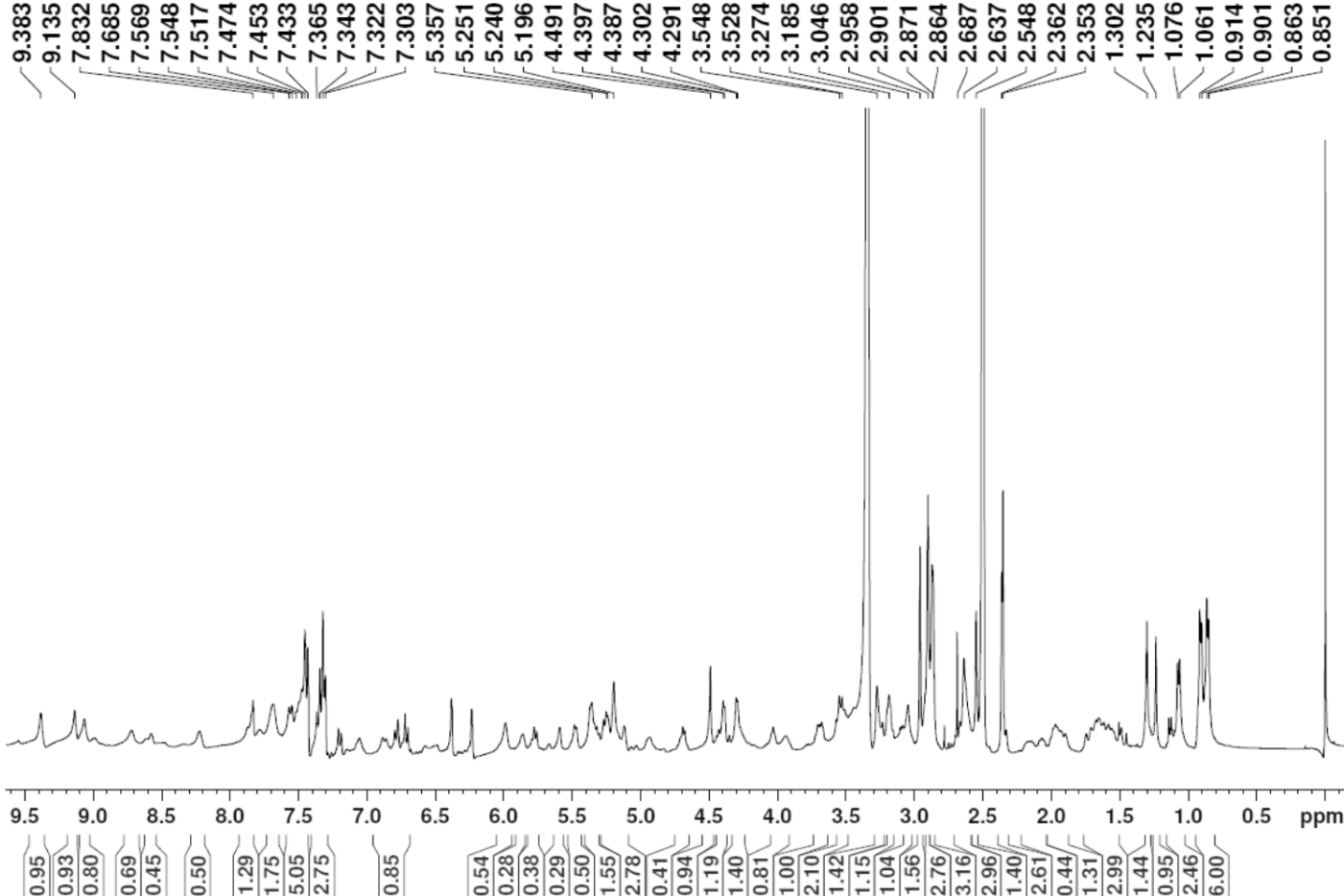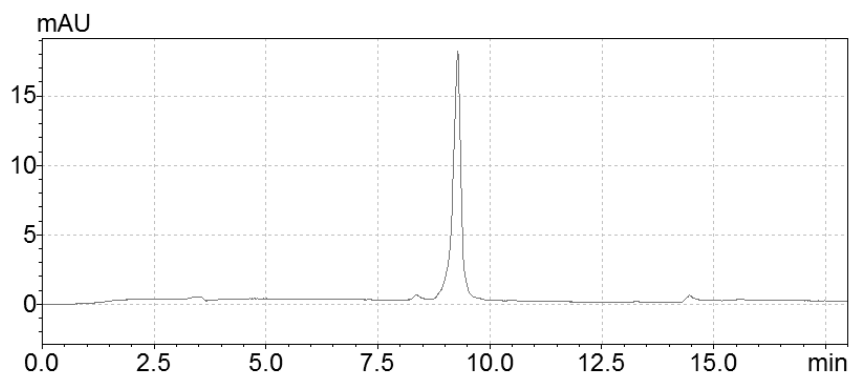

(3)

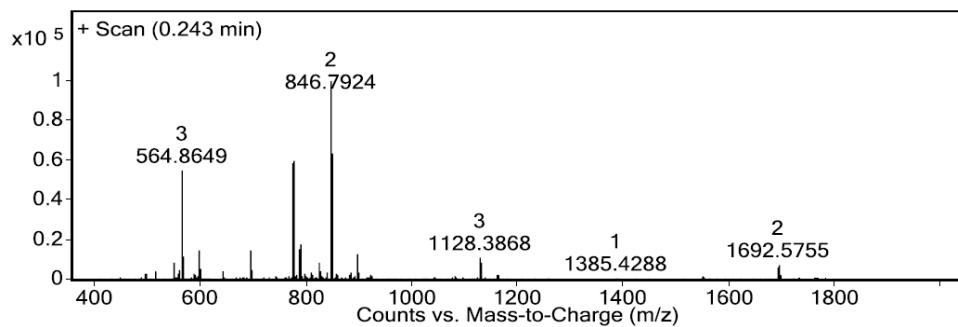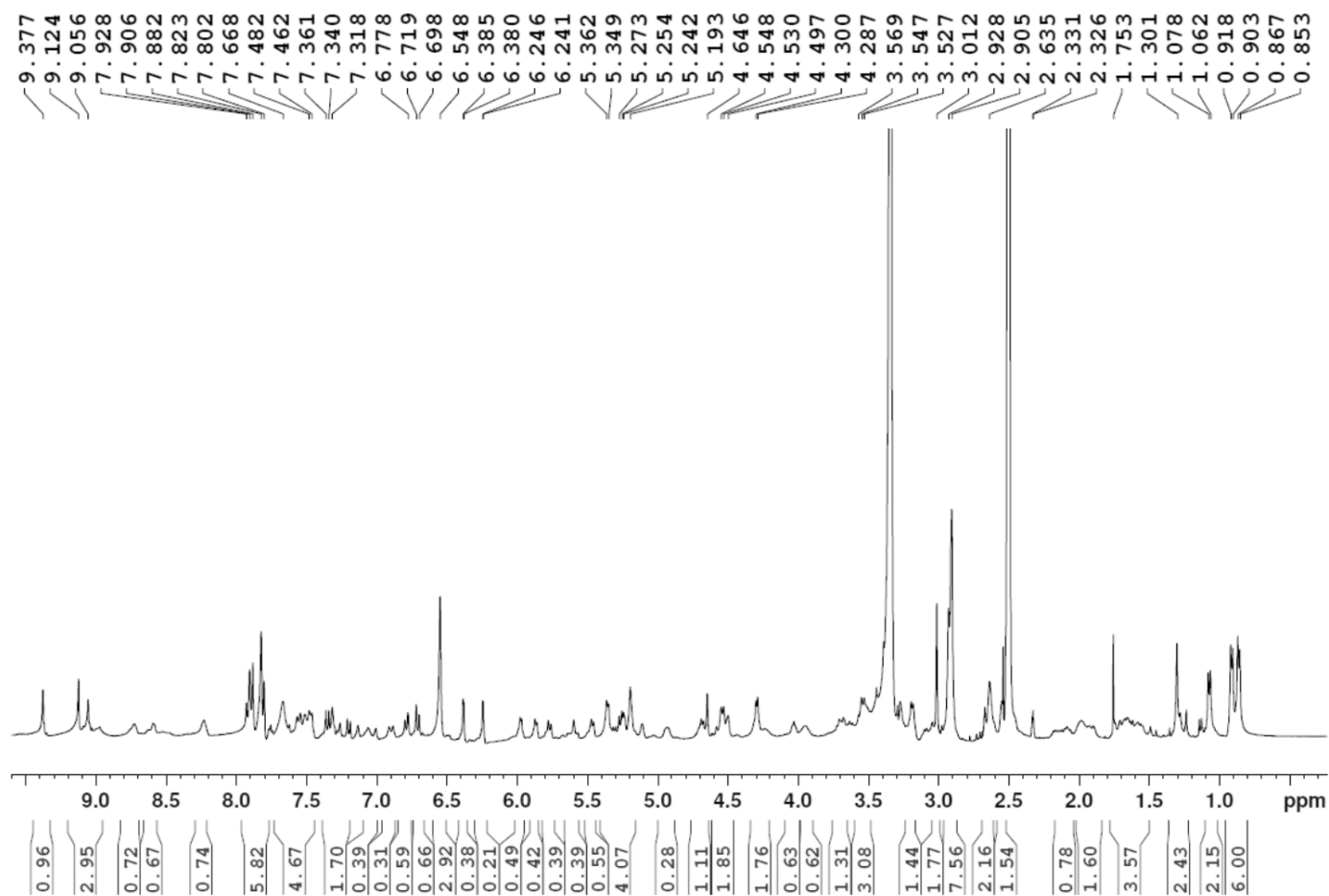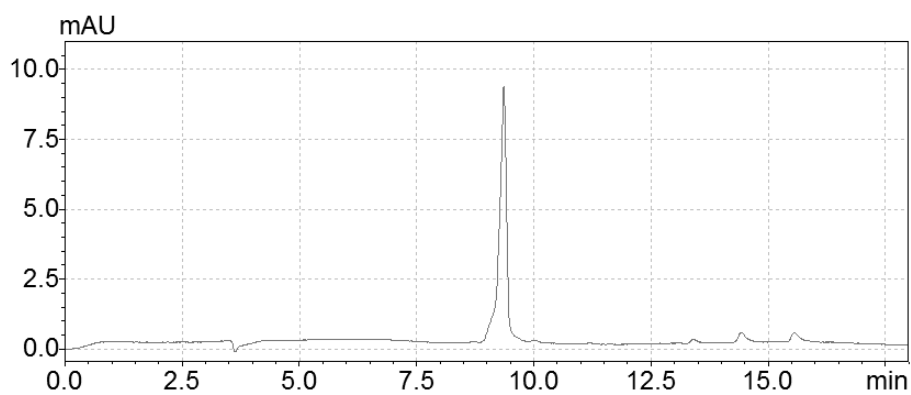

(4)

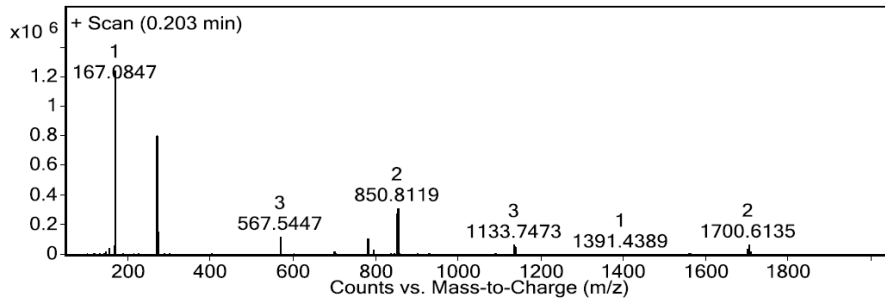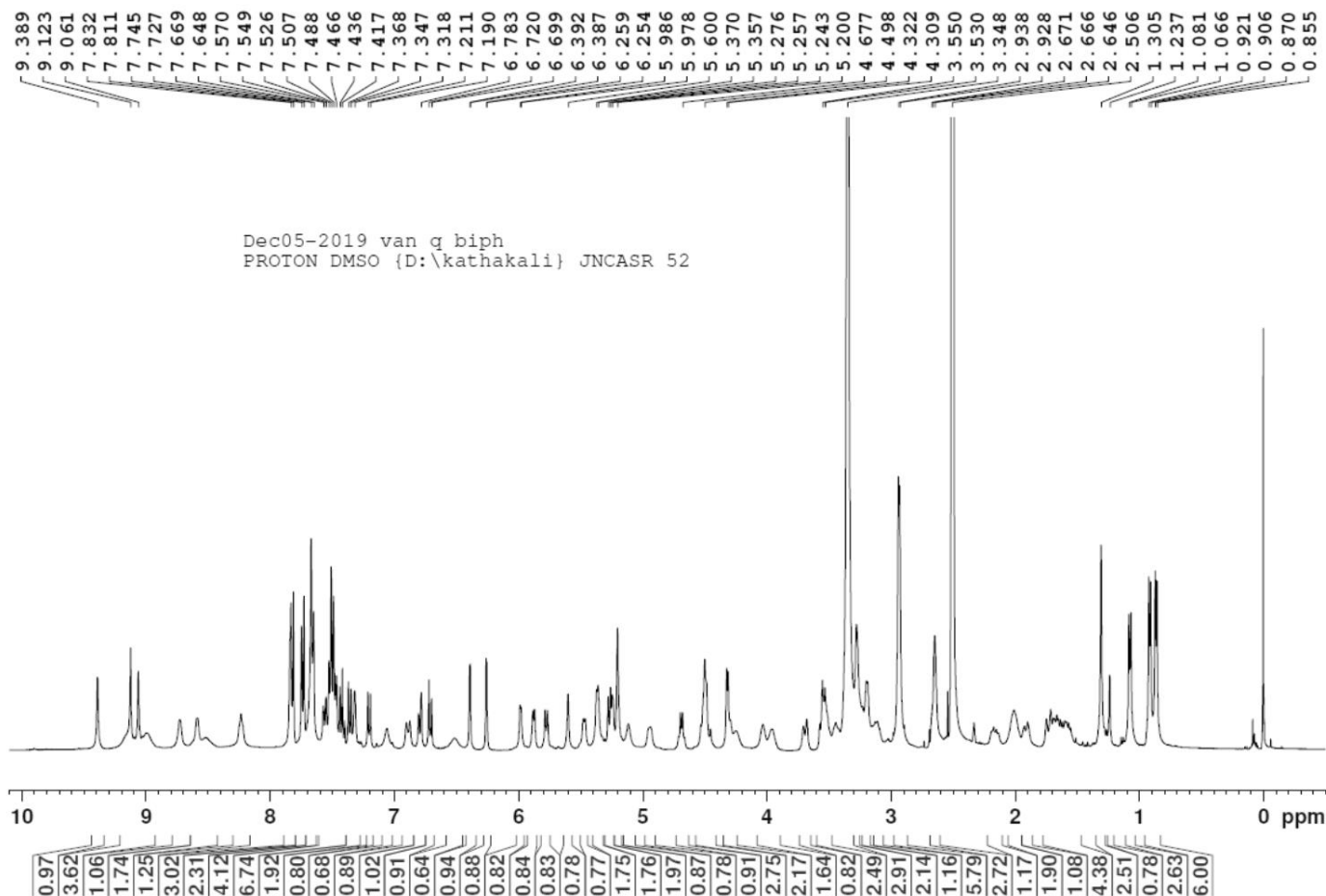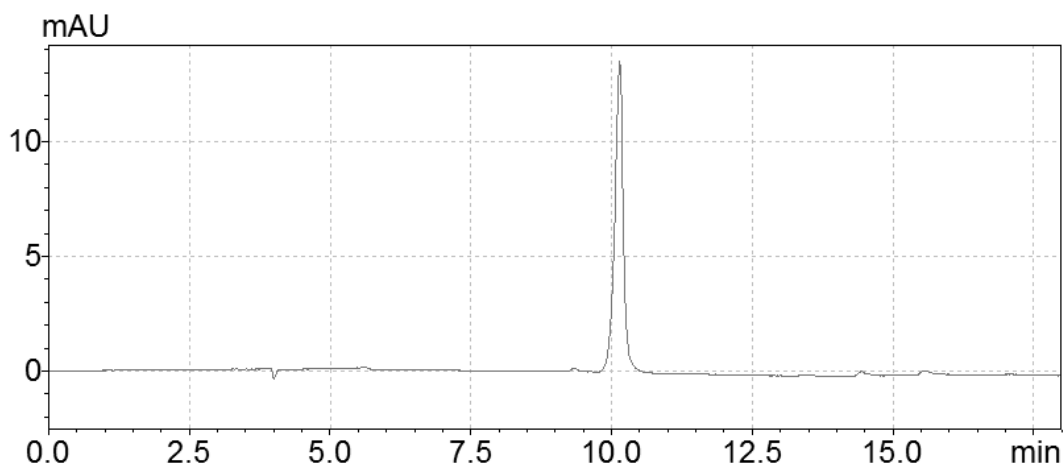

(5)

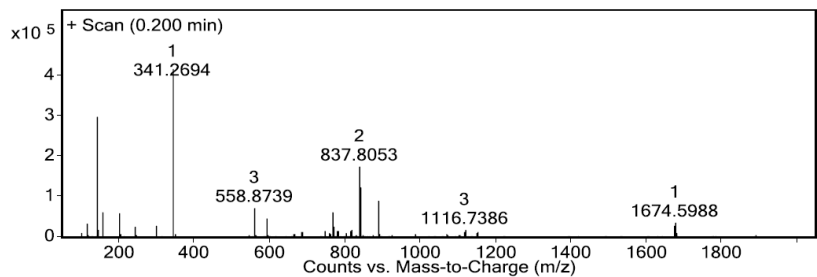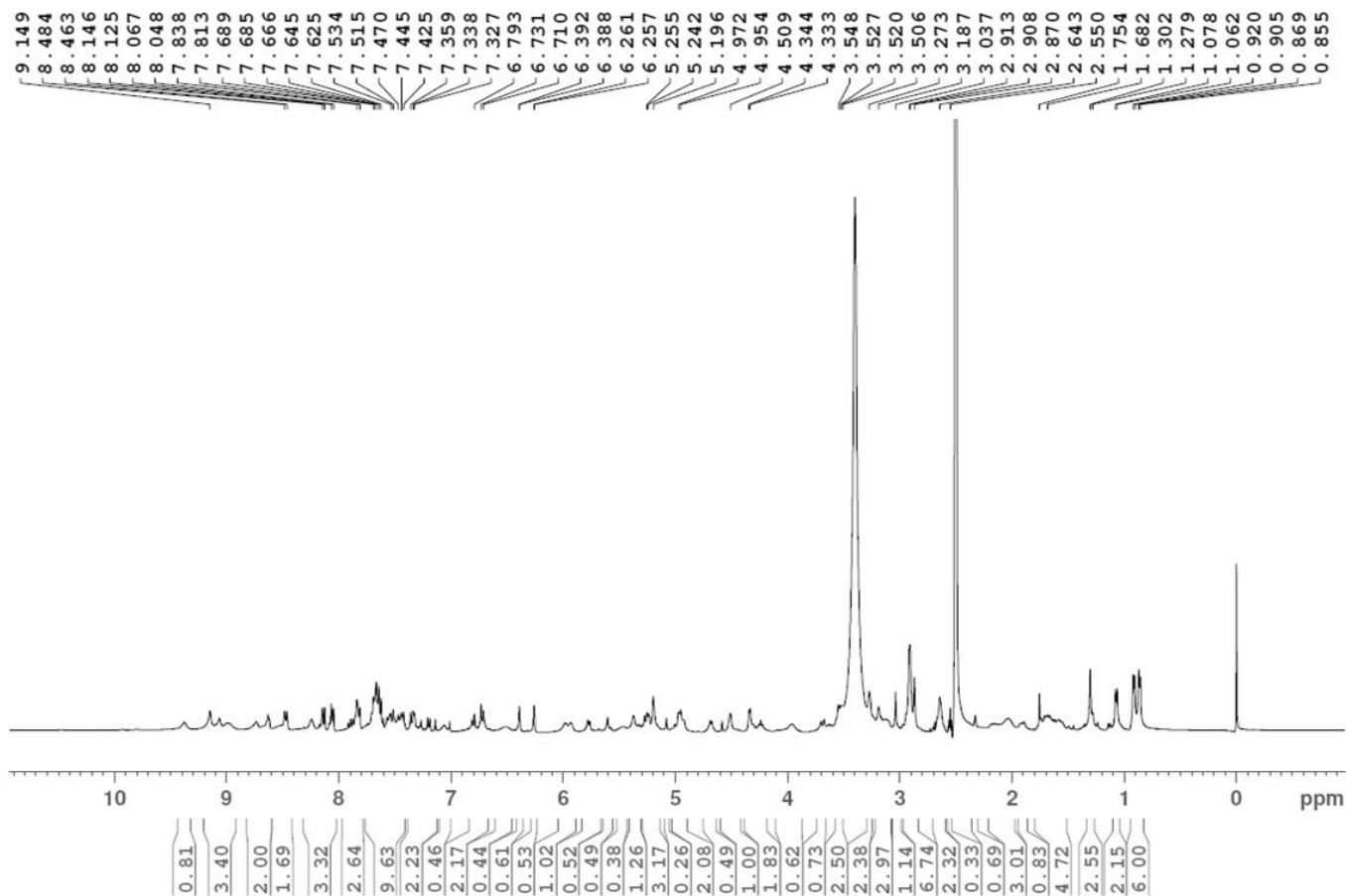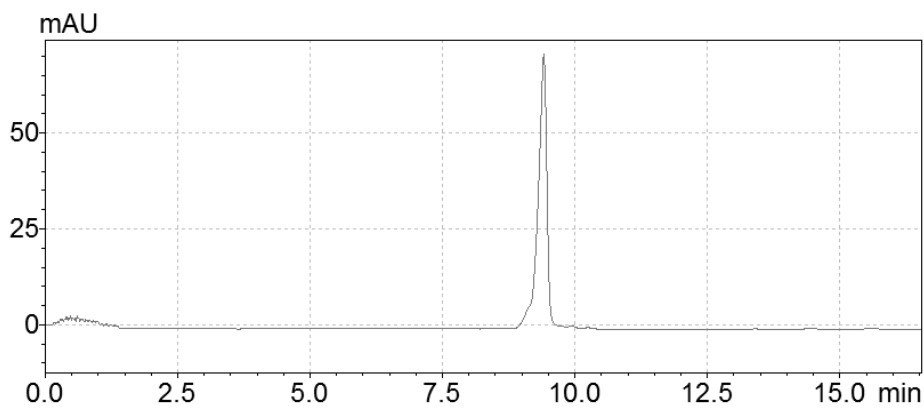

(6)

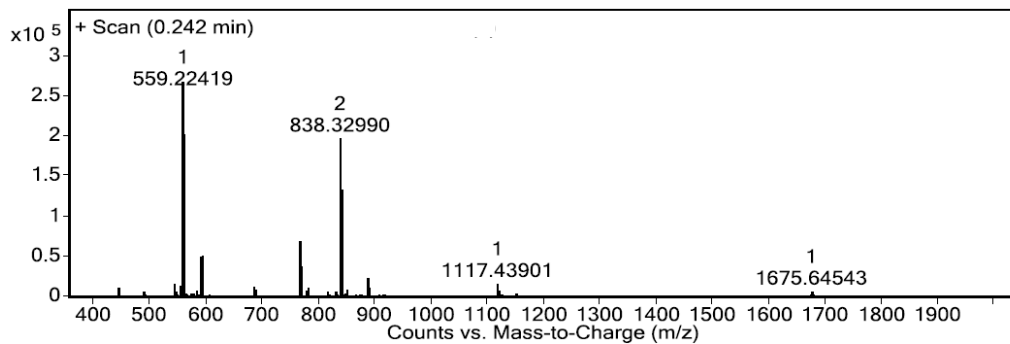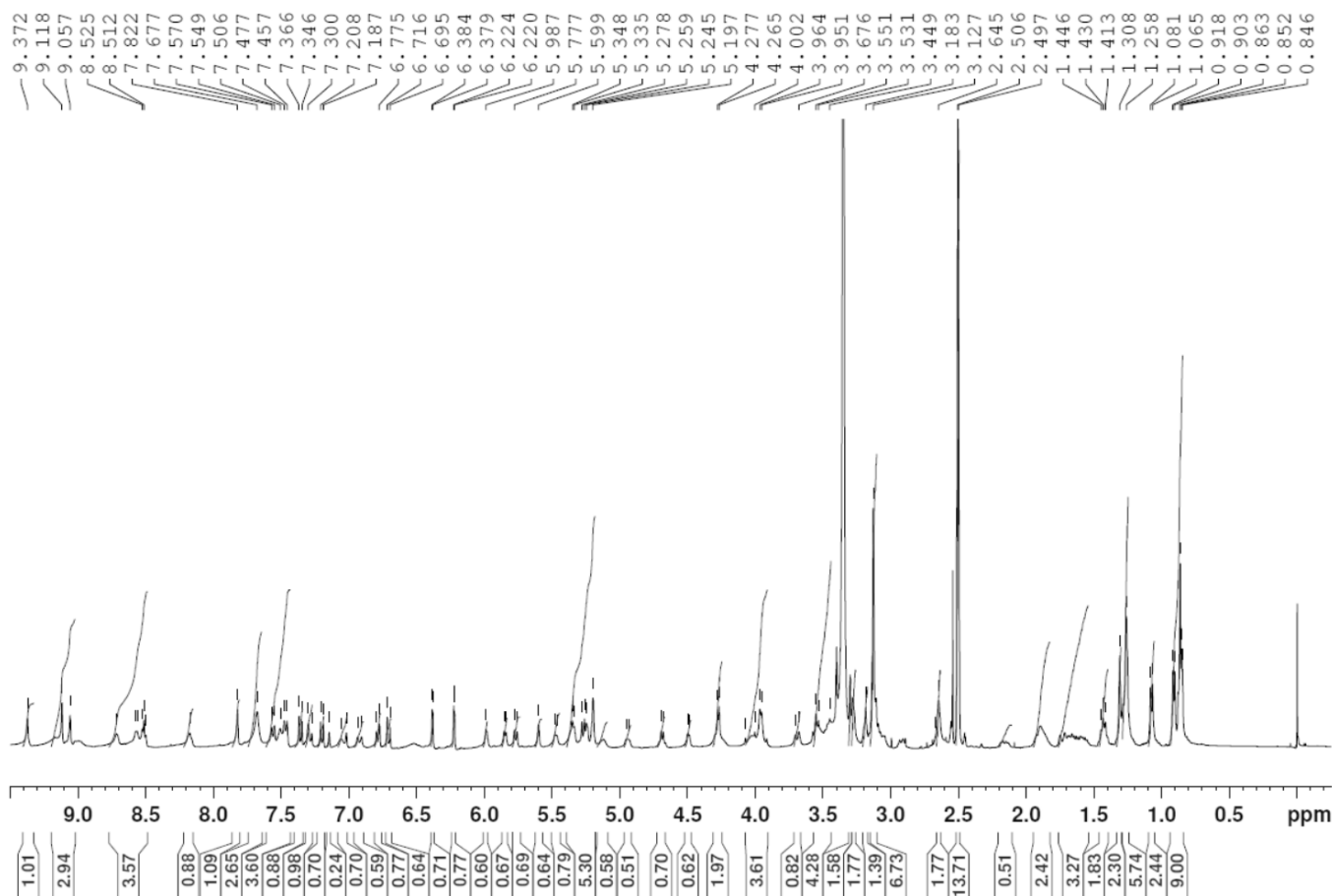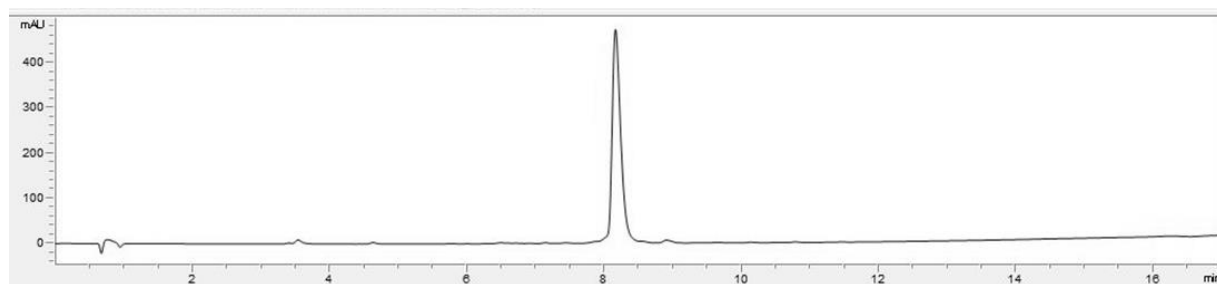

(7)

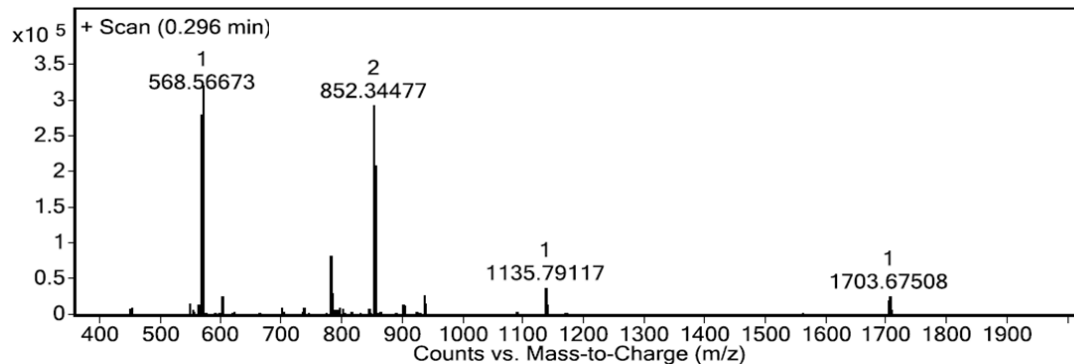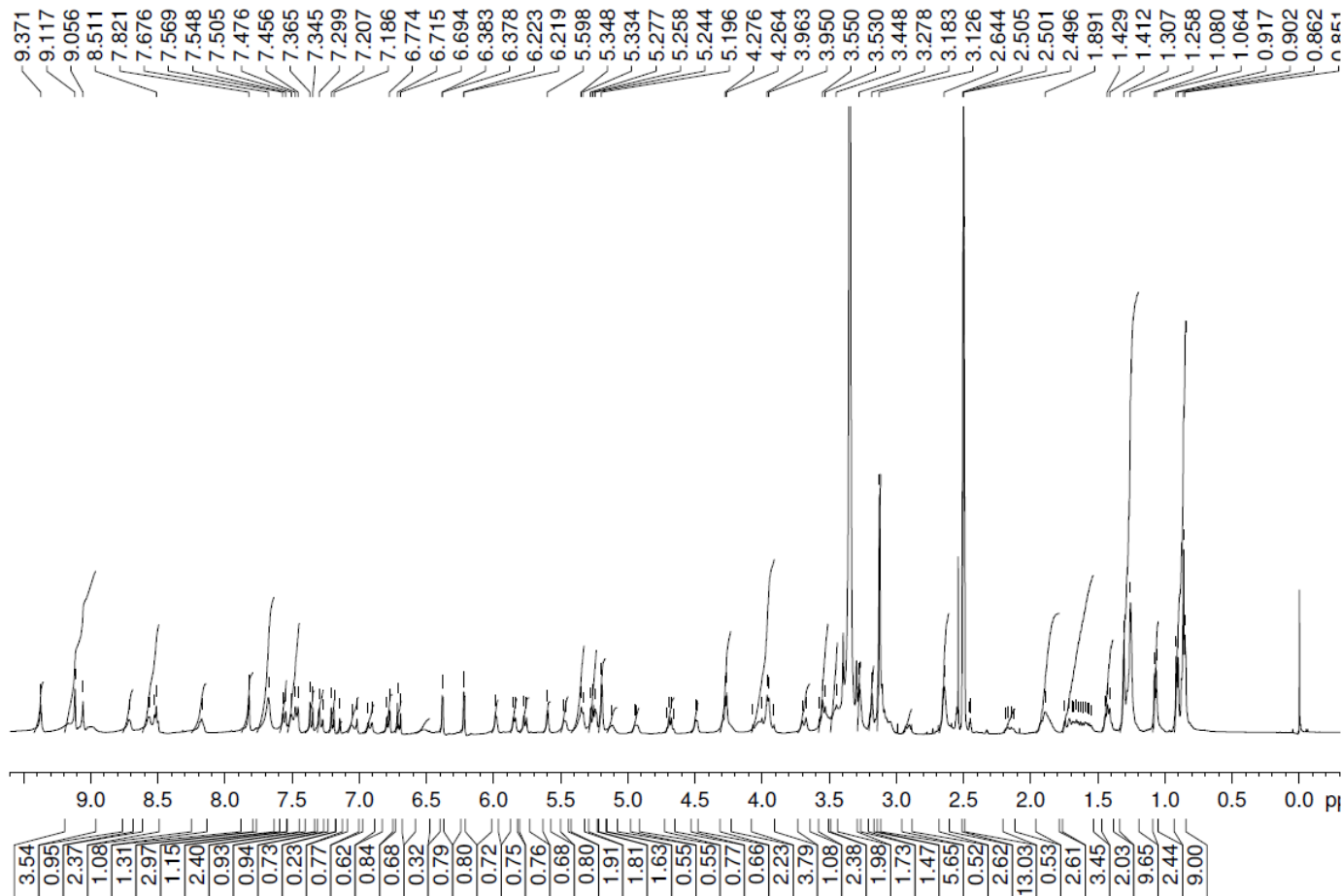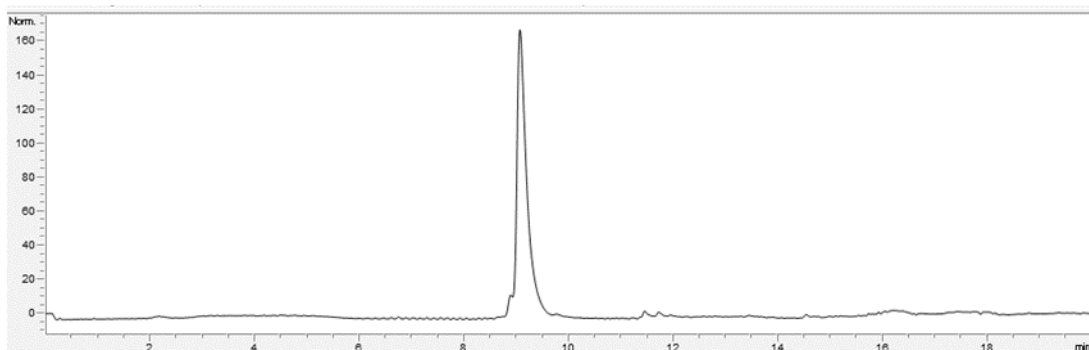

(8)

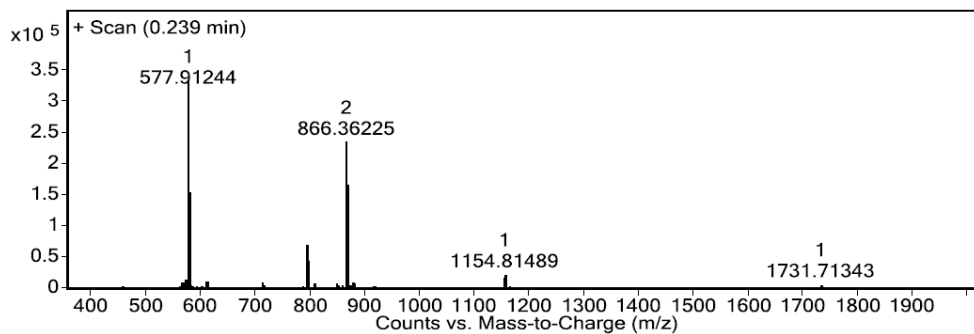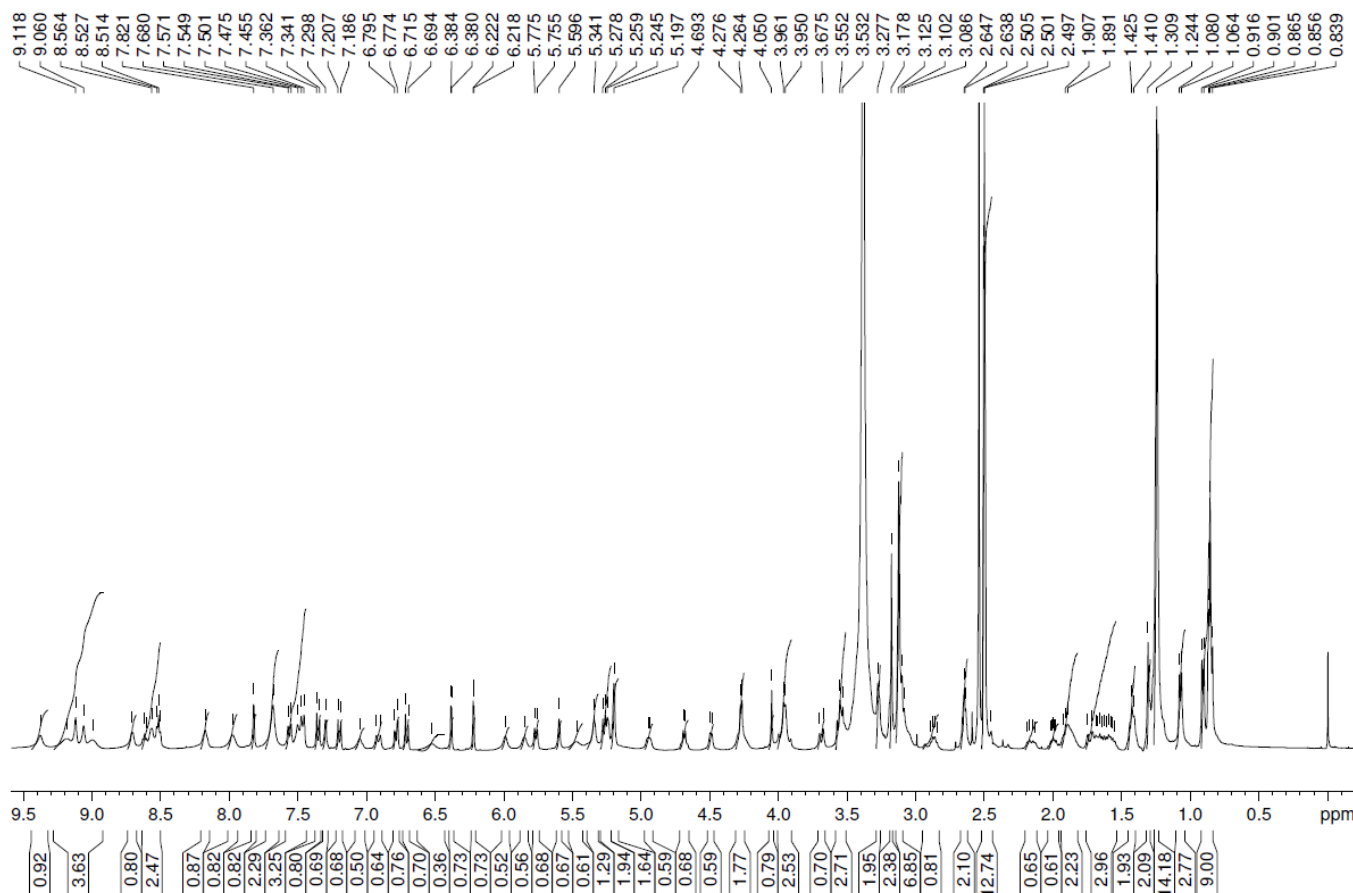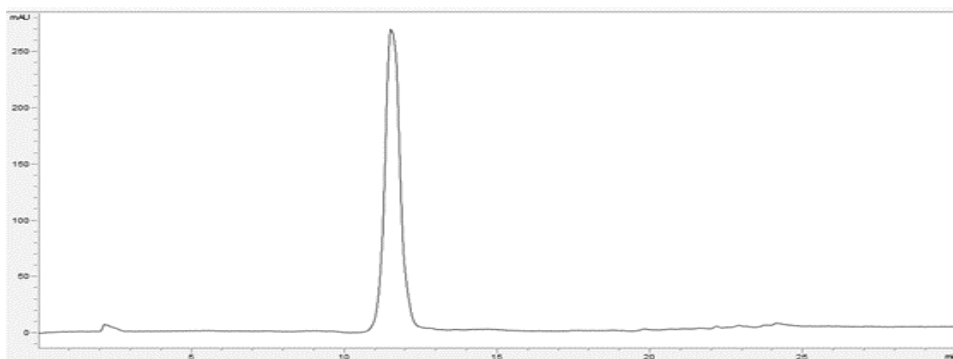

(9)

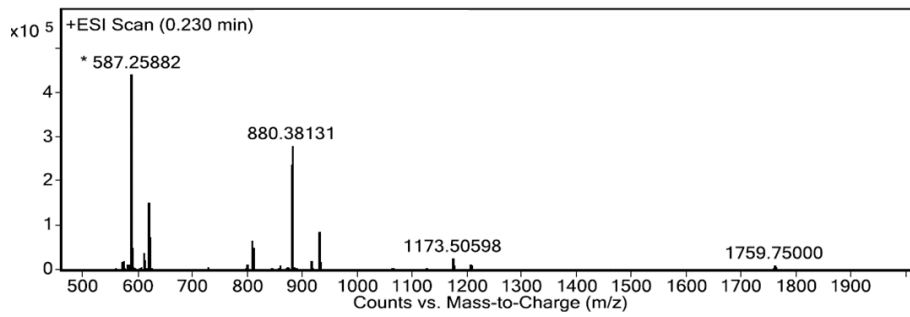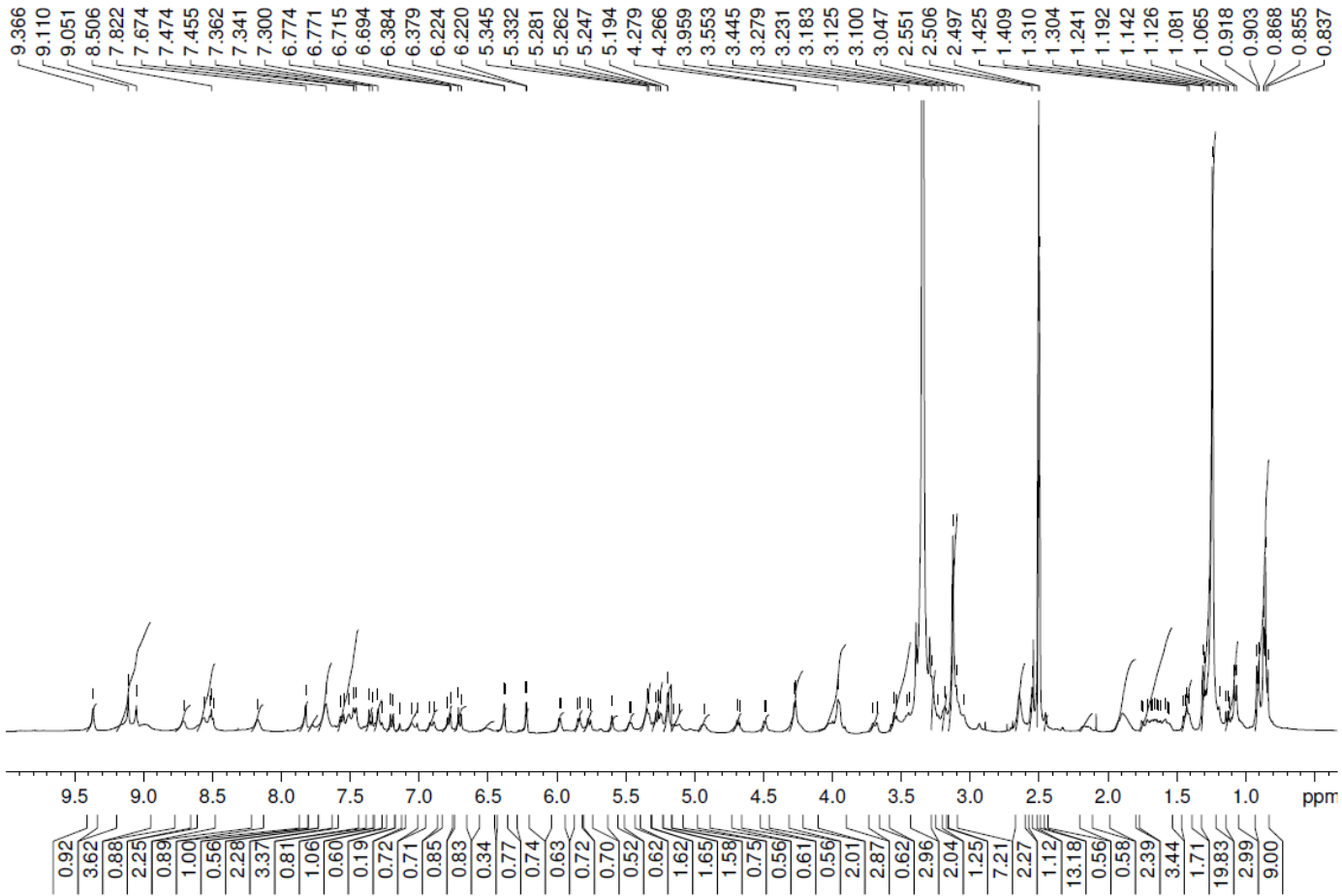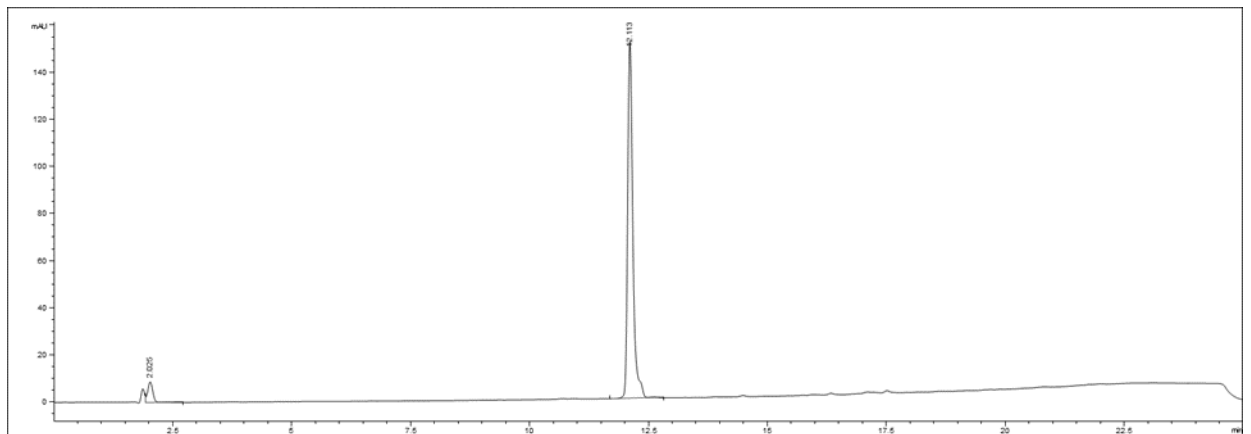

(10)

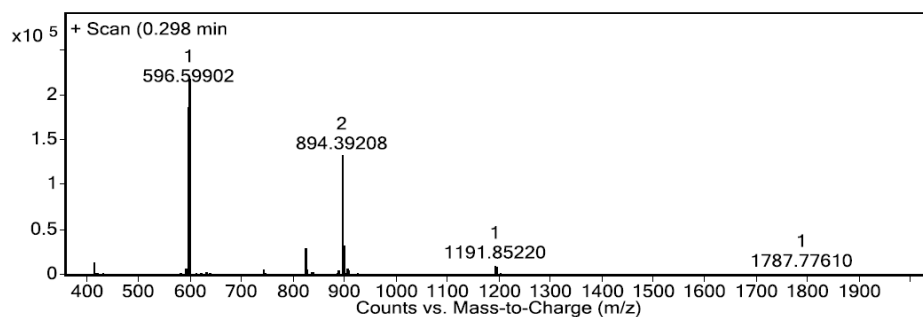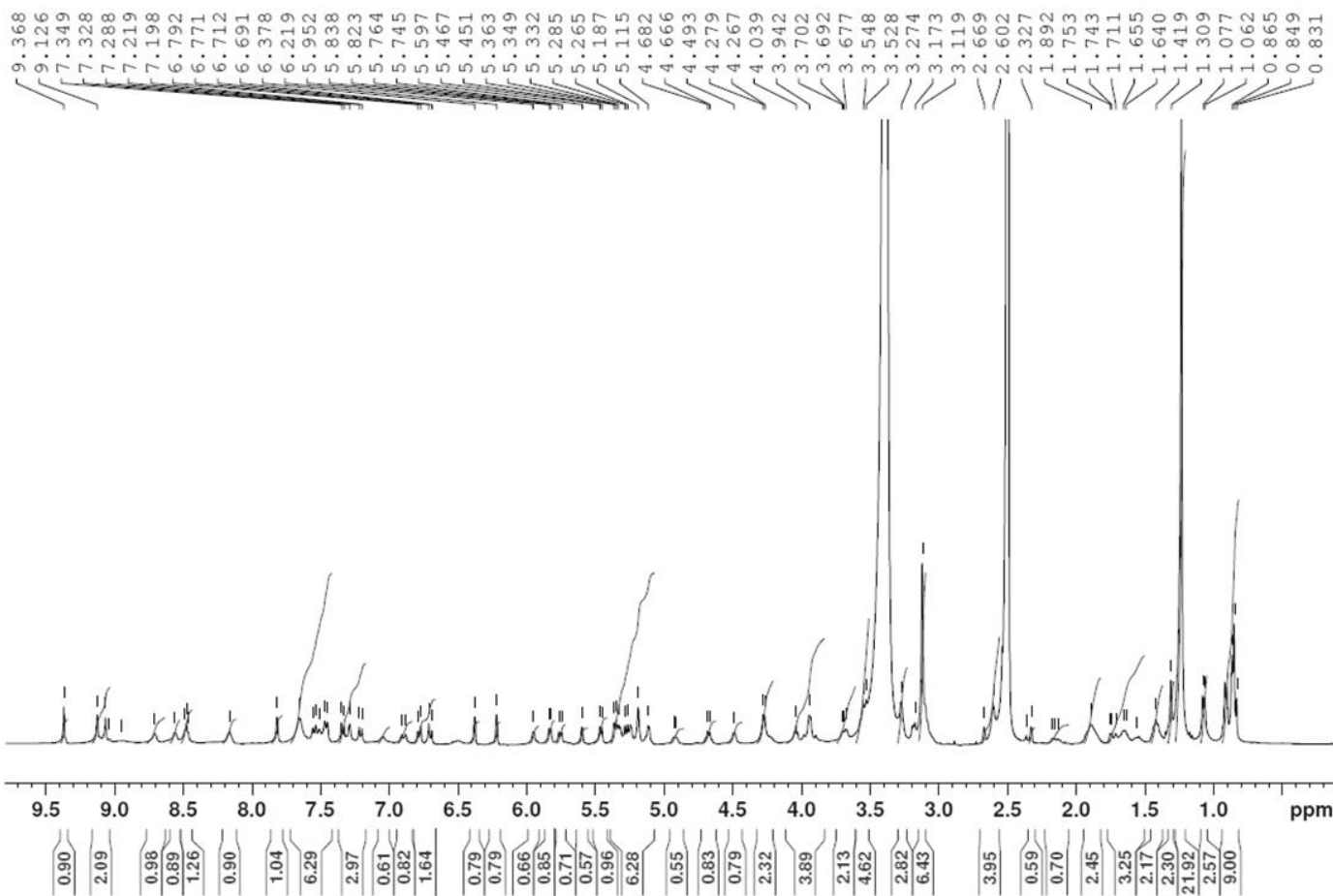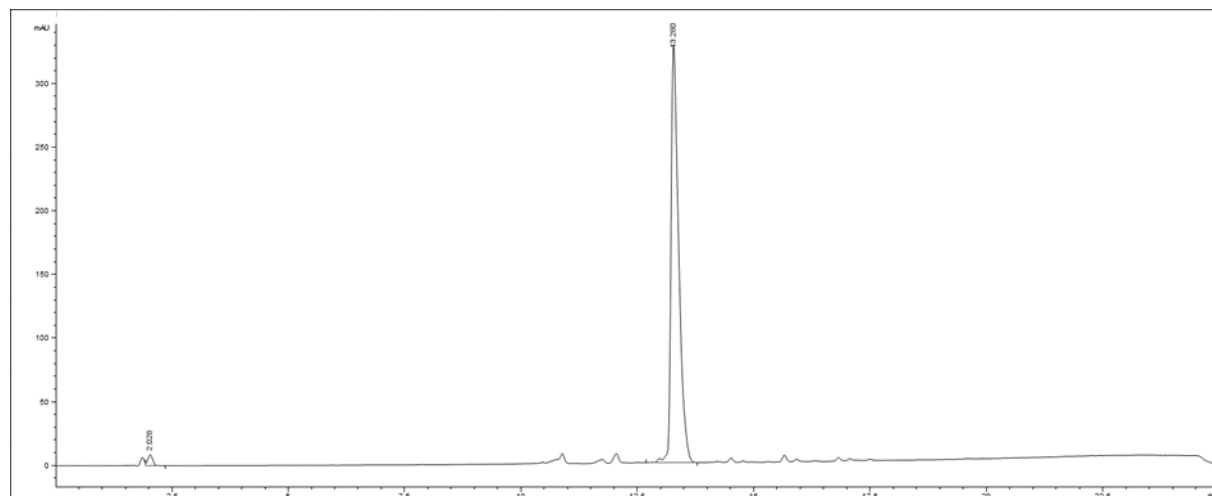

Supplement: SC-014-D2SC05600C-s001 [file SC-014-D2SC05600C-s001.pdf]
