# Supplementary material for: Next-generation membrane-active glycopeptide antibiotics that also inhibit bacterial cell division
Source: Chem Sci. 2023 Jan 6;14(9):2386–98. doi: 10.1039/d2sc05600c (PMC9977398; doi:10.1039/d2sc05600c)
Supplement: SC-014-D2SC05600C-s002 [file SC-014-D2SC05600C-s002.pdf]

Electronic Supplementary Material (ESI) for Chemical Science.
This journal is © The Royal Society of Chemistry 2023

Next-generation membrane-active glycopeptide antibiotics that also inhibit bacterial cell division

Paramita Sarkar,[a] Kathakali De,[a] Malvika Modi,[b] Geetika Dhanda,[a] Richa Priyadarshini,[b] Julia E. Bandow,[c] and Jayanta Haldar*[a]

[a]Antimicrobial Research Laboratory, New Chemistry Unit and School of Advanced Materials, Jawaharlal Nehru Centre for Advanced Scientific Research (JNCASR), Jakkur, Bengaluru 560064, Karnataka, India

[b]Department of Life Sciences, School of Natural Sciences, Shiv Nadar University, Dadri, 201314, UP, India

[c]Applied Microbiology, Faculty of Biology and Biotechnology, Ruhr University Bochum, Universitätsstraße 150, 44780 Bochum, Germany

*Corresponding author: Jayanta Haldar, Email ID: jayanta@jncasr.ac.in, Ph. No.: +91 802208 2565

Contents

Results- Page S2

Materials and methods -Page S4

Experimental Section- Page S6

Synthesis and characterization of compounds- Page S7

Biological assays-Page S16

References -Page S23

Results

Design and Synthesis

A series of lipophilic cationic moieties with varying hydrophobicity were synthesised through a simple synthetic strategy (Scheme 1). The aromatic substituents with varying hydrophobicity and lipophilicity selected were benzyl-, 4-methyl-benzyl-, 4-(trifluoromethyl)benzyl-, biphenyl- and naphthalen-1-yl-methyl-groups. The aliphatic hydrophobic substitutions consisted of amido-alkyl moieties (hexyl-, octyl-, decyl-, dodecyl-, and tetradecyl-). The inclusion of an amide bond has been reported to impart additional hydrogen bonding capability to the bacterial lipids and improve the selectivity towards bacteria.[1] To synthesize the amido-alkyl-cationic precursor moieties, first, the respective amines (hexyl-, octyl-, decyl-, dodecyl-, tetradecyl amine) were reacted with bromoacetyl bromide to yield the corresponding activated bromides (**6a-10a**). Boc protected *N[1]*,*N[1]*-dimethylpropan-1,3-diamine was then subjected to nucleophilic substitution of the respective aryl- (**1a-5a**) or activated alkyl bromides (**6a-10a**), to yield aminium bromides **(1b-10b)**. Boc deprotection of these compounds yielded the aminium based lipophilic precursor moieties (**1c-10c**) that were then coupled to the carboxylic

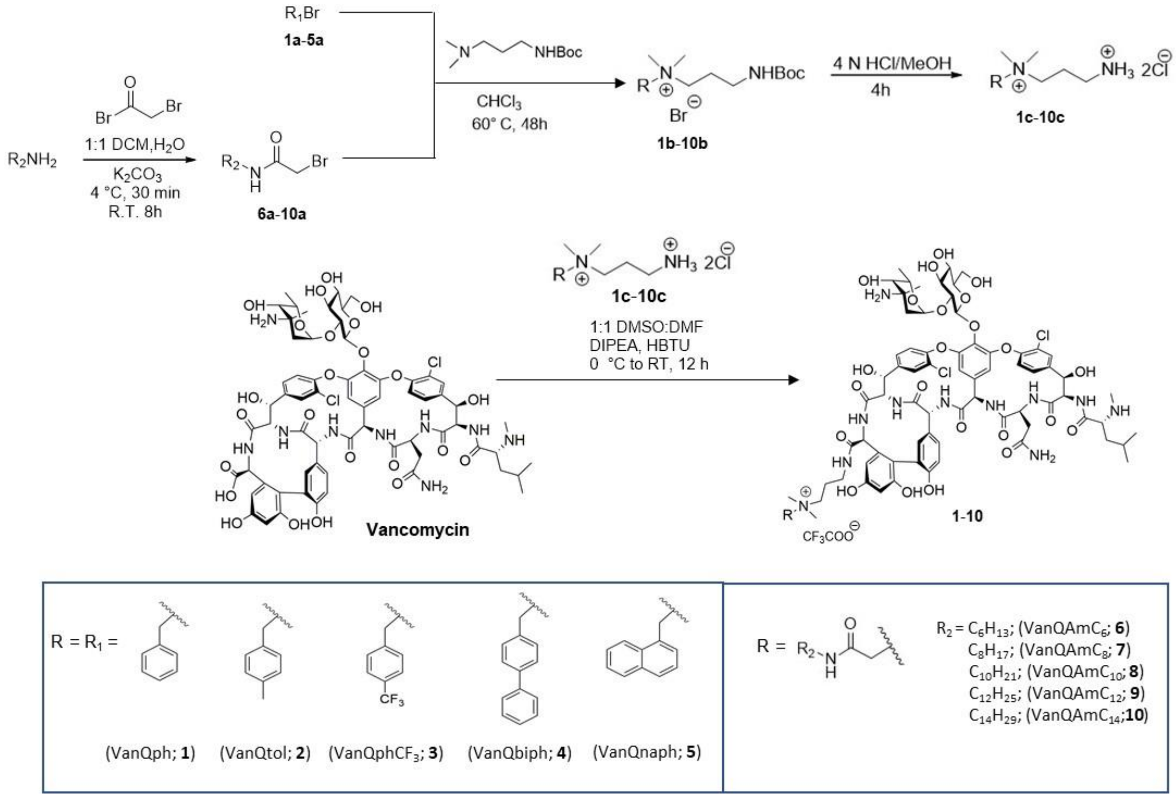

Scheme 1: Synthetic scheme for cationic lipophilic substitutions on C-terminus of vancomycin.

acid group of vancomycin to yield the compounds **1-10**. All the derivatives of vancomycin were purified by reverse phase HPLC to about 90 % purity with 65-75 % yield and characterized by HR-MS and ^{1}H-NMR.

Supplementary figures

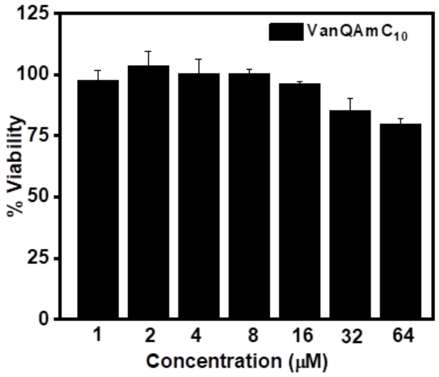

Figure S1. Cytotoxicity of VanQAmC$_{10}$ against MDCK cells. The CC$_{50}$ (defined as the concentration at which the viability of compound treated cells reduced to 50%) was greater than 64 µM.

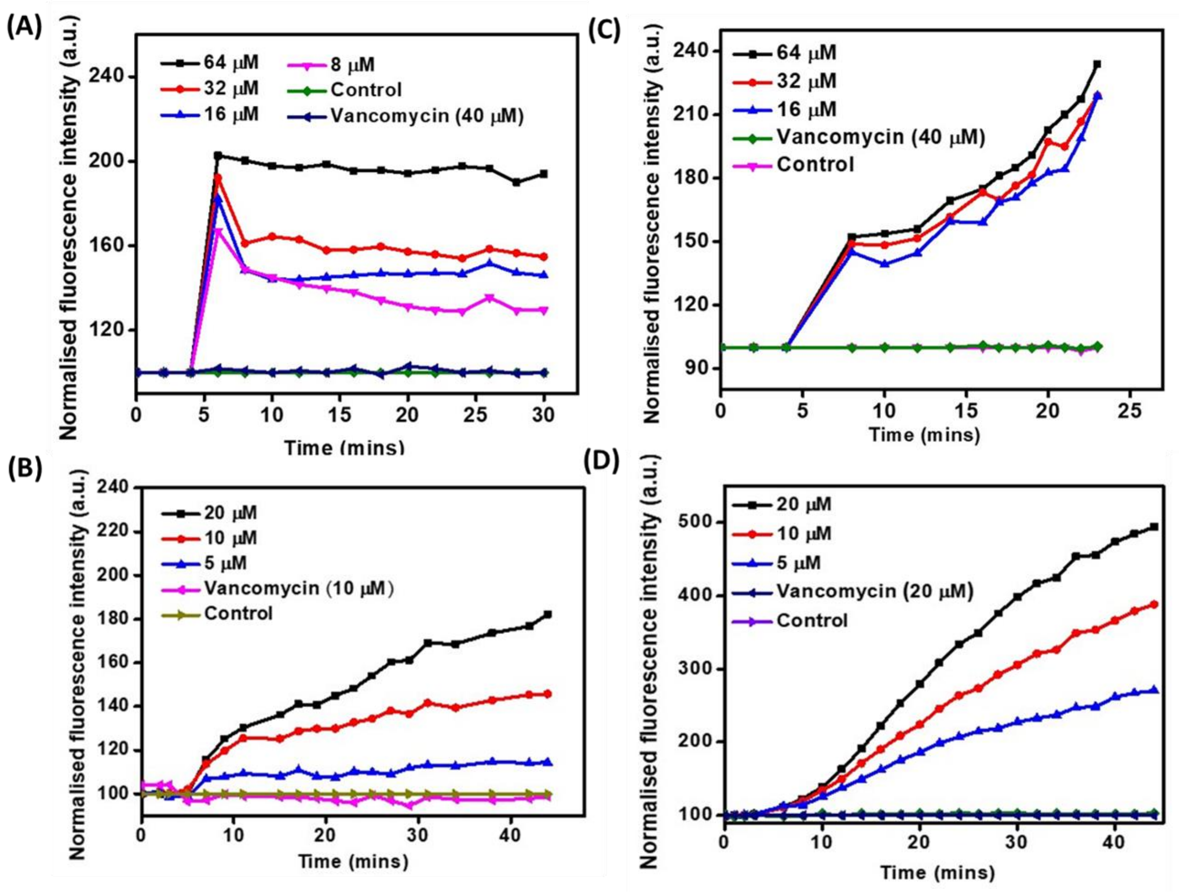

Figure S2. Concentration dependent membrane perturbation in exponentially growing MRSA caused by VanQAmC$_{10}$ and VanQbiph. Membrane depolarization by (A) VanQbiph and (B) VanQAmC10. Membrane permeabilization upon treatment with (C) VanQbiph and (D) VanQAmC$_{10}$.

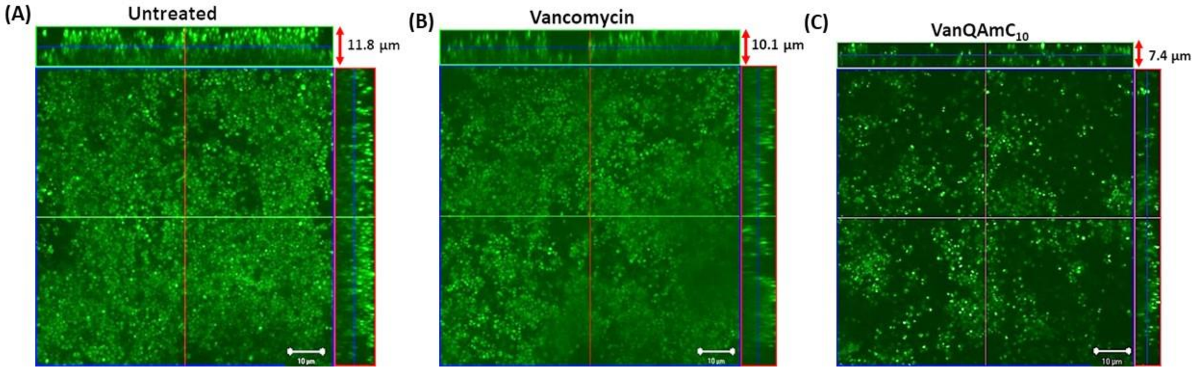

Figure S3. Efficacy of VanQAmC$_{10}$ (**8**) against biofilms of MRSA. Confocal laser scanning microscopy when mature biofilms were (A) untreated (B) treated with vancomycin at 20 μM, (C) treated with VanQAmC$_{10}$ at 20 μM; (Scale bar = 10 μm).

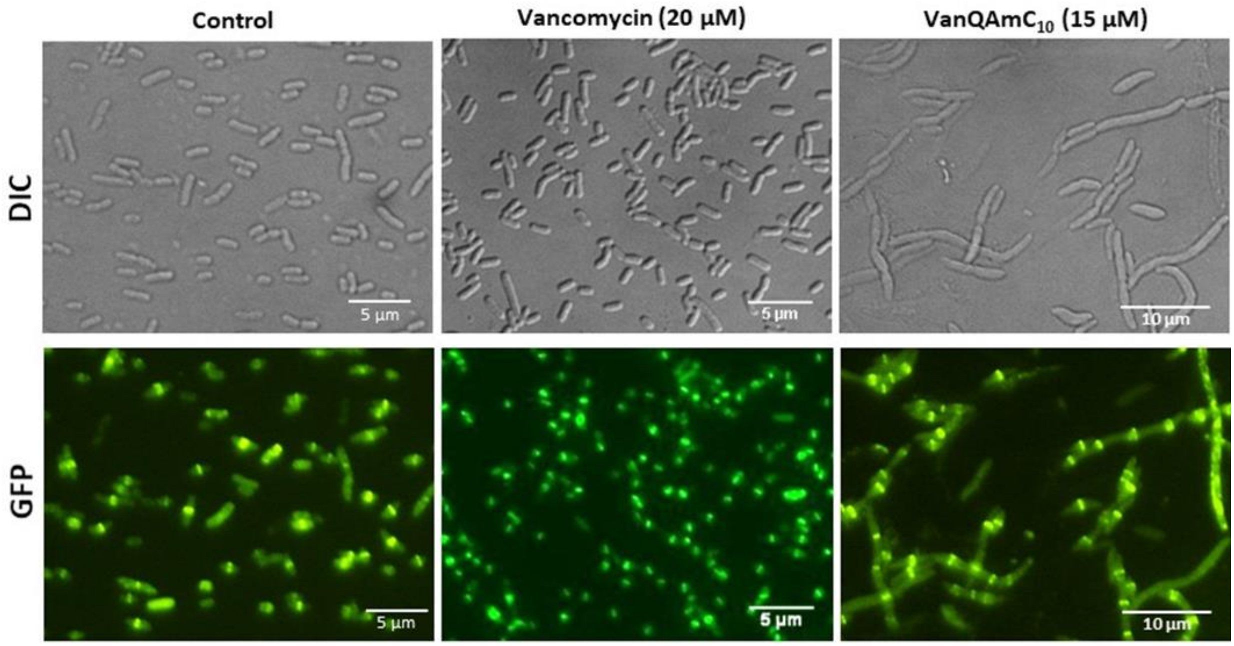

Figure S4. Light and fluorescence microscopy in GFP-FtsZ expressing *E. coli* to assess morphological changes and localization of FtsZ post-treatment with vancomycin (20 µM) and VanQAmC10 (15 µM) for 130 mins.

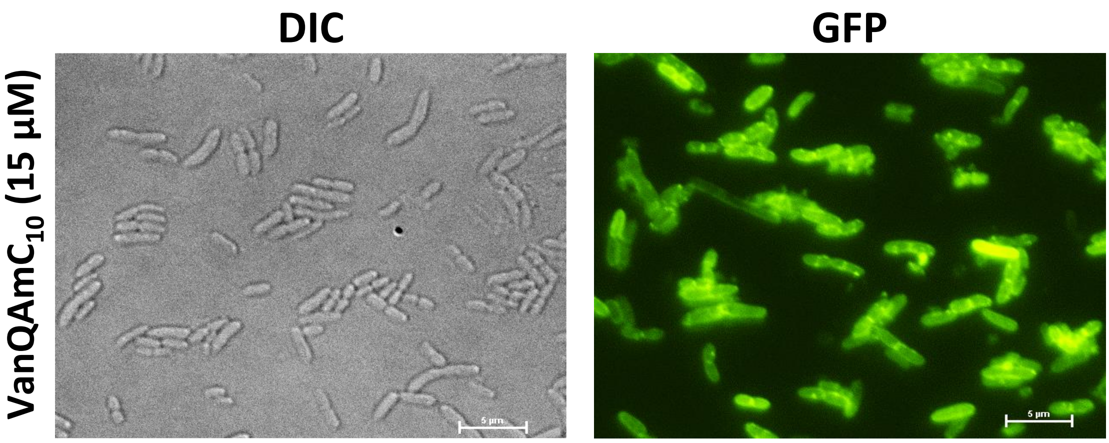

Figure S5. Fluorescence and light microscopy of GFP-FtsI producing *E. coli* post-treatment with 15 µM of VanQAmC$_{10}$.

Table S1: Activity of $VanQAmC_{10}$ in mouse plasma and liver homogenate against MRSA ATCC 33591 and VRE ATCC 51575

Bacteria	MIC of $VanQAmC_{10}$ post incubation in (μM)		
	Plasma	Liver homogenate	Media
VRE ATCC 51575	4	4	4
MRSA ATCC 33591	0.4	0.4	0.4

In-vivo toxicity

The LD_{50} of $VanQAmC_{10}$ (**8**) was found to be 70 mg/kg through intravenous injection. When administered intraperitoneally, a 130 mg/kg dose was found to be well tolerated and all mice survived (LD_{50} greater than 130 mg/kg). The LD_{50} of $VanQAmC_{10}$ was greater than 160 mg/kg when administered subcutaneously. The toxicity study in mice demonstrates the lower toxicity of $VanQAmC_{10}$ as compared to the previously developed molecule, $VanQC_{14}$.[3] This reduced toxicity of $VanQAmC_{10}$ can be attributed to the presence of the amide spacer between the cationic moiety and the alkyl chain as mentioned in the previous section.

Table S2 *In-vivo* toxicity (LD_{50} values) of $VanQAmC_{10}$ by various modes of administration

Mode of administration	LD_{50} (mg/kg)
Intravenous	70
Intraperitonial	>130
Subcutaneous	>160

Materials and methods

All reagents were purchased from Sigma-Aldrich and Spectrochem and used without further purification. All the solvents of reagent grade were purchased from Spectrochem and SD Fine. Chloroform for column chromatography was purchased from SD Fine chemicals and distilled prior to use. HPLC grade acetonitrile was purchased from Spectrochem. Analytical thin layer chromatography (TLC) was performed on E. Merck TLC plates pre-coated with silica gel 60 F254 (250 μm thickness). Vancomycin derivative was purified by reverse phase HPLC using 0.1 % trifluoroacetic acid (TFA) in water/acetonitrile (0-100 %) as mobile phase. HPLC

analysis was performed on a Shimadzu-LC 8 Å Liquid Chromatography instrument (C18 column, 10 mm diameter, 250 mm length) with UV detector monitoring at 254 nm. HPLC purification was performed at a flow rate of 8 mL/min (eluent A: H_2O + 0.1 % TFA, eluent B: acetonitrile + 0.1 % TFA) using the gradient of 5 % B to 95% B over 20 minutes and then at 95% B for another 10 minutes.

The NMR spectra were recorded using Bruker AMX-400 (400 MHz for 1H) spectrometer in deuterated solvents. The chemical shifts (δ) are reported in parts per million downfield from the peak for the internal standard TMS for 1H NMR. High-resolution mass spectra (HR-MS) were obtained using 6538-UHD Accurate Mass Q-TOF LC-MS instrument. TECAN (Infinite series, M200 pro) Plate Reader was used to measure absorbance in biological assays. A Zeiss 510 Meta confocal laser-scanning microscope was used for confocal imaging. Bacterial strains, MRSA ATCC 33591, Enterococcal strains were obtained from ATCC (Rockville, MD) and MTCC. Clinical isolates of VRSA were obtained from National Institute of Mental Health and Neuro Sciences (NIMHANS), Bengaluru, India and BEI resources.

Details of *E. coli* strains and plasmids:

S.no.	Strain	Relevant genotype or description	Reference
1.	RP21	MG1655 Δ*amiA::frt* Δ*amiC::frt* /pBAD18	Dubey A. *et al., Current Genetics*, **2018**, *64*, 661–675
Plasmids			
1.	pDSW230	Expressing *ftsZ-gfp*; Amp^r	Weiss D.S. *et al., J. Bacteriol.* **1999**, 181, 508–520.
2.	pDSW234	Expressing *gfp-ftsI*; Amp^r	Weiss D.S. *et al., J. Bacteriol.* **1999**, 181, 508–520.

Animals: Six-week old pathogen free Balb/c female mice weighing 20 to 24 g were used for in vivo studies. The animal experiments were approved by the Institutional Animal Ethics Committee (IAEC) and carried out as per the guidelines of Committee for the purpose of Supervision and Experiments on Animals (CPCSEA), Ministry of Environment and Forests, New Delhi.

Experimental Section

Synthesis and characterisation of compounds

Synthesis of *t*-Butyl (3-(dimethylamino)propyl)carbamate : N^1,N^1-Dimethylpropan-1,3-diamine (4 g, 1 equivalent) was dissolved in 1M NaOH solution and $(Boc)_2O$ (4 equivalent)

was added to it. The reaction mixture was stirred at room temperature for 10 h. The compound was then extracted into the organic layer using chloroform. The resultant organic solution was evaporated and dried to afford colourless oily tert-butyl (3-(dimethylamino)propyl)carbamate with 70 % yield.

^{1}H-NMR (400 MHz, CD_3OD) δ/ppm: 4.67 (s, 1H), 2.96-2.94 (s, 2H), 1.5-1.2 (m, 6H), 1.81 (s, 9H). HRMS *m/z:* 203.1737 ($[M+H]^+$ observed), 203.1755 (calculated).

General procedure for synthesis of 6a-10a: Alkyl amine (2 g, 1 equiv) was dissolved in dichloromethane (DCM) and stirred at 4 °C. K_2CO_3 (1.5 equiv) was dissolved in 10 mL Millipore water and added to the alkyl amine solution. Bromoacetyl bromide (1.5 equiv) was then dissolved in dry DCM and added drop-wise into the reaction mixture at 4 °C, over 30 minutes. The reaction mixture was then stirred at room temperature for 8 h. The product was extracted in chloroform and the chloroform was evaporated under reduced pressure to obtain the pure product with 85-95% yield.

2-Bromo-*N*-hexylethanamide (6a): Yield 95%; ^{1}H-NMR (400 MHz, $CDCl_3$) δ/ppm: 6.53 (s, 1H; CON*H*), 3.87 (s, 2H; $COCH_2Br$), 3.29-3.24 (m, 2H; $CONHCH_2$), 1.56-1.49 (m,2H; $NHCH_2CH_2C_4H_9$), 1.29 (bs, 6H; $CH_2(CH_2)_3CH_3$), 0.89-0.86 (m, CH_3,3H); HRMS *m/z:* 222.0489 ($[M+H]^+$, observed), 222.0495 (calculated).

2-Bromo-*N*-octyl-ethanamide (7a): Yield 90%; ^{1}H-NMR (400 MHz, $CDCl_3$) δ/ppm: 6.52 (s, 1H; N*H*CO), 3.86 (s, 2H; $COCH_2Br$), 3.29-3.24 (m, 2H; $CONHCH_2$), 1.55-1.48 (m, 2H; $CONHCH_2CH_2C_6H_{13}$), 1.29-1.26 (m, 10H; ($CH_{2(C8\ alkyl\ chain)}$)), 0.86 (t, *J* = 7.2 Hz, 3H; CH_3); HRMS *m/z:* 250.0898 ($[M+H]^+$, observed), 250.0878 (calculated).

2-Bromo-*N*-decyl-ethanamide (8a): Yield 97%; ^{1}H-NMR (400 MHz, $CDCl_3$) δ/ppm: 6.52 (s,1H; N*H*CO), 3.87 (s, 2H; $COCH_2Br$), 3.27 (dd, *J* = 13.2 Hz, 7.2 Hz, 2H; $CONHCH_2$), 1.56-1.49 (m, 2H; $NHCH_2CH_2C_8H_{17}$), 1.25 (bs, 14H; $CH_{2(C10\ alkyl\ chain)}$), 0.87 (t, *J* = 7.2 Hz, CH_3, 3H); HRMS *m/z:* 278.1111 ($[M+H]^+$, observed), 278.1120 (calculated)

2-Bromo-*N*-dodecyl-ethanamide (9a): Yield 98%; ^{1}H-NMR (400 MHz, $CDCl_3$) δ/ppm: 6.46 (s,1H; N*H*CO), 3.88 (s, 2H; $COCH_2Br$), 3.28 (dd, *J* = 13.2 Hz, 6.8 Hz, 2H; $CONHCH_2$), 1.53 (dd, *J* = 14.4 Hz, 7.2 Hz, 2H; $CONHCH_2CH_2C_{10}H_{21}$), 1.26 (m, 18H; $CH_{2(C10\ alkyl\ chain)}$), 0.88 (t, *J* = 6.8 Hz, 3H; CH_3); HRMS *m/z:* 306.1409 ($[M+H]^+$, observed), 306.2822 (calculated)

2-Bromo-*N*-tetradecyl-ethanamide (10a): Yield 97%; ^{1}H-NMR (400 MHz, $CDCl_3$) δ/ppm: 6.47 (s,1H; N*H*CO), 3.88 (s, 2H; COC*H*$_2$Br), 3.28 (dd, *J* = 13.2 Hz, 7.2 Hz, 2H; CONHC*H*$_2$), 1.55-1.52 (m, 2H; CONHCH$_2$C*H*$_2$C$_{12}$H$_{25}$), 1.26 (m, 22H; C*H*$_{2\text{(C14 alkyl chain)}}$), 0.88 (t, *J* = 6.8 Hz, 3H; C*H*$_3$); HRMS *m/z:* 334.1655 ($[M+H]^+$, observed), 334.3354 (calculated)

General procedure for synthesis of 1b-10b: Compounds **6a-10a** (2 equiv) or the respective aryl bromides were dissolved in dry chloroform in a sealed tube and NHBoc-*N,N*-dimethyl propylamine (1g, 1 eqv) was added to it. The reaction mixture was allowed to reflux for 48 h. The pure products were obtained by column chromatography ($CHCl_3$/CH_3OH) using Silica gel as aminium salts.

***N*-benzyl-3-((tert-butoxycarbonyl)amino)-*N,N*-dimethylpropan-1-aminium bromide (1b):** Yield 69%; ^{1}H-NMR (400 MHz, $CDCl_3$) δ/ppm: 7.56-7.50 (m, 5H; *H*$_{Ar}$), 6.96 (t, *J* = 5.6 Hz, 1H; *H*$_{Ar}$), 4.59 (s, 2H; C*H*$_{2(Ar)}$N$^+$), 3.27- 3.23 (m, 2H; N$^+$C*H*$_2$CH$_2$), 3.02 (s, 2H; C*H*$_2$NHBoc), 2.98 (s, 6H; N$^+$(C*H*$_3$)$_2$), 1.93 (t, *J* = 8.0 Hz, 2H; C*H*$_2$CH$_2$NHBoc), 1.37 (s, 9H; OC(C*H*$_3$)$_3$); HRMS *m/z*: 293.2211 (observed), 293.2224 (calculated for M$^+$, $C_{17}H_{29}N_2O_2^+$).

3-((tert-butoxycarbonyl)amino)-*N,N*-dimethyl-*N*-(4-(trifluoromethyl)benzyl)propan-1-aminium bromide (2b): Yield 70%; ^{1}H-NMR (400 MHz, $CDCl_3$) δ/ppm: 7.87 (d, *J* =7.2 Hz 2H; *H*$_{Ar}$), 7.69 (d, *J* =7.0 Hz, 2H; *H*$_{Ar}$), 5.45 (bs, 1H; N*H*Boc), 5.18 (s, 2H; C*H*$_{2(Ar)}$N$^+$), 3.67 (bs, 2H; N$^+$C*H*$_2$CH$_2$), 3.31 (s, 6H; N$^+$(C*H*$_3$)$_2$), 3.28-3.23 (m, 2H; CH$_2$C*H*$_2$NHBoc), 2.22-2.12 (m, 2H; C*H*$_2$CH$_2$NHBoc), 1.4 (s, 9H; O-C(C*H*$_3$)$_3$); HRMS *m/z*: 361.2098 (Observed), 361.2098 (calculated for M$^+$, $C_{18}H_{28}F_3N_2O_2^+$).

3-((tert-butoxycarbonyl)amino)-*N,N*-dimethyl-N-(4-methylbenzyl)propan-1-aminium bromide (3b): Yield 65%; ^{1}H-NMR (400 MHz, $CDCl_3$) δ/ppm: 7.42 (d, *J* = 7.8 Hz, 2H; *H*$_{Ar}$), 7.30 (d, , *J* = 7.4 Hz 2H; *H*$_{Ar}$), 6.96 (t, *J* = 7.2 Hz 1H; N*H*Boc), 4.49 (s, 2H; C*H*$_{2(Ar)}$N$^+$), 3.22-3.17 (m, 2H; N$^+$C*H*$_2$CH$_2$), 3.03-2.97 (m, 3H; PhC*H*$_3$), 2.94 (s, 6H; N$^+$(C*H*$_3$)$_2$), 2.35 (s, 2H; CH$_2$C*H*$_2$NHBoc), 1.95-1.85 (m, 2H; C*H*$_2$CH$_2$NHBoc), 1.37 (s, 9H; O-C(C*H*$_3$)$_3$); HRMS *m/z*: 307.2379 (Observed), 307.2380 (calculated for M$^+$, $C_{18}H_{31}N_2O_2^+$).

***N*-([1,1'-biphenyl]-4-ylmethyl)-3-((tert-butoxycarbonyl)amino)-*N,N*-dimethylpropan-1-aminium bromide (4b):** Yield 70%; ^{1}H-NMR (400 MHz, $CDCl_3$) δ/ppm: 7.70 (d, *J* = 8.0 Hz, 2H; *H*$_{Ar}$), 7.64 (d, *J* = 8.0 Hz, 2H; *H*$_{Ar}$), 7.54 (d, *J* = 7.2 Hz, 2H; *H*$_{Ar}$), 7.46-7.38 (m, 3H; *H*$_{Ar}$), 5.54 (bs, 1H; N*H*Boc), 4.99 (s, 2H; C*H*$_{2(Ar)}$N$^+$), 3.73 (bs, 2H; C*H*$_2$NHBoc), 3.30 (bs, 8H;

$N^+(CH_3)_2$ and $CH_2CH_2N^+$), 2.20- 2.17 (m, 2H; CH_2CH_2NHBoc), 1.41 (s, 9H; O-C(CH_3)$_3$); HRMS *m/z*: 369.2519 (Observed), 369.2537 (calculated for M^+, $C_{23}H_{33}N_2O_2^+$).

3-((tert-butoxycarbonyl)amino)-*N*,*N*-dimethyl-*N*-(naphthalen-1-ylmethyl)propan-1-aminium bromide (5b): Yield 66%; ^{1}H-NMR (400 MHz, $CDCl_3$) δ/ppm: 8.53 (d, *J* = 7.2 Hz, 1H; CON*H*), 8.15 (d, *J* = 8.0 Hz, 2H; H_{Ar}), 8.06 (d, *J* = 8.0 Hz, 2H; H_{Ar}), 7.82 (d, *J* = 7.2 Hz, 2H; H_{Ar}), 7.70-7.60 (m, 3H; H_{Ar}), 6.98 (t, *J* = 5.6 Hz, 1H; H_{Ar}), 5.06 (s, 2H; $CH_{2(Ar)}N^+$), 3.47-3.43 (m, 2H; BocNHCH_2CH_2), 2.99 (bs, 8H; $N^+(CH_3)_2$ and $CH_2CH_2N^+$), 1.98 (m, 2H; CH_2CH_2NHBoc), 1.38 (s, 9H; O-C(CH_3)$_3$); HRMS *m/z*: 344.2364 (observed), 344.2360 (calculated for M^+, $C_{21}H_{33}N_2O_2^+$).

3-((tert-butoxycarbonyl)amino)-*N*-(2-(hexylamino)-2-oxoethyl)-*N*,*N*-dimethylpropan-1-aminium bromide (6b): Yield 70%; ^{1}H-NMR (400 MHz, $CDCl_3$) δ/ppm: 8.85 (bs, 1H; CON*H*C_6H_{13}), 5.20 (s, 1H; N*H*Boc), 4.56 (s, 2H; N^+CH_2CONHC_6H_{13}), 3.68 (d, *J* = 7.6 Hz, 2H; BocNH(CH_2)$_2$$CH_2N^+$), 3.36 (s, 6H; $N^+(CH_3)_2$), 3.29-3.24 (m, 4H; BocNH-CH_2CH_2 and CONH-CH_2), 2.14-2.10 (m, 2H; $N^+CH_2CH_2CH_2$NHBoc), 1.62-1.54 (m, 2H; CONH$CH_2CH_2C_4H_9$), 1.44 (s, 9H; O-C(CH_3)$_3$), 1.30-1.26 (m, 6H; $CH_{2(\text{C6 alkyl chain})}$), 0.88 (t, *J* = 7.2 Hz, 3H; CH_3); HRMS *m/z*: 344.2898 (observed), 344.2908 (calculated for M^+).

3-((tert-butoxycarbonyl)amino)-*N*,*N*-dimethyl-*N*-(2-(octylamino)-2-oxoethyl)propan-1-aminium bromide (7b): Yield 65%; ^{1}H-NMR (400 MHz, $CDCl_3$) δ/ppm: 8.89 (bs, 1H; CON*H*C_8H_{17}), 5.11 (s, 1H; N*H*Boc), 4.54 (s, 2H; $N^+(CH_3)_2CH_2$CONHC_8H_{17}), 3.65 (t, *J* = 8.0 Hz, 2H; BocNH$CH_2CH_2N^+$), 3.35 (s, 6H; $N^+(CH_3)_2$), 3.29-3.24 (m, 4H; BocNHCH_2CH_2 and CONHCH_2), 2.16-2.09 (m, 2H; $N^+CH_2CH_2CH_2$NHBoc), 1.57-1.55 (m, 2H; -CONH$CH_2CH_2C_6H_{13}$), 1.44 (s, 9H; ,OC(CH_3)$_3$), 1.33-1.26 (m, 10H; $CH_{2(\text{C8 alkyl chain})}$), 0.87 (t, *J* = 6.8 Hz, CH_3, 3H); HRMS *m/z*: 372.3308 (observed), 372.3221(calculated for M^+).

3-((tert-butoxycarbonyl)amino)-*N*-(2-(decylamino)-2-oxoethyl)-*N*,*N*-dimethylpropan-1-aminium bromide (8b): Yield 68%; ^{1}H-NMR (400 MHz, $CDCl_3$) δ/ppm: 8.86 (bs, 1H; CON*H*$C_{10}H_{21}$), 5.15 (s, 1H; N*H*Boc), 4.55 (s, 2H; N^+CH_2CONH$C_{10}H_{21}$), 3.67 (t, *J* = 8.0 Hz, 2H; BocNH$CH_2CH_2N^+$), 3.35 (s, 6H; $N^+(CH_3)_2$), 3.27 (t, *J* = 6.0 Hz, 4H; BocNHCH_2CH_2 and CONHCH_2), 2.14-2.10 (m, 2H; $N^+CH_2CH_2CH_2$NHBoc), 1.60-1.55 (m, 2H; CONH$CH_2CH_2C_8H_{17}$), 1.44 (s, 9H; OC(CH_3)$_3$), 1.33-1.35 (m, 14H; $CH_{2(\text{C10 Alkyl chain})}$), 0.87 (t, *J* = 8.0 Hz, 3H; CH_3); HRMS *m/z*: 400.3588 (observed), 400.3534 (calculated for M^+).

3-((tert-butoxycarbonyl)amino)-*N*-(2-(dodecylamino)-2-oxoethyl)-*N*,*N*-dimethylpropan-1-aminium bromide (9b): Yield 70%; ^{1}H-NMR (400 MHz, $CDCl_3$) δ/ppm: 8.85 (t, J = 5.6 Hz, 1H; N*H*Boc), 5.14 (s, 1H;CON*H*$C_{12}H_{25}$), 4.58 (s, 2H; $N^+CH_2CONHC_{12}H_{25}$), 3.68 (t, J = 8.0 Hz, 2H; $BocNHCH_2CH_2N^+$), 3.36 (s, 6H; $N^+(CH_3)_2$), 3.28-3.23(m, 4H; BocNH-CH_2CH_2 and CONH-CH_2), 2.16-2.09 (m, 2H; $N^+CH_2CH_2CH_2$NHBoc), 1.6-1.55 (m, 2H; $CONHCH_2CH_2C_{10}H_{23}$), 1.44 (s, 9H; $OC(CH_3)_3$), 1.25 (bs, 18H; $CH_{2(C12\ Aryl\ chain)}$), 0.87 (t, J = 8.0 Hz, 3H; CH_3); HRMS *m/z*: 428.3805 (M^+ observed), 428.3847(M^+ calculated).

3-((tert-butoxycarbonyl)amino)-*N*,*N*-dimethyl-N-(2-oxo-2-(tetradecylamino)ethyl) propan-1-aminium bromide (10b): Yield 72%;^{1}H-NMR (400 MHz, $CDCl_3$) δ/ppm: 8.87 (bs,1H; CON*H*$C_{14}H_{29}$,), 5.13 (s, 1H; -N*H*Boc), 4.57 (s,2H; $N^+CH_2CONHC_{14}H_{29}$), 3.67 (t, J = 7.6 Hz, 2H; $BocNHCH_2CH_2N^+$), 3.36 (s, 6H; $N^+(CH_3)_2$), 3.29-3.24 (m, 4H; $BocNHCH_2CH_2$ and $CONHCH_2$), 2.14-2.10 (m, 2H; $N^+CH_2CH_2CH_2$NHBoc), 1.62-1.53 (m, 2H; $CONHCH_2CH_2C_{12}H_{25}$), 1.44 (s, $OC(CH_3)_3$, 9H), 1.25 (bs, $CH_{2(C14\ Alkyl\ chain)}$, 22H), 0.88 (t, J = 7.2 Hz,CH_3, 3H); HRMS *m/z*: 456.411 (M^+ observed), 456.416 (M^+ calculated).

General procedure for synthesis of 1c-10c: Compounds **1b-10b** were dissolved in 1:1 solution of 4 N HCl and methanol and the mixture was stirred at room temperature for 4 h. The solvents were then evaporated under reduced pressure to obtain pure product in quantitative yield.

3-amino-*N*-benzyl-*N*,*N*-dimethylpropan-1-aminium chloride (1c): ^{1}H-NMR (400 MHz, DMSO-d6) δ/ppm: 8.06 (s, 3H; NH_3^+), 7.60-7.52 (m, 5H; H_{Ar}), 4.57 (s, 2H; $CH_{2(Ar)}NMe_2^+$), 3.39-3.35 (m, 2H; $CH_2NMe_2^+$), 2.99 (s, 6H; $N^+(CH_3)_2$), 2.90 (s, 2H; NH_2-CH_2-CH_2), 2.13-2.09 (m, 2H; NH_2-CH_2-CH_2).

***N*,*N*-dimethyl-*N*-(4-(trifluoromethyl)benzyl)propane-1,3-diaminium chloride (2c):** ^{1}H-NMR (400 MHz, DMSO-d6) δ/ppm: 8.23 (s, 3H; NH_3^+), 7.89 (s, 4H; H_{Ar}), 4.76 (s, 2H; N^+ CH_2Ph), 3.5-3.45 (m, 2H; $CH_2CH_2NMe_2^+$), 3.08 (s, 6H; $N^+(CH_3)_2$), 2.95-2.92 (m, 2H; $^+NH_3$-CH_2-CH_2), 2.16-2.2 (m, 2H; $^+NH_3$-CH_2-CH_2).

***N*,*N*-dimethyl-*N*-(4-methylbenzyl)propane-1,3-diaminium chloride (3c):** ^{1}H-NMR (400 MHz, DMSO-d6) δ/ppm: 8.06 (s, 3H; NH_3^+), 7.51-7.59 (m, 5H; H_{Ar}), 4.57 (s, 2H; N^+ CH_2Ph), 3.35-3.39 (m, 2H; $CH_2CH_2NMe_2^+$), 2.99 (s, 6H; $N^+(CH_3)_2$), 2.89-2.9 (m, 2H; $^+NH_3CH_2CH_2$), 2.09-2.13 (m, 2H; $^+NH_3$-CH_2-CH_2).

***N*-([1,1'-biphenyl]-4-ylmethyl)-3-amino-*N*,*N*-dimethylpropan-1-aminium chloride (4c**): ^{1}H-NMR (400 MHz, DMSO-d6) δ/ppm: 7.98 (s, 3H; NH_3^+), 7.87 (d, *J* = 8.0 Hz, 2H; H_{Ar}), 7.77(d, *J* = 7.2 Hz, 2H; H_{Ar}), 7.71 (d, *J* = 8.4 Hz, 2H; H_{Ar}), 7.56 (t, *J* = 7.6 Hz, 2H; H_{Ar}), 7.46 (t, *J* = 7.6 Hz, 1H; H_{Ar}), 4.63 (s, 2H; N^+CH_2CONH), 3.43-3.39 (m, 2H; $CH_2NMe_2^+$), 3.06 (s, 6H; $N^+(CH_3)_2$), 2.96-2.91 (m, 2H; NH_2-CH_2-CH_2), 2.18-2.1 (m, 2H; $NH_2CH_2CH_2$).

3-Amino-*N*,*N*-dimethyl-*N*-(naphthalen-1-ylmethyl)propan-1-aminium chloride (5c): ^{1}H-NMR (400 MHz, DMSO-d6) δ/ppm: 8.53 d, *J* = 8.4 Hz, 1H; H_{Ar}) 8.19 (d, *J* = 8.0 Hz, 2H; H_{Ar}), $^+$), 8.10 (d, *J* = 8.0 Hz, 1H; H_{Ar}), 7.89 (d, *J* = 6.8 Hz, 1H; H_{Ar}), 7.74-7.64 (m, 3H; NH_2 & H_{Ar}), 5.12 (s, 2H; N^+CH_2CONH), 3.64-3.62 (m, 2H; $CH_2NMe_2^+$), 3.06 (s, 6H; $N^+(CH_3)_2$), 2.95 (bs, 2H; $NH_2CH_2CH_2$), 2.23 (bs, 2H; NH_2-CH_2-CH_2).

3-Amino-*N*,*N*-dimethyl-*N*-(2-oxo-2-(hexylamino)ethyl)propan-1-aminium chloride (6c): ^{1}H-NMR (400 MHz,CD_3OD) δ/ppm: 8.48 (bs, 1H; CON*H*), 4.15 (s, 2H; N^+CH_2CONH), 3.72 (t, *J* = 8.4 Hz, 2H; $NH_2(CH_2)_2CH_2N^+$), 3.33 (s, 6H; $N^+(CH_3)_2$), 3.25-3.21 (m,2H; $CONHCH_2C_5H_{11}$), 3.04 (t, *J* = 7.6 Hz, 2H; NH_2CH_2), 2.25-2.18 (t,2H; $N^+CH_2CH_2CH_2NH_2$), 1.56-1.53 (m, 2H; $CONHCH_2CH_2$), 1.32(bs, 6H; $CH_{2(C6\ alkyl\ chain)}$), 0.90 (t, *J* = 6.8 Hz, 3H; CH_3).

3-Amino-*N*,*N*-dimethyl-N-(2-oxo-2-(octylamino)ethyl)propan-1-aminium chloride (7c): ^{1}H-NMR (400 MHz, $CDCl_3$) δ/ppm: 8.64 (bs, 1H; CON*H*), 7.96 (bs, 1H; CH_2NH_2) 4.15 (s, 2H; N^+CH_2CONH), 3.72 (t, *J* = 8.4 Hz, 2H; $NH_2(CH_2)_2CH_2N^+$), 3.33 (s, 6H; $N^+(CH_3)_2$), 3.25-3.21 (m,2H; $CONHCH_2C_7H_{15}$), 3.04 (t, *J* = 7.6 Hz, 2H; NH_2CH_2), 2.25-2.18 (t,2H; $N^+CH_2CH_2CH_2NH_2$), 1.56-1.53 (m, 2H; $CONHCH_2CH_2$), 1.32(bs, 10H; $CH_{2(C8\ alkyl\ chain)}$), 0.90 (t, *J* = 6.8 Hz, 3H; CH_3).

3-Amino-*N*,*N*-dimethyl-N-(2-oxo-2-(decylamino)ethyl)propan-1-aminium chloride (8c): ^{1}H-NMR (400 MHz, $CDCl_3$) δ/ppm: 8.75 (bs, 1H; CON*H*), 8.11 (bs, 3H; $CH_2NH_3^+$) 4.15 (s, 2H; N^+CH_2CONH), 3.62- 3.58 (m, 2H; $NH_2(CH_2)_2CH_2N^+$), 3.20 (s, 6H; $N^+(CH_3)_2$), 3.11 (dd, *J* = 12.4 Hz, 6.4 Hz, 2H; $CONHCH_2C_9H_{19}$), 2.86 (t, *J* = 6.4 Hz, 2H; NH_2CH_2), 2.06- 2.02 (m, 2H; $N^+CH_2CH_2CH_2NH_2$), 1.43 (bs, 2H; $CONHCH_2CH_2$), 1.25 (bs, 14H; $CH_{2(C10\ alkyl\ chain)}$), 0.86 (t, *J* = 6.8 Hz, 3H; CH_3).

3-Amino-*N*,*N*-dimethyl-*N*-(2-oxo-2-(dodecylamino)ethyl)propan-1-aminium chloride (9c): ^{1}H-NMR (400 MHz, $CDCl_3$) δ/ppm: 8.78 (t, *J* = 5.6 Hz, 1H; CON*H*), 8.15 (s, 3H; $CH_2NH_3^+$) 4.11 (s, 2H; N^+CH_2CONH), 3.63- 3.59 (m, 2H; $NH_2(CH_2)_2CH_2N^+$), 3.20 (s, 6H;

$N^+(CH_3)_2$), 3.11 (dd, *J* = 12.8 Hz, 6.8 Hz, 2H; $CONHCH_2C_{11}H_{23}$), 2.86 (t, *J* = 6.8 Hz, 2H; NH_2CH_2), 2.09- 1.99 (m, 2H; $N^+CH_2CH_2CH_2NH_2$), 1.44-1.43 (bs, 2H; $CONHCH_2CH_2$), 1.24 (bs, 18H; $CH_{2(C12\ alkyl\ chain)}$), 0.85 (t, *J* = 6.8 Hz, 3H; CH_3).

3-Amino-*N*,*N*-dimethyl-*N*-(2-oxo-2-(tetradecylamino)ethyl)propan-1-aminium chloride (10c): ^{1}H-NMR (400 MHz, $CDCl_3$) δ/ppm: 8.78 (t, *J* = 5.6 Hz, 1H; CON*H*), 8.14 (bs, 3H; $CH_2NH_3^+$) 4.11 (s, 2H; N^+CH_2CONH), 3.62- 3.59 (m, 2H; $NH_2(CH_2)_2CH_2N^+$), 3.20 (s, 6H; $N^+(CH_3)_2$), 3.11 (d, *J* = 6.0 Hz, 2H; $CONHCH_2C_{13}H_{27}$), 2.86 (s, 2H; NH_2CH_2), 2.06- 2.03 (m, 2H; $N^+CH_2CH_2CH_2NH_2$), 1.43 (bs, 2H; $CONHCH_2CH_2$), 1.24 (bs, 22H; $CH_{2(C14\ alkyl\ chain)}$), 0.86 (t, *J* = 6.8 Hz, 3H; CH_3).

General procedure for synthesis of vancomycin carboxamides: Vancomycin hydrochloride (100 mg, 1 equivalent) was dissolved in 4 mL 1:1 DMSO: DMF. The reaction mixture was cooled to 0 °C and 220 μL (1.5 equivalents) of 0.45 M HBTU solution in DMF and 5.0 equivalents of diisopropylethylamine (DIPEA) were added. To this, two equivalents of the cationic lipophilic precursor amine (**1c**-**10c**) dissolved in 1 mL 1:1 DMSO:DMF was added. The reaction mixture was continued at room temperature for 12 h. The product was purified by preparative reverse-phase HPLC using 0.1 % trifluoroacetic acid in H_2O/acetonitrile mixture and then lyophilized to obtain tris-(trifluoroacetate) salts of the vancomycin derivatives with more than 90 % purity (65-75 % yield).

VanQph (1): Yield 65%; ^{1}H NMR (DMSO-d6, 400 MHz) 9.4 (br, 1H), 9.12 (d, 1H), 8.78-8.51 (br, 1H), 8.39-8.19 (m, 1H), 7.97-7.91 (m, 1H), 7.97-7.91 (m, 2H), 7.59-7.43 (m, 9H), 7.39-7.33 (m, 3H), 6.94-6.69 (m, 1H), 5.94 (br, 1H), 5.58 (s, 1H), 5.47-5.15 (m, 4H), 4.93 (s, 1H), 4.52-4.4 (m, 2H), 4.3 (d, 1H), 3.69 (d, 2H), 3.59-3.49 (m, 4H), 3.19 (s, 3H), 2.89 (d, 4H), 2.69 (t, 1H), 2.55 (d, 4H), 2.03-1.86 (m, 2H), 1.77-1.69 (m, 1H), 1.69-1.59 (m, 2H), 1.69-1.59 (m, 2H), 1.57-1.47 (m, 1H), 1.3 (s, 2H), 1.23 (s, 1H), 1.07 (d, 3H), 0.93 (d, 3H), 0.84 (d, 3H); HRMS *m/z:* 812.2978 (Observed), 812.3001 ($[M+H]^{2+}$, Calculated for $C_{78}H_{95}Cl_2N_{11}O_{23}^{2+}$).

VanQphCH$_3$ (2): Yield 70%; ^{1}H NMR (DMSO-d6, 400 MHz) 9.38 (s, 1H), 9.13(s, 1H), 8.72 (s, 1H), 8.58 (d, 2H), 8.22 (s, 1H), 7.83 (m, 1H), 7.68 (s, 1H), 7.4-7.28 (m, 3H), 6.92-6.69 (m, 2H), 5.98 (d, 1H), 5.86 (d, 1H), 5.76 (d, 1H), 5.51-5.45 (m, 1H), 5.40-5.30 (m, 2H), 4.73(s, 1H), 4.3 (d, 2H), 4.03 (m, 1H), 3.95 (d, 2H), 3.7 (d, 1H), 3.59-3.49 (m, 3H), 3.2 (m, 2H), 3.09-3.0 (m, 2H), 2.96 (s, 6H), 2.69-2.59 (m, 2H), 2.58-2.53(m, 2H), 2.19-2.04 (m, 1H), 2.02-1.84 (m, 2H), 1.77-1.5 (m, 4H), 1.35-1.22 (m, 2H), 1.07 (d, 2H), 0.95-0.81 (m, 6H); HRMS *m/z:* 819.8066 (observed), 819.8048 ($[M+H]^{2+}$, Calculated for $C_{79}H_{97}Cl_2N_{11}O_{23}^{2+}$).

VanQphCF$_3$ (3): Yield 68%; ^{1}H NMR (DMSO-d6, 400 MHz) 9.38 (s, 1H), 9.16-8.94 (m, 3H), 8.73 (s, 1H), 8.59 (d, 2H), 8.23 (s, 1H), 7.96-7.79 (m, 6H), 7.73-7.44 (m, 6H), 7.4-7.18 (m, 2H), 7.17-6.99 (m, 2H), 6.89 (d, 1H), 6.78 (d, 1H), 6.7 (d, 1H), 6.55 (bs, 1H), 6.38 (d, 1H), 6.24 (d, 1H), 5.97 (d, 1H), 5.86 (d, 1H), 5.76 (d, 1H), 5.65-5.56 (m, 1H), 5.52-5.44 (d, 1H), 5.41-5.14 (m, 4H), 4.93(s, 1H), 4.61-4.48 (m, 2H), 4.3 (d, 2H), 4.03 (m, 1H), 3.95 (d, 2H), 3.7 (d, 1H), 3.59-3.49 (m, 3H), 3.2 (m, 2H), 3.09-3.0 (m, 2H), 2.98 (s, 6H), 2.69-2.59 (m, 2H), 2.58-2.53(m, 2H), 2.19-2.04 (m, 1H), 2.02-1.84 (m, 2H), 1.77-1.5 (m, 4H), 1.35-1.22 (m, 2H), 1.07 (d, 2H), 0.95-0.81 (m, 6H). HRMS *m/z:* 846.7924 (observed), 846.7955 ($[M+H]^{2+}$, Calculated for $C_{79}H_{94}Cl_2F_3N_{11}O_{23}{}^{2+}$).

VanQbiph (4): Yield 66%; ^{1}H NMR (DMSO-d6, 400 MHz) 9.38 (s, 1H), 9.17-8.93 (m, 4H), 8.73 (s, 1H), 8.57 (d, 2H), 8.23 (bs, 1H), 7.83 (d, 3H), 7.74 (d, 2H),7.66 (d, 4H), 7.6-7.39 (m, 7H), 7.38-7.27 (m, 2H), 7.2 (d, 1H), 7.06 (s,1H), 6.93-6.84 (d, 1H), 6.79 (d, 1H), 6.7 (d, 1H),6.51 (bs, 1H), 6.38 (d, 1H), 6.25 (d, 1H), 5.98 (d, 1H), 5.87 (d, 1H), 5.77 (d, 1H), 5.59 (s, 1H), 5.49 (d, 1H), 5.35 (d, 2H), 5.29-5.22 (m, 2H), 5.2 (2H, s), 5.11 (s, 1H), 4.94 (d, 1H), 4.68 (d, 1H), 4.49 (s, 3H), 4.3 (d, 2H), 3.97 (d, 2H), 3.7 (d, 1H), 3.59-3.47 (m, 3H), 3.28 (s, 3H), 3.2 (d, 2H), 3.11 (d, 1H), 2.93 (s, 6H), 2.65 (s, 3H), 2.33 (t, 1H), 2.15 (t, 1H), 2.0 (s, 2H), 1.9 (d, 1H), 1.77-1.52 (m, 4H), 1.31 (s, 3H), 1.24 (s, 1H), 1.08 (d, 2H), 0.95-0.83 (m, 6H). HRMS *m/z:* 850.8119 (observed), 850.8126 ($[M+H]^{2+}$, Calculated for $C_{84}H_{99}Cl_2N_{11}O_{23}{}^{2+}$).

VanQnaph (5): Yield 75%; ^{1}H NMR (DMSO-d6, 400 MHz, 298 K) 9.37 (s, 1H), 9.19-8.93 (m, 3H), 8.78-8.3 (s, 1H), 8.58-8.44 (m, 2H), 8.29-8.03 (m, 3H), 7.76-7.4 (m, 10H), 7.38-7.12 (m, 2H), 7.06 (s,1H), 6.84-6.68 (m, 2H), 6.51 (s, 1H), 6.39 (d, 1H), 6.26 (d, 1H), 5.95 (d, 1H), 5.81-5.75 (m, 1H), 5.63-5.56 (m, 1H), 5.41-5.31 (m, 1H), 5.29-5.22 (m, 3H), 5.29-5.14 (m, 3H), 5.08 (s, 1H), 5.03-4.89 (m, 2H), 4.74-4.65 (m, 1H), 4.51 (s, 1H), 4.38-4.18 (m, 2H), 3.96 (m, 1H), 3.75-3.66 (m, 1H), 3.59-3.51 (m, 2H), 3.27 (s, 2H), 3.27 (d, 2H), 3.21-3.08 (m, 2H), 3.09 (s, 1H), 2.97-2.84 (m, 7H), 2.69-2.59 (m, 2H), 2.57-2.53 (m, 2H), 2.33 (m, 1H), 2.21-1.98 (m, 3H), 1.94-1.86 (m, 1H), 1.79-1.51 (m, 4H), 1.3 (s, 3H), 1.07 (d, 2H), 0.95-0.83 (m, 6H). HRMS *m/z:* 837.8053 (Observed), 837.8096 ($[M+H]^{2+}$, Calculated for $C_{82}H_{97}Cl_2N_{11}O_{23}{}^{2+}$).

VanQAmC$_6$ (6): Yield 75%; HPLC purity 98%; ^{1}H-NMR (400 MHz, DMSO-d6) δ/ppm: 9.37 (s, 1H), 9.18-8.96 (m, 3H), 8.74-8.48 (m, 4H), 8.21 (bs, 1H), 7.82 (s, 1H), 7.67 (bs, 3H),7.6-7.43 (m, 4H), 7.38-7.33 (d, 1H), 7.32-7.26 (d, 1H), 7.22-7.18 (d, 1H), 7.08-7.01 (m,1H), 6.96-6.89 (d, 1H), 6.8-6.76 (d, 1H), 6.73-6.69 (d, 1H), 6.39-6.37 (d, 1H), 6.24-6.21 (d, 1H), 6-5.96 (m, 1H), 5.87-5.82 (d, 1H), 5.79-5.74 (d, 1H), 5.59 (s, 1H), 5.49-5.44 (d, 1H), 5.39-5.17 (m,

5H), 4.97-4.9 (d, 1H), 4.7-4.65 (m, 1H), 4.5-4.47 (d, 1H), 4.3-4.23 (d, 2H), 4.07-3.9 (m, 4H), 3.72-3.65 (d, 1H), 3.57-3.42 (m, 3H), 3.29-3.25 (s, 2H), 3.19 (s, 1H), 3.12-3.09 (m, 7H), 2.65 (bs, 2H), 2.51-2.49 (m, 12H), 2.19-2.12 (m, 1H), 2.19-2.12 (m, 1H), 1.93-1.82 (m, 2H), 1.75-1.52 (m, 3H), 1.45-1.38 (m, 2H), 1.32-1.29 (s, 2H), 1.27-1.24 (m, 6H),1.09-1.05 (d, 2H), 0.92-0.83 (m, 9H). HRMS *m/z:* 837.3299 (Observed), 837.3326 ($[M+H]^{2+}$, Calculated for $C_{79}H_{103}Cl_2N_{12}O_{24}^{2+}$).

VanQAmC8 (7): Yield 73%; HPLC purity 97%; ^{1}H-NMR (400 MHz, DMSO-d6) δ/ppm: 9.37 (s, 1H), 9.19-9.03 (m, 3H), 8.75-8.47 (m, 4H), 8.18 (bs, 1H), 7.98 (s, 1H), 7.82 (bs, 2H), 7.68 (s, 2H), 7.58-7.44 (m, 3H), 7.36-7.33 (d,1H), 7.29 (s, 1H), 7.21-7.17 (d, 1H), 7.03 (bs, 1H), 6.93-6.9 (1H,d), 6.8-6.76 (d, H), 6.4-6.37 (d, 1H), 6.23-6.2 (d, 1H), 5.98 (s, 1H), 5.78-5.74 (d, 1H), 5.59 (s, 1H), 5.39-5.14 (m, 5H), 4.97-4.9 (d, 1H), 4.7-4.65 (m, 1H), 4.5-4.47 (d, 1H), 4.3-4.23 (d, 2H), 4.3-4.23 (d, 2H), 4.06-3.92 (m,3H), 3.72-3.66 (d, 1H), 3.59-3.51 (m,2H), 3.26 (s, 2H), 3.17 (s, 4H), 3.13-3.09 (m, 7H), 2.89-2.84 (t, 1H), 2.65 (s, 2H), 2.5-2.49 (m, 12H), 2.20-2.11 (m, 1H),2-1.95 (m, 2H), 1.94-1.1.8 (m,2H), 1.76-1.54 (m, 3H), 1.46-1.38 (t, 2H), 1.31-1.28 (m, 2H), 1.27-1.22 (bs, 10H), 1.1-1.05 (d, 2H), 0.92-0.84 (m, 9H). HRMS *m/z*: 852.3447 (observed), 852.3468 ($[M+H]^{2+}$, Calculated $C_{81}H_{108}Cl_2N_{12}O_{24}^{2+}$).

VanQAmC10 (8): Yield 69%; HPLC purity 98%; ^{1}H-NMR (400 MHz, DMSO-d6) δ/ppm: 9.37 (s, 1H), 9.24-8.95 (m, 3H), 8.77-8.49 (m, 4H), 8.18 (bs, 1H),8—7.94 (m, 1H), 7.99 (s, 1H), 7.82 (bs, 2H), 7.68 (m, 3H), 7.59-7.44 (m, 3H), 7.38-7.28 (m, 2H), 7.22-7.19 (d, 1H), 7.05 (s, 1H), 6.95-6.89 (d, 1H), 6.8-6.76 (d, 1H), 6.73-6.68 (d, 1H), 6.39-6.37 (d, 1H), 6.23-6.21 (d, 1H), 6.01-5.97 (s, 1H), 5.85 (s,1H), 5.79-5.74 (d, 1H), 5.59 (s, 1H), 5.47 (s, 1H), 5.4-5.17 (m, 5H), 4.97-4.92 (d, 1H), 4.72-4.66 (d, 1H), 4.52-4.47 (d, 1H), 4.32-4.22 (d, 2H), 4.07-3.89 (m, 3H), 3.72-3.66 (d, 1H), 3.59-3.45 (m, 3H), 3.29-3.25 (s, 2H), 3.18 (s, 3H), 3.14 (bs, 7H), 2.9-2.83 (m, 1H), 2.65 (s, 2H), 2.52-2.48 (m, 13H), 2.2-2.12 (m, 1H), 2.04-1.96 (m, 1H), 1.95-1.8 (m, 2H), 1.76-1.53 (m, 3H), 1.45-1.38 (m, 2H), 1.32-1.29 (m, 2H), 1.26-1.22 (m, 14H), 1.08-1.05 (d, 2H), 0.92-0.83 (m, 9H); HRMS *m/z*: 866.3623 (Observed), 866.3625 ($[M+H]^{2+}$, Calculated $C_{83}H_{112}Cl_2N_{12}O_{24}^{2+}$).

VanQAmC12 (9): Yield 70%; HPLC purity 95%; ^{1}H-NMR (400 MHz, DMSO-d6) δ/ppm: 9.36 (s, 1H), 9.17-8.98 (m, 3H), 8.75-8.47 (m, 4H), 8.16 (s, 1H), 7.82 (s, 2H), 7.77 (s, 1H), 7.68 (s, 3H), 7.59-7.43 (m, 4H), 7.39-7.26 (m, 2H), 7.22-7.17 (d, 1H), 7.05 (s, 1H), 6.93-6.88 (d, 1H), 6.8-6.76 (m, 1H), 6.72-6.68 (d, 1H), 6.4-6.36 (d, 1H), 6.24-6.21 (d, 1H), 6-5.95 (d, 1H), 5.86-5.8 (d, 1H), 5.79-5.74 (d, 1H), 5.59 (s, 1H), 5.48-5.44 (d, 1H), 5.38-5.16 (m, 5H), 4.93 (s, 1H),

4.72-4.62 (d, 1H), 4.5-4.46 (d, 1H), 4.31-4.22 (d, 2H), 4.05-3.91 (bs, 3H), 3.73-3.65(m, 1H), 3.59-3.43 (m, 3H), 3.29-3.24 (s, 2H), 3.18(m, 2H), 3.12 (bs, 7H), 2.64 (s, 2H), 2.51-2.48 (m, 13H), 2.19-2.11 (m, 1H), 1.95-1.8 (m, 2H), 1.76-1.54 (m, 2H), 1.39-1.46(m, 2H), 1.28-1.2 (bs, 18H), 1.09-1.05 (d, 2H), 0.92-0.83 (m, 3H); HR-MS *m/z:* 879.3804 (Observed), 879.3796 ($[M+H]^{2+}$,Calculated $C_{85}H_{116}Cl_2N_{12}O_{24}^{2+}$).

VanQAmC$_{14}$ (10): Yield 65%; HPLC purity 96%; ^{1}H-NMR (400 MHz, DMSO-d6) δ/ppm: 9.37 (s, 1H), 9.19-9.03 (m, 3H), 8.76-8.47 (m, 4H), 8.18 (bs, 1H), 7.98 (s, 1H), 7.82 (bs, 2H), 7.68 (s, 2H) 7.58-7.44 (m, 3H),7.36-7.33 (d,1H),7.29 (s, 1H), 7.21-7.17 (d, 1H), 7.03 (bs, 1H), 6.93-6.9 (1H,d), 6.8-6.76 (d, 1H), 6.4-6.37 (d, 1H), 6.23-6.2 (d, 1H), 5.98 (s, 1H), 5.78-5.74 (d, 1H), 5.59 (s, 1H), 5.39-5.14 (m, 5H), 4.97-4.9 (d, 1H), 4.7-4.65 (m, 1H), 4.5-4.47 (d, 1H), 4.3-4.23 (d, 2H), 4.3-4.23 (d, 2H), 4.06-3.92 (m, 3H), 3.72-3.66 (d, 1H), 3.59-3.51 (m, 2H), 3.26 (s, 2H), 3.17 (s, 4H), 3.13-3.09 (m, 7H), 2.89-2.84 (t, 1H), 2.65 (s, 2H), 2.5-2.49 (m, 12H), 2.20-2.11 (m, 1H), 2-1.95 (m, 2H), 1.94-1.1.8 (m, 2H), 1.76-1.54 (m, 3H), 1.46-1.38 (m, 2H), 1.34-1.29 (m, 2H),1.28-1.22 (bs, 22H), 1.11-1.05 (d, 2H), 0.92-0.84 (m, 9H); HRMS *m/z:* 893.3912 (Observed), 894.3952 ($[M+H]^{2+}$,Calculated $C_{87}H_{120}Cl_2N_{12}O_{24}^{2+}$).

Biological assays

Determination of MIC [2]

Test compound was assayed in a micro-dilution broth format as per the CLSI guideline. The bacterial freeze dried stock samples were stored at -80ºC. About 5 µL of these stocks were added to 3 mL of the respective broth and the culture was grown for 6 h at 37°C with prior to the experiments. This 6 h grown culture was diluted to give effective cell concentration of 10^5 CFU/mL which was then used for determining MIC. Compounds were serially diluted 2-fold, in sterile millipore water and 50 µL of these serial dilutions were added to the wells of 96 well plate followed by the addition of about 150 µL of bacterial solution. The plates were then incubated for 18-24 h at 37ºC. The O.D. value at 600 nm was recorded using TECAN (Infinite series, M200 pro) Plate Reader. Each concentration had triplicate values and the whole experiment was done at least twice and the MIC value was determined by taking the average of triplicate O. D. values for each concentration and plotting it against concentration. The data was then subjected to sigmoidal fitting. From the curve the MIC value was determined, as the point where the O. D. was similar to that of control having no bacteria.

Haemolysis assay[3]

50 µL of serially diluted compound was added into 96 well microtiter plates. Freshly drawn, heparanized human blood was centrifuged down to obtain the erythrocytes and re-suspended to 5 vol % in PBS (pH 7.4). 150 µL of the erythrocyte suspension was added to the serially diluted compounds (from 500 µM to 2 µM). One set without compound and other with 50 µL of 1 vol % solution of Triton X-100 were kept as controls. The plates were incubated at 37 °C for 1 h followed by centrifugation at 3,500 rpm for 5 min. 100 µL of the supernatant from each well were transferred into fresh microtiter plates, and A_{540} was measured.

% haemolysis = $(A - A_0)/(A_{total} - A_0) \times 100$, where A is the absorbance of the test well, A_0 the absorbance of the negative controls (without compound), and A_{total} the absorbance of 100 % hemolysis wells (with Triton X-100), all at 540 nm.

Cytotoxicity assay (Alamar-blue assay)

The standard protocol as mentioned in the kit was followed. Briefly, 2×10^4 MDCK (NBL-2) - CCL-34 cells were seeded per well in 100 µL of DMEM media in a 96-well plate and incubated for 24 h. VanQAmC_{10} were treated at various at 2-fold dilution concentrations from 64 µM to 1 µM to and incubated for 24 h after which 10 µL of 10× Alamar blue dye was added. 2 h post incubation with dye, the absorbance was measured at 570 nm using 600 nm as reference wavelength.

Time-kill kinetics assay[4]

The bactericidal activity of the compounds was evaluated with the time kill kinetics assay. MRSA ATCC 33591 cells were cultured in nutrient media at 37°C for 6 h. VanQAmC_{10}, VanQbiph, and vancomycin was added to the bacterial solution (~1.8×10^5 CFU/mL) with the working concentration of 25 µM. This was incubated at 37°C. At different time intervals (0, 1, 2, 3, 6 and 24 h) 20 µL of aliquots from that solution were serially diluted 10-fold in 0.9 % saline. Then from the dilutions, 20 µL was plated on yeast-dextrose agar plates and incubated at 37°C for 24 h. The bacterial colonies were counted and results are represented in logarithmic scale, i.e. $\log_{10}$ (CFU/mL).

Membrane permeabilization assay[5]

The 6 h grown culture (mid log phase) of MRSA were harvested (5000 rpm, 5 min), washed, and resuspended in 1:1 solution of 5 mM glucose and 5 mM HEPES buffer at pH 7.2. 10 µM of propidium iodide (PI) was added to the bacterial suspension and 200 µL of this mixture was put into 96-well flat clear bottomed black well plates. 20 µL of test compound and vancomycin

were added to it the wells containing bacterial suspension. Fluorescence has monitored at excitation wavelength of 535 nm (slit width: 10 nm) and emission wavelength of 617 nm (slit width: 5 nm). As a measure of inner membrane permeabilization the uptake of PI was monitor by the increase in fluorescence for 30-50 min. For assessing depolarisation against stationary phase cells of MRSA ATCC 33591, cells were grown to the stationary phase for 16 h. The culture was then diluted to an OD_{600} of 0.2 and the depolarisation assays was performed as described. The experiments were performed three times independently in duplicates and the graph is representative of the results from all experiments.

Cytoplasmic membrane depolarization assay[5]

Mid log phase MRSA were harvested (3500 rpm, 5 min), washed in 5 mM glucose and 5 mM HEPES buffer (pH 7.2) in 1:1 ratio and resuspended in 5 mM HEPES buffer, 5 mM glucose and 100 mM KCl solution in 1:1:1 ratio. Then 2 µM of 3, 3′-Dipropylthiadicarbocyanine iodide (DiSC3(5)) was added to bacterial suspension and pre-incubated for 45 min. The fluorescence was monitored at excitation wavelength of 622 nm and emission wavelength of 670 nm. Then 20 µL of test compound and vancomycin were added to black well plates containing bacterial suspension and DiSC3(5) after 2-4 min of fluorescence measurement. As a measure of membrane depolarization fluorescence was monitor for another 35 min. For assessing membrane permeabilization against stationary phase cells of MRSA ATCC 33591, cells were grown to the stationary phase for 16 h. The culture was then diluted to an OD_{600} of 0.2 and the depolarisation assays was performed as described. The images are representative of results from three independent experiments done in triplicates

Growth of mature biofilms of MRSA

Biofilms were grown as per previously reported protocol.[6] A mid log phase culture of MRSA was diluted to a concentration of approximately 10^5 CFU/mL in a nutrient broth supplemented with 1% w/v glucose and 1% w/v NaCl to make the bacterial stock solution. Sterile glass cover slips of 18 mm diameter were placed in 6-well plates individually. Biofilms of MRSA were then allowed to form on the glass cover-slip by incubating the bacterial solution at 37 ˚C for 24 h. After 24 h, the cover slips containing mature biofilms were removed and carefully washed with 0.9% saline to remove planktonic bacteria. The biofilm containing cover-slips were then taken into a fresh well in a 6-well plate and treated with 2 mL complete nutrient media containing the test compounds (vancomycin, $VanQAmC_{10}$) at the concentration of 20 µM each. Glass cover slips 24 h post-treatment with the test compounds at the mentioned concentrations,

and the untreated control were carefully removed from the well, washed with 0.9 % saline, and placed on glass slides. The biofilms were then stained with 10 µL of SYTO9 (60µM) and imaged using a Zeiss 510 Meta confocal laser-scanning microscope. The orthogonal projections of the images were processed with LSM 5 Image examiner. The experiment was performed three times independently and the image provided is representative of these.

Determination of MIC against *B. subtilis*

Overnight cultures of *B. subtilis* 168 were grown in 50 mL flasks in 5 mL BMM media. The bacteria in the mid-log phase were then incubated with compounds for 18 h at 37 ℃ in BMM media. The MIC was determined as the lowest concentration at which no visible bacterial OD was observed.

Determination of Physiologically Effective Concentration[7]

Overnight cultures of *B. subtilis* 168 were grown in 50 mL flasks with 10 mL of media. Upon growing to mid-log phase (an OD_{500} between 0.5 and 1), bacteria was inoculated in 100mL BMM in a 500 ml flask. When the cultures reach an OD_{500} of 0.35, the bacteria is aliquoted into separate conicals (50 mL) with 5 ml and compounds are added. The O.D. of the solutions are recorded every 30 minutes. The concentration which retards the growth of bacteria was selected as the PEC. The experiments were performed two times independently and the images are representative of results from two independent experiments. The experiment was done together with the compound published in (*J. Med. Chem.* 64, **2021,** 10185) and therefore the graph for vancomycin is the same.

Cell wall biosynthesis inhibition assay or Bubble assay

Overnight bacterial cultures were inoculated in BMM and allowed to grow to an OD 500 of 0.35. 200 µL of the bacterial culture were incubated with compounds at 37 ℃ for 15 minutes. Post treatment, the cells were fixed with 1 mL of 1:3 Acetate/methanol.[7] The morphology of the cells were then examined through microscopy. The experiments were performed three times independently and the images are representative of results from three independent experiments.

BAC Light assay or pore formation assay

Overnight bacterial cultures were inoculated in BMM and allowed to grow to an OD 500 of 0.35. 500 µL of the bacteria was then incubated with the compounds at their respective PECSs for 10 mins in the same media at 37 ℃. The compound was then centrifuged at 13200 rpm and

resuspended in prewarmed BMM media. 1:1 BAC light dye was then added to the bacterial suspension and incubated for 5 minutes. The fluorescence in the GFP and Texas red channels was then observed under the microscope. The experiments were performed three times independently and the images are representative of results from three independent experiments.

GFP-MinD localization

B. subtilis 1981 GFP-MinD was cultured overnight in BMM.[8] Cells were then inoculated in xylose containing BMM instead of glucose to an OD_{500} of 0.1 to induce expression of the GFP-MinD fusion protein. Upon reaching an OD_{500} of 0.35, the cells were treated with test compounds at the PEC (vancomycin, $VanQAmC_{10}$, VanQbiph) and 0.75 µg/mL (nisin) for 15 min. 0.5 µl of nonfixed, non-immobilized samples of the culture were imaged immediately in fluorescent mode (Olympus microscope with a U-LH100HGAPO burner and a U-RFL-T power supply). The experiments were performed three times independently and the images are representative of results from three independent experiments.

GFP-FtsI and GFP-FtsZ localization in *E. coli*

E. coli MG1655 strains pDSW230 and pJW234 overexpressing *FtsZ-gfp* and *FtsI-gfp* respectively were cultured overnight in LB media containing ampicillin at 100µg/ml at 37℃. Cells were then inoculated in LB media at 1:100 dilution ratio and made to grow till $O.D_{600}$ 0.1. Cells were then induced for 40 minutes with 2.5µm isopropyl--D-thiogalactopyranoside (IPTG) for GFP-FtsI and for 2 hours with 5µm IPTG for GFP-FtsZ. Along with induction with IPTG, cells were simultaneously treated with drugs vancomycin, $VanQAmC_{10}$ or left untreated. About 6 µl of sample was immobilized on 1% agarose pad. DIC and fluorescence images were obtained at different time intervals using Nikon Eclipse Ti Microscope equipped with Nikon DS-U3 camera through Plan Apo 100×/1.40 oil objective. All images were taken at 100× or 60× magnification and were optically zoomed 1.5× whenever required. Against GFP-FtsZ expressing *E. coli* vancomycin (20 µM), VanQAmC10 (15 µM), while GFP-FtsI expressing *E. coli* were treated with, vancomycin at 20 µM, VanQAmC10 at 10 and 20 µM. The experiments were performed three times independently and the images are representative of results from three independent experiments.

Antibacterial activity against Δ*amiAC* MG1655 *E. coli*

Δ*amiAC* MG1655 was cultured overnight in LB media at 37℃. Cells were then inoculated in LB media at 1:100 dilution ratio and made to grow till $O.D_{600}$ 0.05. The cells were then treated with varying concentrations of vancomycin and $VanQAmC_{10}$ with 2-fold dilutions starting

from 10 μg/mL. The bacteria with compounds and untreated control were incubated for 18 h at 37℃ under shaking and OD_{600} was measured.

Microscopy of Δ*amiAC* MG1655 *E. coli*

Δ*amiAC* MG1655 was cultured overnight in LB media at 37℃. Cells were then inoculated in LB media at 1:100 dilution ratio and made to grow till $O.D_{600}$ 0.1. The cells were then treated with 4 μg/mL concentration of vancomycin and $VanQAmC_{10}$ or left untreated for 2 h. 6 μl of sample was immobilized on 1% agarose pad and microscopy was performed as described above. The experiments were performed three times independently and the images are representative of results from three independent experiments.

Antibacterial activity against stationary phase bacteria

MRSA ATCC 33591 was grown to mid-log phase at 37 °C. The bacterial suspension was diluted 1000-fold in nutrient media and allowed to reach stationary phase in 16 h. The bacterial suspension was diluted and spot-plated to determine the bacterial count. The 16 h growth culture was diluted to ~10^6 CFU/mL in PBS. 150 μL of this bacterial suspension was then incubated with 50 μL of test compounds in a 96 well-plate. The compound and bacteria were then incubated at 37 °C for 2h. The suspension was serially diluted 10 fold and spot-plated on Mac Conkey agar. The viable bacteria were then counted after 48 h incubation at 37 °C. The results represented are the average of two independent experiments carried in duplicates.

Persister cells were then generated by treating the stationary phase culture of MRSA ATCC 33591 with 100 μg/mL of ampicillin for 3 h.

Antagonization assays

Antagonization of antibacterial activity of AAV-qC10 was determined by addition of 500 μM *N*,*N*′-diacetyl–L-Lys–D-Ala–D-Ala or 100 μg/mL of lipoteichoic acid to serial dilutions of the test compound. The MIC was then determined against MRSA by measuring the OD_{600} 18-24 h post-incubation.

Resistance study

Vancomycin was chosen as control antibiotic for MRSA. The initial MIC values of $VanQAmC_{10}$, vancomycin were determined against the respective bacteria. After the initial MIC experiment, serial passaging was initiated by harvesting bacterial cells growing in the sub-MIC concentration of the compounds and was subjected to another MIC assay.[9] The

bacterial concentration was adjusted to ~ 10^5 CFU/mL based on OD_{600}. The process was repeated for 27 passages. The fold of MIC increased for test compounds were plotted against the number of days.

Activity in blood plasma and liver homogenate

To examine the susceptibility of $VanQAmC_{10}$ to serum proteases, the antibacterial activities was tested in the presence of 50% of plasma and liver homogenate. Briefly, 250 μL of $VanQAmC_{10}$ was added into 250 μL of fresh human plasma and incubated at 37 °C. An aliquot of the samples 3 h and 24h post-incubation was diluted in 0.9% saline and the antibacterial activity (MIC) were determined against MRSA and VRE by following the same protocol as described above for the antibacterial assay.

***In-vivo* toxicity in mice**

The toxicity of the lead compound $VanQAmC_{10}$ was tested in mice through various modes of administration and reported as the LD_{50} (LD = lethal dose). The LD_{50} is the dosage that kills 50% of the test population of mice. Balb/c female mice were divided into groups, with five mice in each group. Each group was given a single injection of various doses of $VanQAmC_{10}$ through intraperitoneal, intravenous and subcutaneous routes respectively, and the survival of mice in each group was observed for 14 days. The doses for administration, were given modified OECD guidelines. LD_{50} was determined using Spearman-Karber method.

***In-vivo* activity in murine thigh infection model**

Groups of four 6 to 8 week-old Balb/c specific-pathogen-free female mice were used (weight ~22 g) for the experiment. The mice were rendered neutropenic by injecting two intraperitoneal doses of cyclophosphamide, 4 days (150 mg kg^{-1}) and 1 day (100 mg kg^{-1}) before the infection experiment. 50 μL of ∼10^6 CFU/mL bacterial inoculum (MRSA ATCC33591) was injected into the thigh. 1 h post-inoculation, animals were treated intraperitoneally twice with 12 h intervals with saline, vancomycin (12 mg kg^{-1}), and $VanQAmC_{10}$ (12 mg kg^{-1}). 24 h post the first treatment, the animals were euthanized (using ether) and the thighs were collected aseptically. The thigh tissue was weighed and homogenized. The dilutions of the homogenate were plated onto agar plates, which were incubated overnight at about 37 °C. The bacterial titer was expressed as Log CFU/g of thigh weight and plotted in GraphPad Prism software.

***In-vivo* activity in murine burn wound infectionmodel [10-12]**

Female Balb/c mice (6-8 weeks, 22-25 g) were anesthetized with a cocktail of ketamine-xylazine and their dorsal surface shaved and cleansed. 6 mm diameter burn wounds were created by applying a 120 s heated brass bar for 10 s. Immediately after injury, burn wounds were infected with a mid-log phase bacterial inoculum of about ~10^6 CFU (20 μL from 0.8×10^8 CFU mL^{-1}) of *A. baumannii* (R674) prepared in PBS. To mimic the condition for the biofilms, burn wounds were left untreated for 24 h. Treatment started 24 h post infection by when the infection has already become established. Burn wounds were treated every 24 h for 5 days. $VanQAmC_{10}$ and vancomycin were dissolved 0.9% saline. 40 μL of solutions (30 mg kg) was treated to burn wounds. Mice were euthanized 6 days post-injury; the wounded muscle tissue was excised, weighed, and homogenized in 10 mL of PBS. Serial dilutions of homogenate were plated on MacConkey agar (Himedia, India) and the results were stated as log ($CFUg^{-1}$) of tissue.

References

1. D. Uppu, M. M. Konai, U. Baul, P. Singh, T. K. Siersma, S. Samaddar, S. Vemparala, L. W. Hamoen, C. Narayana and J. Haldar, *Chem Sci*, 2016, **7**, 4613-4623.
2. I. Wiegand, K. Hilpert and R. E. Hancock, *Nat Protoc*, 2008, **3**, 163-175.
3. V. Yarlagadda, G. B. Manjunath, P. Sarkar, P. Akkapeddi, K. Paramanandham, B. R. Shome, R. Ravikumar and J. Haldar, *ACS Infect. Dis.*, 2016, **2**, 132-9.
4. V. Yarlagadda, P. Akkapeddi, G. B. Manjunath and J. Haldar, *J. Med. Chem.*, 2014, **57**, 4558-4568.
5. C. Ghosh, G. B. Manjunath, P. Akkapeddi, V. Yarlagadda, J. Hoque, D. S. Uppu, M. M. Konai and J. Haldar, *J. Med. Chem.*, 2014, **57**, 1428-1436.
6. C. Ghosh, G. B. Manjunath, M. M. Konai, D. S. Uppu, J. Hoque, K. Paramanandham, B. R. Shome and J. Haldar, *PLoS One*, 2015, **10**, e0144094.
7. M. Wenzel, B. Kohl, D. Munch, N. Raatschen, H. B. Albada, L. Hamoen, N. Metzler-Nolte, H. G. Sahl and J. E. Bandow, *Antimicrob Agents Chemother*, 2012, **56**, 5749-5757.
8. H. Strahl and L. W. Hamoen, *Proc Natl Acad Sci USA*, 2010, **107**, 12281-12286.
9. V. Yarlagadda, S. Samaddar, K. Paramanandham, B. R. Shome and J. Haldar, *Angew. Chem. Int. Ed. Engl.*, 2015, **54**, 13644-13649.
10. M. G. Thompson, C. C. Black, R. L. Pavlicek, C. L. Honnold, M. C. Wise, Y. A. Alamneh, J. K. Moon, J. L. Kessler, Y. Si, R. Williams, S. Yildirim, B. C. Kirkup, Jr., R. K. Green, E. R. Hall, T. J. Palys and D. V. Zurawski, *Antimicrob Agents Chemother*, 2014, **58**, 1332-1342.
11. C. Ghosh, G. B. Manjunath, M. M. Konai, D. S. Uppu, K. Paramanandham, B. R. Shome, R. Ravikumar and J. Haldar, *ACS Infect Dis*, 2016, **2**, 111-122.
12. D. S. Uppu, S. Samaddar, C. Ghosh, K. Paramanandham, B. R. Shome and J. Haldar, *Biomaterials*, 2016, **74**, 131-143.
